# New insight into dyslipidemia‐induced cellular senescence in atherosclerosis

**DOI:** 10.1111/brv.12866

**Published:** 2022-05-15

**Authors:** Qunyan Xiang, Feng Tian, Jin Xu, Xiao Du, Shilan Zhang, Ling Liu

**Affiliations:** ^1^ Department of Geriatrics, The Second Xiangya Hospital Central South University Changsha Hunan 410011 PR China; ^2^ Institute of Aging and Age‐related Disease Research Central South University Changsha Hunan 410011 PR China; ^3^ Department of Geriatric Cardiology The First Affiliated Hospital of Zhengzhou University Zhengzhou Henan 450000 PR China; ^4^ Department of Cardiovascular Medicine, The Second Xiangya Hospital Central South University Changsha Hunan 410011 PR China; ^5^ Research Institute of Blood Lipid and Atherosclerosis Central South University Changsha Hunan 410011 PR China; ^6^ Modern Cardiovascular Disease Clinical Technology Research Center of Hunan Province Changsha Hunan 410011 PR China; ^7^ Cardiovascular Disease Research Center of Hunan Province Changsha Hunan 410011 PR China; ^8^ Department of Gastroenterology, The Second Xiangya Hospital Central South University Changsha Hunan 410011 PR China

**Keywords:** atherosclerosis, endothelial cells, vascular smooth muscle cells, macrophages, adipose‐derived mesenchymal stem cells, senescence, dyslipidemia

## Abstract

Atherosclerosis, characterized by lipid‐rich plaques in the arterial wall, is an age‐related disorder and a leading cause of mortality worldwide. However, the specific mechanisms remain complex. Recently, emerging evidence has demonstrated that senescence of various types of cells, such as endothelial cells (ECs), vascular smooth muscle cells (VSMCs), macrophages, endothelial progenitor cells (EPCs), and adipose‐derived mesenchymal stem cells (AMSCs) contributes to atherosclerosis. Cellular senescence and atherosclerosis share various causative stimuli, in which dyslipidemia has attracted much attention. Dyslipidemia, mainly referred to elevated plasma levels of atherogenic lipids or lipoproteins, or functional impairment of anti‐atherogenic lipids or lipoproteins, plays a pivotal role both in cellular senescence and atherosclerosis. In this review, we summarize the current evidence for dyslipidemia‐induced cellular senescence during atherosclerosis, with a focus on low‐density lipoprotein (LDL) and its modifications, hydrolysate of triglyceride‐rich lipoproteins (TRLs), and high‐density lipoprotein (HDL), respectively. Furthermore, we describe the underlying mechanisms linking dyslipidemia‐induced cellular senescence and atherosclerosis. Finally, we discuss the senescence‐related therapeutic strategies for atherosclerosis, with special attention given to the anti‐atherosclerotic effects of promising geroprotectors as well as anti‐senescence effects of current lipid‐lowering drugs.

## I. INTRODUCTION

Atherosclerosis is a chronic immune–inflammatory, progressive and age‐related disorder, characterized by lipid‐rich plaques in the arterial wall (Libby *et al*., 2009; Savji *et al*., 2013). Despite the continuous therapeutic advances in cardiology, atherosclerosis remains the leading cause of mortality worldwide (GBD 2013 Mortality and Causes of Death Collaborators, 2015). Excessive deposition of pro‐atherogenic lipids and lipoproteins in the arterial *intima* leads to the structural destruction and functional impairment of endothelial cells (ECs), which results in the recruitment and migration of monocytes across the endothelial barrier (Steinberg *et al*., 1989). The migrated monocytes then differentiate into macrophages in order to clear the accumulated lipids and lipoproteins, but transform into foam cells when overloaded, which aggravates the formation and development of atherosclerotic plaques and triggers an inflammation response by releasing pro‐inflammatory factors (Yu *et al*., 2013). Meanwhile, vascular smooth muscle cells (VSMCs) in the arterial *media* also migrate into the arterial *intima*, surrounding the inflammatory factors and lipids (Lugano *et al*., 2013). These highly proliferative VSMCs then form a fibrous cap to stabilize the atherosclerotic plaques on the one hand, while secreting various matrix metalloproteinases (MMPs) that promote plaque rupture on the other hand (Allahverdian *et al*., 2018). VSMCs can transform into a foam‐cell‐like phenotype in some advanced atherosclerotic plaques, which further aggravates the progression and instability of atherosclerotic plaques (Vengrenyuk *et al*., 2015). In addition, endothelial progenitor cells (EPCs), a main source of new ECs, play a vital role in vascular repair and atherosclerosis (Zampetaki, Kirton & Xu, 2008). Reduced number and impaired function of EPCs are important biomarkers and predictors for atherosclerosis (Schmidt‐Lucke *et al*., 2005; Keymel *et al*., 2008). Moreover, perivascular adipose tissue (PVAT), a supporting component surrounding the blood vessels, regulates vascular function and atherosclerosis (Qi *et al*., 2018; Quesada *et al*., 2018). Paracrine secretion of various bioactive factors by adipose‐derived mesenchymal stem cells (AMSCs) may be crucial during this process (Gu *et al*., 2019). Hence, dysfunction of all these types of cells plays a pivotal role in atherosclerosis.

**Fig. 1:**
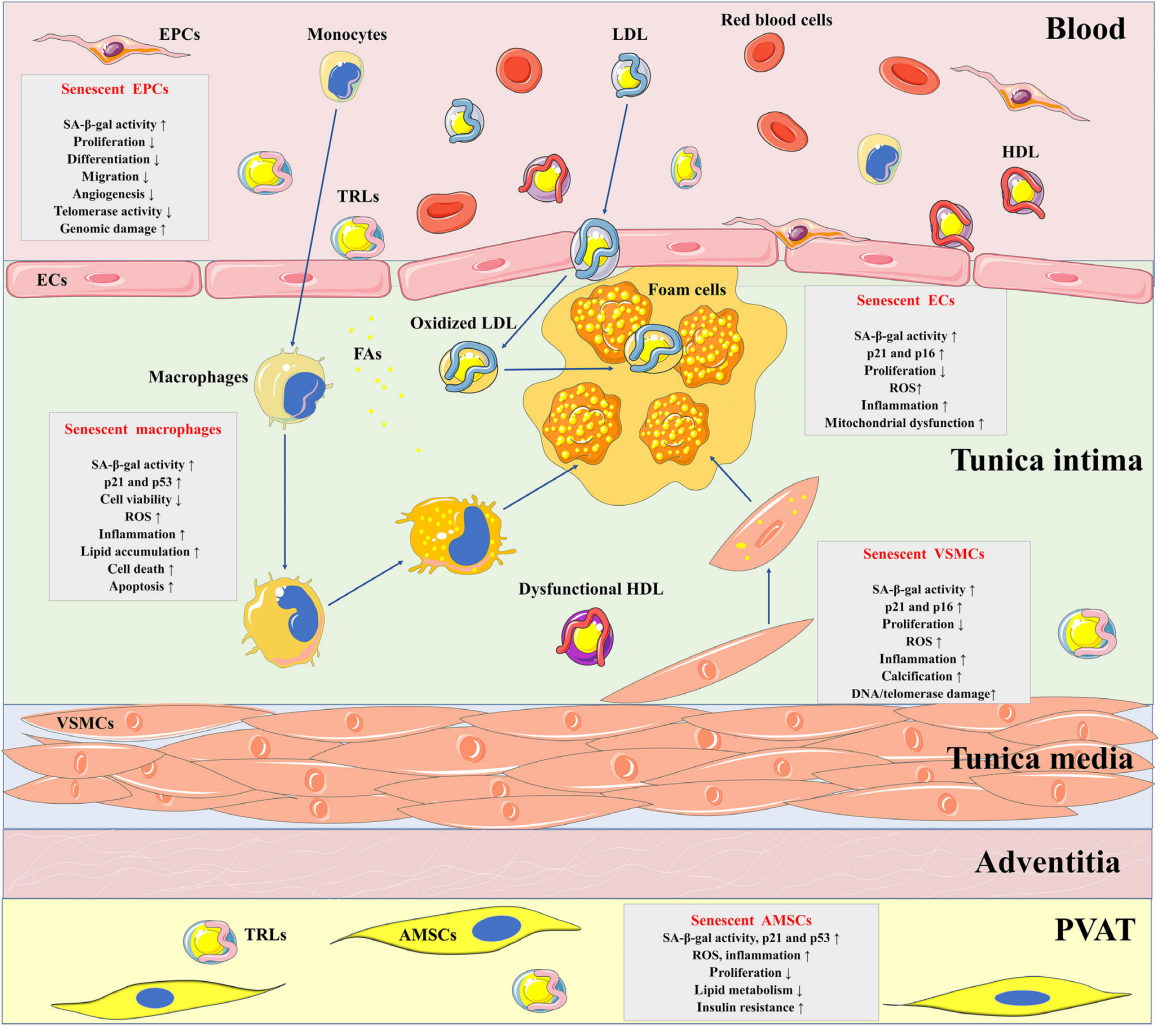
Contributions of dyslipidemia‐induced senescence to atherosclerosis. Dyslipidemia promotes the development and progression of atherosclerosis through multiple mechanisms, one of which might be related to cellular senescence. The main categories of pro‐atherogenic lipids and lipoproteins include modified low‐density lipoprotein (LDL), triglyceride‐rich lipoproteins (TRLs) and its hydrolysate, and dysfunctional high‐density lipoprotein (HDL). They can lead to the senescence of various types of cells that participate in atherosclerosis, including endothelial cells (ECs), endothelial progenitor cells (EPCs), vascular smooth muscle cells (VSMCs), macrophages, and adipose‐derived mesenchymal stem cells (AMSCs). These senescent cells display a decreased replication rate, increased inflammation and reactive oxygen species (ROS), which contribute to the development and progression of atherosclerosis. The senescent ECs impair the endothelium integrity and permeability, which facilitates the retention of oxidized LDL and eventually results in atherosclerosis. Senescent endothelial progenitor cells (EPCs) also present functional impairment of differentiation, migration and angiogenesis, leading to the dysfunction of vascularization and acceleration of atherosclerosis. In addition, the senescent VSMCs show poor proliferation capacity and calcific phenotypes, which means impaired function in forming the atherosclerotic fibrous cap and thus promotes plaque vulnerability. Moreover, senescent macrophages with increased lipid accumulation accelerate the formation of the atherosclerotic core. The senescent AMSCs in perivascular adipose tissue (PVAT) contribute to the pathogenesis of atherosclerosis through paracrine secretion of various inflammatory factors. Together, these senescent cells induced by dyslipidemia accelerate the occurrence and progression of atherosclerosis. FA, fatty acid; SA‐β‐gal, senescence‐associated β‐galactosidase.

Aging is an independent risk factor for the increased morbidity and mortality of atherosclerosis (North & Sinclair, 2012). At the cellular level, aging is caused by the accumulation of senescent cells. Cellular senescence is manifested by irreversible cell cycle arrest and distinctive phenotype alterations, including a flattened and enlarged morphology, increased activity of senescence‐associated β‐galactosidase (SA‐β‐gal) and upregulated senescence‐related proteins, such as p53, p21 and p16 (Lopez‐Otin *et al*., 2013). Importantly, one of the main characteristics of senescent cells is the production of senescence‐associated secretory phenotype (SASP) factors, including pro‐inflammatory cytokines, MMPs, and other factors (Gardner *et al*., 2015; Childs *et al*., 2016). Cellular senescence is likely a result of telomere shortening‐dependent replicative senescence (Silva *et al*., 2017) and/or stress‐induced premature senescence (SIPS) (Wang *et al*., 2018b) in response to a variety of endogenous and exogenous stimuli, such as DNA damage (Ungvari *et al*., 2013), oncogene signals (Quijano *et al*., 2012), mitochondrial dysfunction (Tyrrell *et al*., 2020) and some cardiovascular risk factors (Voghel *et al*., 2007). Intriguingly, cellular senescence sharing multiple causative stimuli with atherosclerosis, such as hyperlipidemia (Wang *et al*., 2018b), hypertension (Voghel *et al*., 2007), diabetes (Li *et al*., 2018b) and obesity (Burton & Faragher, 2018; Khoukaz *et al*., 2020), is an important driver for atherosclerosis. Emerging evidence indicates the existence of various types of senescent cells in atherosclerotic arteries, including ECs (Vasile *et al*., 2001; Minamino *et al*., 2002; Silva *et al*., 2017), VSMCs (Gorenne *et al*., 2006; Matthews *et al*., 2006; Wang *et al*., 2015), and macrophages (Childs *et al*., 2016). Importantly, senescence of other types of cells such as EPCs (Rauscher *et al*., 2003; Vemparala *et al*., 2013) and AMSCs (Parvizi *et al*., 2021) also contribute to atherosclerosis. These senescent cells all take part in the pathophysiological process of atherosclerosis (Fig. 1).

Among the various stimuli that cause cellular senescence during atherosclerosis, dyslipidemia seems to be the most common (Wang *et al*., 2018b). Dyslipidemia is characterized by a subset of lipid metabolism disorders involving abnormally elevated plasma levels or functional impairment of lipids or lipoproteins. There is much evidence that low‐density lipoprotein (LDL) and its modifications exert pro‐atherogenic effects through inducing senescence of vascular cells (Wang *et al*., 2018a,b; Ahmad & Leake, 2019), while elimination of these senescent cells stabilizes the fibrous cap (Childs *et al*., 2016). In addition, remnant‐like lipoproteins (RLPs) (Pu & Liu, 2007; Liu *et al*., 2009a; Yang *et al*., 2011) and fatty acids (FAs) (Li *et al*., 2018a; Wang *et al*., 2020; Grootaert *et al*., 2021) that are hydrolysed products from triglyceride‐rich lipoproteins (TRLs) accelerate cellular senescence in atherosclerosis. In addition, high‐density lipoprotein (HDL), usually regarded as an anti‐senescence lipoprotein, can result in cellular senescence when at high concentrations or dysfunctional (Huang *et al*., 2012; Park *et al*., 2016). These observations suggest that dyslipidemia may be a key contributory factor linking cellular senescence and atherosclerosis.

In this review, we first summarize existing evidence on the senescence of various types of cells induced by dyslipidemia in atherosclerosis, with a focus on LDL and its modifications, hydrolysate of TRLs, and HDL. We then discuss the mechanisms connecting dyslipidemia‐induced cellular senescence and atherosclerosis. Finally, we discuss potential therapeutic strategies for atherosclerosis by targeting dyslipidemia‐induced cellular senescence and highlight the future challenges and improvements that are still required.

## II. EVIDENCE AND MECHANISMS OF DYSLIPIDEMIA‐INDUCED EC SENESCENCE IN ATHEROSCLEROSIS

Table 1 provides an overview of the factors involved in dyslipidemia‐induced EC senescence.

**Table 1:** Factors involved in dyslipidemia‐induced endothelial cell (EC) senescence

Factors	Functions	Mechanisms
Oxidized LDL	Increased SA‐β‐gal activity	GSK‐3β/catenin/p53 (Liu *et al*., 2015)
	Increased mitochondrial dysfunction	AMPK/p53/p21 (Zhang *et al*., 2017, 2018)
	Impaired proliferation; increased ROS and inflammation Increased ROS; impaired cell viability	NF‐κB, PI3K/AKT/eNOS (Bian *et al*., 2020) mTOR (Yao *et al*., 2017; Jiang *et al*., 2020)
	Increased SA‐β‐gal activity; upregulated p21 and p16	SIRT1/LKB1/AMPK (Zhang *et al*., 2019)
	Decreased proliferation; upregulated p21	SIRT1/Beclin‐1 (Shi *et al*., 2020)
Electronegative LDL	Increased DNA damage and nuclear γH2AX deposition	ROS/ATM/CHK2/p53 (Wang *et al*., 2018b)
Native LDL	Impaired proliferation; increased ROS	p53 and p16 (Oh *et al*., 2017; Yoon, Chay & Yang, 2019)
Palmitate	Impaired proliferation; increased ROS	PKR/JNK/SIRT1 (Y. Li *et al*., 2018a)
Palmitate and HG	Increased ROS; mitochondrial dysfunction	AKT (Liao *et al*., 2017)
	Increased ROS; upregulated p21 and p16	AMPK‐mediated autophagy (Wang *et al*., 2020)
LA	Impaired proliferation	JNK‐mediated autophagy (Lee *et al*., 2015)
Mediterranean diet	Decreased ROS, apoptosis and telomere shortening	Epigenetic alterations (Marin *et al*., 2012; Yubero‐Serrano *et al*., 2020)
EPA/DHA	Decreased ROS, inflammation and thrombosis	Angiotensin improvement (Yamagata, Suzuki & Tagami, 2016; Qureshi *et al*., 2020)
	Decreased DNA damage and ROS	Nrf2 (Sakai *et al*., 2017)
HDL containing glycated apoA‐1	Increased lysosomal enlargement, impaired anti‐senescence and insulin‐secretion activities	Structural modification (Park *et al*., 2016)

AKT, protein kinase B; AMPK, adenosine monophosphate‐activated protein kinase; apoA‐1, apolipoprotein A‐1; ATM, ataxia‐telangiectasia mutated; CHK2, checkpoint kinase 2; DHA, docosahexaenoic acid; eNOS, endothelial nitric oxide synthase; EPA, eicosapentaenoic acid; GSK‐3β, glycogen synthase kinase 3 beta; γH2AX, phosphorylated histone protein H2AX; HDL, high‐density lipoprotein; HG, high glucose; JNK, c‐Jun N‐terminal kinase; LA, linoleic acid; LDL, low‐density lipoproteins; LKB1, liver kinase B1; mTOR, mammalian target of rapamycin; NF‐κB, nuclear factor kappa‐B; Nrf2, nuclear factor erythroid 2‐related factor 2; PI3K, phosphatidylinositol 3‐kinase; PKR, protein kinase R; ROS, reactive oxygen species; SA‐β‐gal, senescence‐associated β‐galactosidase; SIRT1, silent information regulator 1.

### (1) EC senescence in atherosclerosis

ECs are essential to maintain the structural integrity and homeostasis of blood vessels (Li, Sun & Carmeliet, 2019). The main biological functions of ECs in angiogenesis, coagulation, vasoconstriction and vasodilation have been fully elucidated (Gimbrone Jr. & Garcia‐Cardena, 2016). Pathogenic factors, such as advanced age, mechanical forces, excessive inflammation, or other disorders of the internal environment, can lead to EC dysfunction, eventually resulting in atherosclerosis (Donato *et al*., 2015; Bhayadia *et al*., 2016; Gimbrone Jr. & Garcia‐Cardena, 2016). A growing body of evidence indicates that senescent ECs are frequently found in atherosclerosis (Vasile *et al*., 2001; Minamino *et al*., 2002; Silva *et al*., 2017). The replicative senescence of ECs has been extensively investigated (Foreman & Tang, 2003; Cardus *et al*., 2013; Silva *et al*., 2017). It was reported that ECs at arterial bifurcation sites were more prone to show a senescence phenotype with shorter telomeres and increased inflammatory factors (Warboys *et al*., 2014). An earlier study from rats demonstrated that the proliferation potential of ECs declined with advanced age; the daily rate of EC replication declined from 13% at birth to 0.1–0.3% at an age of 5–6 months (Schwartz & Benditt, 1977). A decreased EC replication rate with advanced age may partly explain why elderly people are more prone to suffer from atherosclerotic diseases (Schwartz & Benditt, 1977). SIPS of ECs can be induced by most of the cardiovascular risk factors mentioned above (Voghel *et al*., 2007; Burton & Faragher, 2018; Wang *et al*., 2018b). A number of SASP factors are increased in senescent ECs, promoting the activation of monocytes and their infiltration into the subendothelial region (Stojanovic *et al*., 2020). In addition, senescent ECs compromise the endothelium integrity and permeability, facilitating the accumulation of oxidized LDL, which further contributes to atherosclerosis (Huynh *et al*., 2011; Peiffer, Sherwin & Weinberg, 2013; Steffensen *et al*., 2015; Boren *et al*., 2020). These findings support a close relationship between EC senescence and atherosclerosis.

### (2) LDL‐induced EC senescence

LDL tends to be oxidized and then promotes EC dysfunction as well as the formation, progression, and destabilization of atherosclerotic plaques (Akhmedov *et al*., 2014, 2017). Two scavenger receptors, lectin‐like oxidized low‐density lipoprotein receptor‐1 (LOX‐1) and cluster of differentiation 36 (CD36, also known as fatty acid translocase), mediate the entry and internalization of oxidized LDL in ECs (Akhmedov *et al*., 2014; Cho & Choi, 2019). Among the two receptors, LOX‐1 is the major receptor in ECs and is highly expressed in atherosclerotic lesions, while CD36 seems to be mainly expressed in microvascular rather than macrovascular ECs (Di Pietro, Formoso & Pandolfi, 2016). Increasing evidence showed that oxidized LDL promoted EC senescence, which resulted in atherosclerosis both *in vivo* and *in vitro* (Shi *et al*., 2007; Liu *et al*., 2015; Jiang *et al*., 2020). A number of studies found that oxidized LDL increased the production of intracellular reactive oxygen species (ROS) and induced mitochondrial dysfunction, which in turn promoted EC senescence (Liu *et al*., 2015; Yao *et al*., 2017; Zhang *et al*., 2017, 2018; Bian *et al*., 2020). The increased ROS in ECs induced by oxidized LDL were mainly dependent on NADPH oxidases (Liang *et al*., 2018), nuclear factor erythroid 2‐related factor 2 (Nrf2) (Huang *et al*., 2013), and myeloperoxidase (Liu *et al*., 2015). The downstream activation of the β‐catenin/p53 signalling pathway was also involved in this process (Liu *et al*., 2015). These findings demonstrated that oxidative stress is a crucial mechanism for oxidized LDL‐induced EC senescence.

Importantly, there is close cross‐talk between increased oxidative stress and activated inflammation (Csiszar *et al*., 2008). The increased production and wide range of inflammatory factors not only represent a phenotype of senescent cells, but also play a key role in promoting EC senescence and dysfunction (Donato *et al*., 2009; Hasegawa *et al*., 2012). The adhesion factors and pro‐inflammatory molecules intercellular cell adhesion molecule‐1 (ICAM‐1), vascular cell adhesion molecule‐1 (VCAM‐1), interleukin 6 (IL‐6), and IL‐8 are significantly upregulated in oxidized LDL‐induced senescent ECs and subsequently recruit a large number of circulating monocytes to the atherosclerotic lesions, which contribute to atherosclerosis (Bian *et al*., 2020; Jiang *et al*., 2020).

Additionally, excessive intracellular oxidative stress also triggers the autophagy pathway. Autophagy, a subcellular process for lysosome‐mediated degradation of cytoplasmic materials, is essential for maintaining cellular homeostasis and vascular function (De Meyer *et al*., 2015). It is clear that the activation of autophagy initially plays a protective role in EC function through removal of oxidative stimuli and the promotion of cell survival (Torisu *et al*., 2016). Recent studies reported that activation of autophagy by anti‐senescence agents protected ECs from oxidized LDL‐induced senescence (Zhang *et al*., 2019; Shi *et al*., 2020). However, the failure of autophagy often results in cellular senescence. Therefore, oxidized LDL not only can be internalized more easily by macrophages, but also can recruit circulating monocytes to the subendothelium by inducing EC senescence and releasing adhesion molecules, promoting the occurrence and development of atherosclerosis.

Recently, it has been found that non‐coding RNAs (ncRNAs), mainly microRNAs (miRNAs; miRs) and long non‐coding RNAs (lncRNAs), promote vascular cell senescence (Ni *et al*., 2020). The upregulated expression of miR‐21‐5p and miR‐203a‐3p was detected not only in the plasma of hyperlipidemic rats but also in oxidized LDL‐treated ECs, which accelerated EC senescence through downregulation of dynamin‐related protein 1 (Drp1) and the activation of downstream adenosine monophosphate‐activated protein kinase (AMPK)/p53/p21 signalling pathway (Zhang *et al*., 2017). On the contrary, inhibiting miR‐21‐5p/miR‐203a‐3p significantly restored the mitochondrial dysfunction and EC senescence (Zhang *et al*., 2018). This indicates that some miRNAs take part in modulating oxidized LDL‐induced EC senescence. In addition, lncRNA non‐coding RNA activated by DNA damage (NORAD)‐knockdown promoted oxidized LDL‐induced EC senescence, accompanied by aggravated inflammation and subsequent atherosclerosis (Bian *et al*., 2020). Since increased attention is now being given to ncRNAs, further studies are likely to elucidate their specific role in oxidized LDL‐induced EC senescence in atherosclerosis.

**Fig. 2:**
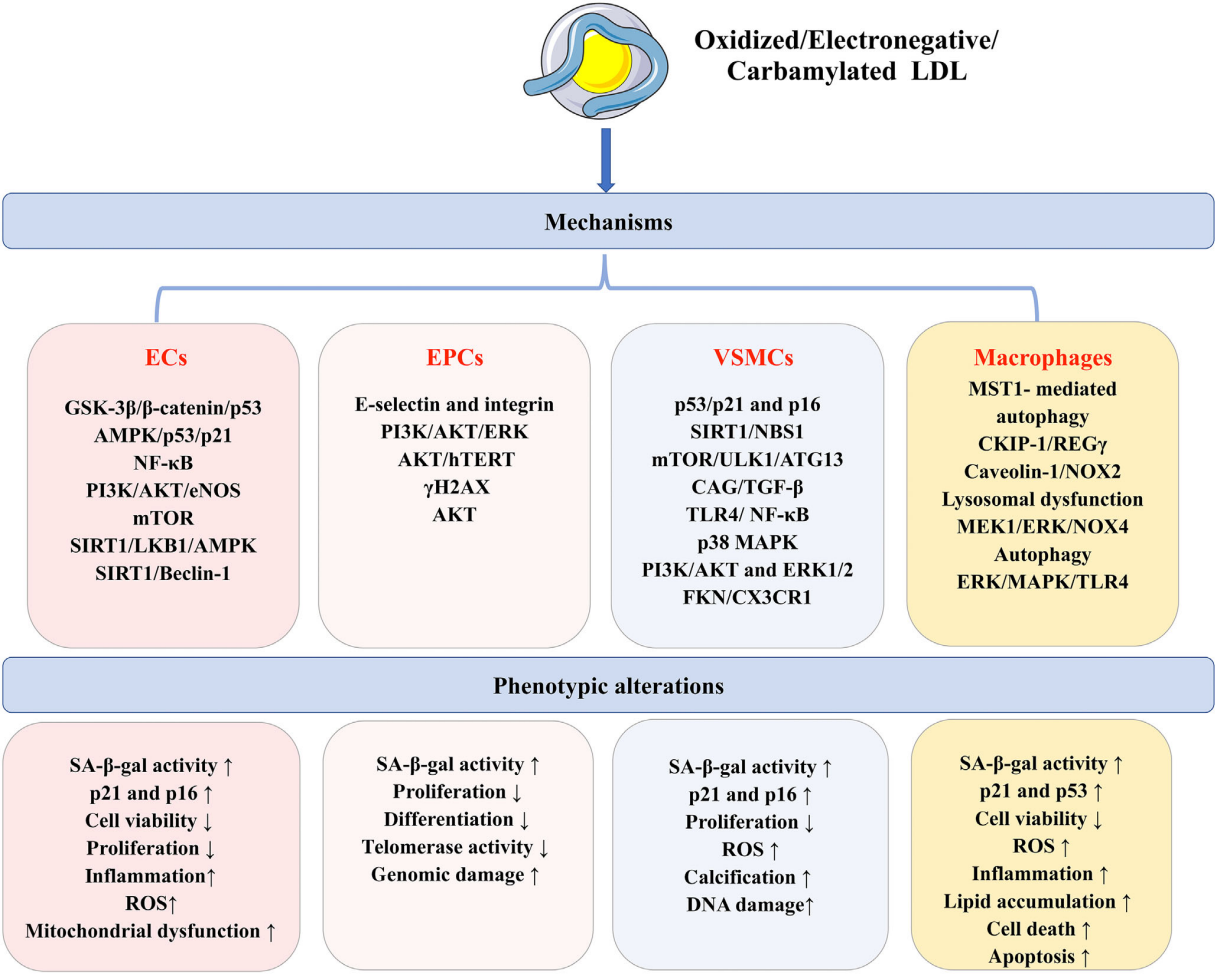
Cellular senescence of various types of cells induced by low‐density lipoprotein (LDL). LDL is a strong pro‐atherogenic lipoprotein, especially in its modified forms oxidized LDL, electronegative LDL and carbamylated LDL. They can lead to the senescence of endothelial cells (ECs), endothelial progenitor cells (EPCs), vascular smooth muscle cells (VSMCs) and macrophages *via* activation of multiple signalling pathways related to oxidative stress, inflammation and autophagy. The phenotypic profiles of these senescent cells are functionally impaired, resulting in atherosclerosis. AKT, protein kinase B; AMPK, adenosine monophosphate‐activated protein kinase; ATG13, autophagy‐related protein 13; CAG, glycosaminoglycan; CKIP‐1, casein kinase 2‐interacting protein‐1; CX3CR1, C‐X3‐C motif chemokine receptor 1; eNOS, endothelial nitric oxide synthase; ERK, extracellular regulated protein kinase; FKN, Fractalkine; GSK‐3β, glycogen synthase kinase 3 beta; γH2AX, phosphorylated histone protein H2AX; hTERT, human telomerase reverse transcriptase; LKB1, liver kinase B1; MAPK, mitogen‐activated protein kinase; MEK1, mitogen‐activated protein kinase 1; MST1, mammalian ste20‐like kinase 1; mTOR, mammalian target of rapamycin; NBS1, Nijmegen Breakage Syndrome‐1; NF‐κB, nuclear factor kappa‐B; NOX, NADPH oxidase; PI3K, phosphatidylinositol 3‐kinase; REGγ, 11S regulatory particles, 28 kDa proteasome activator, proteasome activator subunit 3; ROS, reactive oxygen species; SA‐β‐gal, senescence‐associated β‐galactosidase; SIRT1, silent information regulator 1; TGF‐β, transforming growth factor β; TLR4, toll‐like receptor 4; ULK1, Unc‐51 like autophagy activating kinase 1.

Electronegative LDL is a naturally occurring modified form of LDL with a higher content of triglyceride and FAs than non‐electronegative LDL and is thought to be as atherogenic as oxidized LDL (De Castellarnau *et al*., 2000). It has been demonstrated that electronegative LDL promotes inflammation (De Castellarnau *et al*., 2000), apoptosis (Lu *et al*., 2008) and cytotoxicity (Tai *et al*., 2006) in ECs, contributing to EC dysfunction and atherosclerosis. These pathological processes also may lead to cellular senescence in ECs. It was reported that a high‐fat diet led to elevated serum levels of electronegative LDL, accompanied by accelerated senescence in the aortic endothelium (Wang *et al*., 2018b). In addition, *in vitro* experiments confirmed that EC senescence was promoted after incubation with electronegative LDL. The underlying mechanism might be related to increased ROS production and DNA damage, which promoted the deposition of nuclear phosphorylated histone protein H2AX (γH2AX) and the downregulation of human telomerase reverse transcriptase (hTERT). These alterations resulted in EC senescence through the ROS/ataxia‐telangiectasia mutated (ATM)/checkpoint kinase 2 (CHK2)/p53 pathway (Wang *et al*., 2018b) (Fig. 2).

Unlike oxidized LDL and electronegative LDL, few studies have investigated the role of native LDL, an LDL particle without any modifications, on EC senescence (Oh *et al*., 2017; Yoon *et al*., 2019). Paradoxically, it was found that native LDL, rather than oxidized LDL, promoted EC senescence (Oh *et al*., 2017). The role of native LDL in promoting EC senescence may be related to its direct internalization by ECs. Further exploration of the underlying mechanisms indicated that excessive generation of ROS and the activation of the p53 and p16 signalling pathways may be involved (Oh *et al*., 2017; Yoon *et al*., 2019). However, these *in vitro* studies are not directly applicable to human pathophysiology due to differences in redox status *in vivo*.

### (3) EC senescence induced by TRLs and FAs

It has been known that hypertriglyceridemia, which is caused by elevated levels of circulating TRLs, including chylomicron (CM), very‐low density lipoprotein (VLDL) and their remnants, aggravates atherosclerosis and is related to cardiovascular diseases (Zhang *et al*., 2008; Arsenault *et al*., 2009). In the circulation, triglycerides in CM and VLDL are hydrolysed by lipoprotein lipase (LPL) and release FAs (Xiang *et al*., 2020b). Unlike the well‐characterized receptors of TRLs in macrophages, there are fewer studies in ECs. It has been demonstrated that a VLDL receptor is responsible for the uptake of VLDL, while LOX‐1 performs this function for RLPs in ECs (Pacheco *et al*., 2001; Shin *et al*., 2004). LDL receptor related protein‐1 (LRP1) and sortilin‐related receptor may be involved in the receptor‐mediated uptake of TRLs (Ting *et al*., 2007). Current evidence demonstrates that TRLs promote the initiation and progression of pathogenic atherosclerosis partly through aggravating EC dysfunction (Shin *et al*., 2004; Norata *et al*., 2006), VSMC proliferation (Lipskaia *et al*., 2003; Bermudez *et al*., 2008) and macrophage foam cell formation (Botham *et al*., 2007; Takahashi, 2017). Although there is no direct evidence clarifying the role of TRLs on EC senescence, a large number of studies have demonstrated that they increase ROS production (Shin *et al*., 2004; Wang *et al*., 2008), inflammation (Norata *et al*., 2006; Rajamani *et al*., 2019) and apoptosis (Kawasaki *et al*., 2000; Shin *et al*., 2004) in ECs, supporting a pro‐senescence role of TRLs on ECs indirectly.

It is noteworthy that diverse FAs containing TRLs have distinct effects on EC function. For example, TRLs isolated from the blood following a high saturated fatty acids (SFAs) content meal promoted inflammation and ROS production in ECs, while a meal containing high monounsaturated fatty acids (MUFAs) and polyunsaturated fatty acids (PUFAs), especially n‐3 PUFA eicosapentaenoic acid (EPA) and docosahexaenoic acid (DHA), exerted the opposite effects (Mishra, Chaudhary & Sethi, 2004; Wan *et al*., 2010; Latham Birt *et al*., 2016). However, n‐6 PUFA linoleic acid (LA), a major product of TRLs, could be oxidized into various oxidized fractions that promoted EC inflammation (Wang *et al*., 2009).

Palmitate, the most abundant SFA in humans, has been reported remarkably to promote EC senescence through activation of protein kinase R (PKR) and the downstream c‐Jun N‐terminal kinase (JNK), which resulted in decreased activity of silent information regulator 1 (SIRT1) (Li *et al*., 2018a). Consistently, Liao *et al*. (2017) reported that EC senescence was induced by palmitate combined with high glucose, leading to increased ROS production, mitochondrial dysfunction and apoptosis, which was associated with activation of the protein kinase B (AKT) pathway. The AMPK signalling pathway is also involved in palmitate and high glucose‐induced EC senescence (Wang *et al*., 2020). There is relatively limited evidence about the effects of n‐6 PUFA LA on EC senescence, although LA does induce EC senescence through inhibition of autophagy *via* the JNK pathway (Lee *et al*., 2015). These studies suggest that SFAs and n‐6 PUFA may exert a pro‐senescence effect on ECs.

**Fig. 3:**
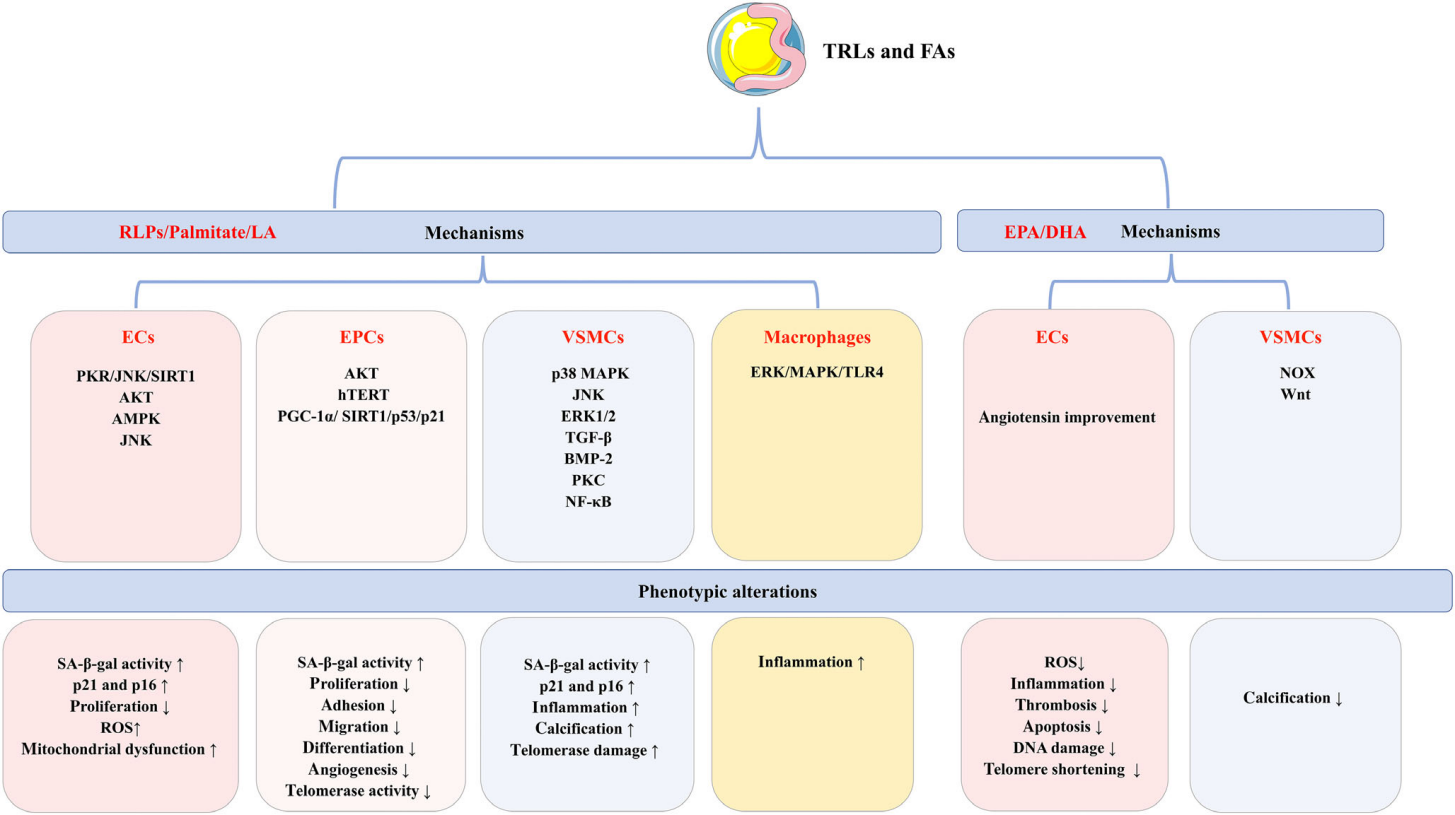
Cellular senescence of various types of cells induced by triglyceride‐rich lipoproteins (TRLs). TRLs and their hydrolysed products remnant‐like lipoproteins (RLPs) and fatty acids (FAs), influence various types of cellular senescence in atherosclerosis. RLPs, palmitate and linoleic acid (LA) promote senescence of these cells, while eicosapentaenoic acid (EPA) and docosahexaenoic acid (DHA) exert anti‐senescence properties in atherosclerosis. AKT, protein kinase B; AMPK, adenosine monophosphate‐activated protein kinase; BMP‐2, bone morphogenetic protein 2; ECs, endothelial cells; EPCs, endothelial progenitor cells; ERK1/2, extracellular regulated protein kinase 1/2; hTERT, human telomerase reverse transcriptase; JNK, c‐Jun N‐terminal kinase; MAPK, mitogen‐activated protein kinase; NF‐κB, nuclear factor kappa‐B; NOX, NADPH oxidase; PGC‐1α, peroxisome proliferator‐activated receptor coactivator1‐α; PKC, protein kinase C; PKR, protein kinase R; ROS, reactive oxygen species; SA‐β‐gal, senescence‐associated β‐galactosidase; SIRT1, silent information regulator 1; TGF‐β, transforming growth factor β; TLR4, toll‐like receptor 4; VSMCs, vascular smooth muscle cells; Wnt, wingless.

A randomized controlled trial from CORDIOPREV reported that a Mediterranean diet enriched in MUFAs restored EC dysfunction in patients with coronary artery disease (CAD), accompanied by decreased ROS production, cellular senescence and apoptosis in ECs (Yubero‐Serrano *et al*., 2020). The decreased ROS production accompanying a Mediterranean diet protected against telomere shortening in ECs, which prevented cellular senescence and apoptosis (Marin *et al*., 2012). There is increasing evidence for an anti‐senescence role of EPA and DHA on ECs. A study from rats demonstrated that ingestion of EPA and DHA attenuated the pro‐senescence, pro‐inflammatory and pro‐thrombotic effects of microvesicles from cultured rat spleen‐derived leukocytes on ECs (Qureshi *et al*., 2020). Another *in vitro* study supported these results, showing that either EPA or DHA attenuated hydrogen peroxide (H_2_O_2_)‐induced DNA damage, ROS production and cellular senescence in ECs through enhancing the Nrf2‐mediated antioxidant response (Sakai *et al*., 2017). However, the anti‐senescence effects of EPA and DHA on ECs were not observed after blocking VCAM‐1 expression, suggesting that the anti‐senescence role of EPA and DHA may be ascribed to anti‐inflammatory effects (Belcastro *et al*., 2021). In addition, DHA prevented tumour necrosis factor alpha (TNF‐α)‐induced EC dysfunction and senescence, supported by a decrease in SA‐β‐gal‐positive cells and expression of some genes related to pro‐thrombotic and pro‐inflammatory effects (Yamagata *et al*., 2016). Therefore, it is reasonable to speculate that the beneficial effects of a Mediterranean diet or n‐3 PUFA‐enriched diet on atherosclerosis may be partly due to their anti‐senescence properties on ECs (Fig. 3).

### (4) HDL and apolipoprotein A‐1 (apoA‐1)‐induced EC senescence

Although compelling evidence has demonstrated inverse associations between HDL cholesterol level and cardiovascular outcomes, therapeutic interventions that increase HDL cholesterol level such as cholesteryl ester transfer protein (CETP) inhibitors, have not been shown to reduce the risk of cardiovascular events (Barter *et al*., 2007; Lincoff *et al*., 2017). These observations led to the hypothesis that the atheroprotective role of HDL may be dependent on its function rather than on its cholesterol content (Rosenson *et al*., 2016). It has been demonstrated that impaired HDL function such as low cholesterol efflux capacity, low levels of apoA‐1 and sphingosine‐1‐phosphate in HDL, and the presence of pro‐inflammatory and pro‐oxidant particles in HDL, were associated with a higher risk of acute coronary syndrome (Soria‐Florido *et al*., 2020). Additionally, probucol has been shown to reduce the development of atherosclerosis in rabbits by improving HDL function, although it reduces HDL cholesterol level (Zhong *et al*., 2011). This evidence suggests that improving or maintaining the function of HDL may be more protective against atherosclerosis than elevating HDL cholesterol level.

The beneficial effects of HDL on ameliorating endothelial dysfunction through its anti‐oxidation, anti‐inflammation and anti‐apoptosis properties have been demonstrated (Rosenson *et al*., 2016). However, dysfunctional HDL caused by some cardiovascular risk factors, such as diabetes (Morgantini *et al*., 2011) and cigarette smoking (He, Zhao & Peng, 2013), or by post‐translation modifications of apoA‐1 including oxidation, nitration and glycation (Ferretti *et al*., 2006; Park *et al*., 2010), can impair or abolish its protective role on atherosclerosis, or even result in completely opposite effects. Advanced glycation end products are a key feature in diabetic patients with premature atherosclerosis (de la Cruz‐Ares *et al*., 2020), and are associated with increased mortality of CAD (Kilhovd *et al*., 2005). Intriguingly, it was found that HDL had an anti‐senescence effect on ECs, while reconstituted HDL containing the glycated apoA‐1 had the opposite effect (Park *et al*., 2016). Considering the close relationship of oxidation, inflammation and apoptosis with EC senescence as discussed above, it is plausible to conjecture that HDL may have an anti‐senescence role on ECs, while dysfunctional HDL may have the opposite role.

## III. EVIDENCE AND MECHANISMS OF DYSLIPIDEMIA‐INDUCED EPC SENESCENCE IN ATHEROSCLEROSIS

Table 2 provides an overview of the factors involved in dyslipidemia‐induced EPC senescence.

**Table 2:** Factors involved in dyslipidemia‐induced endothelial progenitor cell (EPC) senescence

Factors	Functions	Mechanisms
Oxidized LDL	Decreased tube formation and adhesion	Downregulated E‐selectin and integrin (Di Santo *et al*., 2008)
	Impaired telomerase activity and proliferation Impaired telomerase activity; increased ROS	PI3K/AKT/ERK (Ming *et al*., 2016) AKT/hTERT (Lai & Liu, 2015)
Carbamylated LDL	Increased genomic damage and impaired proliferation	Downregulated γH2AX (Carracedo *et al*., 2011)
Electronegative LDL	Impaired telomerase activity and differentiation	AKT (Tang *et al*., 2008)
RLPs	Decreased proliferation, adhesion, migration and telomerase activity	Inhibited AKT and hTERT (Liu *et al*., 2009a; Yang *et al*., 2011)
HG and FAs	Impaired angiogenesis	PGC‐1α/ SIRT1/p53/p21 (Song *et al*., 2017a)
Low HDL	Increased tube formation and anti‐senescence	Activated PI3K/AKT/eNOS (Huang *et al*., 2012)
High HDL	Decreased tube formation and pro‐senescence	Inhibited PI3K/AKT/eNOS (Huang *et al*., 2012)

AKT, protein kinase B; eNOS, endothelial nitric oxide synthase; ERK, extracellular regulated protein kinase; FAs, fatty acid; γH2AX, phosphorylated histone protein H2AX; HDL, high‐density lipoprotein; HG, high glucose; hTERT, human telomerase reverse transcriptase; LDL, low‐density lipoprotein; PGC‐1α, peroxisome proliferator‐activated receptor coactivator1‐α; PI3K, phosphatidylinositol 3‐kinase; RLPs, remnant‐like lipoprotein; ROS, reactive oxygen species; SIRT1, silent information regulator 1.

### (1) EPC senescence in atherosclerosis

EPCs are mononuclear cells derived from bone marrow and other tissues, and express some cell surface markers including CD34, vascular endothelial growth factor receptor‐2 (VEGF‐2, also named KDR) and CD133 (Fadini *et al*., 2008; Liu *et al*., 2009b). They are crucial for maintaining the dynamic equilibrium between EC loss and regeneration, playing a pivotal role in vascular repair (Zampetaki *et al*., 2008). Endothelium damage triggers the migration of EPCs to the injury sites where they differentiate into mature ECs and help to repair the vasculature. EPCs play a prominent role in atherosclerotic diseases; a reduced number and dysfunction of EPCs can be biomarkers and predictors for atherosclerosis (Schmidt‐Lucke *et al*., 2005; Keymel *et al*., 2008). It has been demonstrated that patients with CAD have a lower number and activity of circulating CD34^+^KDR^+^ EPCs, which manifests as an impaired migratory response and vascularization. Furthermore, the number and migratory ability of EPCs in CAD patients was inversely correlated with cardiovascular risk factors, such as hypertension, diabetes and LDL‐cholesterol level (Vasa *et al*., 2001). EPC senescence is accelerated in atherosclerosis (Vemparala *et al*., 2013) and can be induced by various atherosclerotic risk factors, such as hyperlipidemia (Imanishi *et al*., 2004; Tang *et al*., 2008; Liu *et al*., 2009a; Carracedo *et al*., 2011), hyperglycemia (Kuki *et al*., 2006) and advanced age (Thum *et al*., 2007). We have previously summarized the evidence for EPC senescence caused by various atherosclerotic risk factors, which was co‐modulated by p16 and telomerase (Yang, Liu & Zheng, 2008).

### (2) LDL‐induced EPC senescence

It is well recognized that oxidized LDL impairs EPC function, with the evidence indicating decreased capacity for tube formation, proliferation, migration, adhesion and nitric oxide (NO) generation (Ma *et al*., 2006; Wu *et al*., 2009; Ji *et al*., 2015; Qian *et al*., 2019; Shih *et al*., 2019). LOX‐1, instead of the scavenger receptor class B type 1 (SR‐B1) or scavenger receptor class A (SR‐A), was the main scavenger receptor responsible for the uptake of oxidized LDL (Shih *et al*., 2019). During this process, EPC dysfunction was related to increased inflammation and apoptosis together with impaired endothelial nitric oxide synthase (eNOS) expression, NO production and autophagy (Ma *et al*., 2006; Wu *et al*., 2009; Zhi *et al*., 2016; Qian *et al*., 2019). Recently, growing evidence has demonstrated that oxidized LDL accelerates EPC senescence, which contributes to EPC dysfunction (Imanishi *et al*., 2004; Lai & Liu, 2015; Lu *et al*., 2015; Ming *et al*., 2016). Di Santo *et al*. (2008) reported that oxidized LDL diminished the angiogenic potential of EPCs, which was associated with reduced levels of E‐selectin and integrin αvβ5. As mentioned above, telomerase played an important role in EPC senescence. In oxidized LDL‐induced EPC senescence, telomerase activity was found to be impaired, which may be associated with ROS‐dependent inhibition of AKT/hTERT phosphorylation (Lai & Liu, 2015; Lu *et al*., 2015). In addition, phosphatidylinositol 3‐kinase (PI3K)/AKT, a key signalling pathway that regulates cellular senescence, was involved in oxidized LDL‐induced EPC senescence (Lai & Liu, 2015; Ming *et al*., 2016).

There has been limited evidence about the role of electronegative LDL on EPCs. Among patients with cigarette smoking, EPCs are reduced in number and functional activities, which may partly explain why cigarette smoking clearly increased the risk of atherosclerosis (Michaud *et al*., 2006). However, the specific mechanisms by which smoking affects EPC function remain unclear. Recently, Tang *et al*. (2008) found that the content of electronegative LDL was higher in cigarette smokers than in non‐smoking subjects and significantly promoted EPC senescence and apoptosis, leading to impaired differentiation capacity and telomerase activity. This may shed light on the pro‐atherosclerotic mechanisms of cigarette smoking by focusing on the effects of specific modifications of LDL, such as electronegative LDL, on EPC senescence.

It has been demonstrated that carbamylated LDL level was increased in patients with chronic kidney disease (CKD), which accelerated atherosclerosis in CKD patients (Ok *et al*., 2005). Interestingly, Carracedo *et al*. (2011) reported that carbamylated LDL triggered genomic damage, increased oxidative stress and decreased the proliferation and angiogenesis capacities of EPCs, resulting in EPC senescence. The underlying mechanism may be related to the downregulation of γH2AX. Moreover, the injury to EPCs induced by carbamylated LDL was comparable to that of oxidized LDL, indicating that carbamylated LDL‐induced EPC senescence is likely to contribute to the increased mortality of atherosclerosis seen in CKD patients. The results suggest that specific modifications of LDL, such as carbamylation, can accelerate EPC senescence and contribute to atherosclerosis (Fig. 2).

### (3) EPC senescence induced by TRLs and FAs

As mentioned above, RLPs are hydrolysed from TRLs and are thought to be as atherogenic as LDL (Fujioka & Ishikawa, 2009). An elevated plasma level of RLP cholesterol is closely related to CAD and is an independent predictor for CAD risk especially in postprandial state (Imke *et al*., 2005; Xiang *et al*., 2020b), but the underlying mechanisms remain elusive. We have previously identified that RLPs increased the number of SA‐β‐gal positive EPCs, accompanied by a decreased capacity for proliferation, adhesion and migration, indicating that RLPs accelerated EPC senescence (Liu *et al*., 2009a). Further exploration revealed that RLPs resulted in increased ROS production, leading to inhibition of AKT and hTERT phosphorylation, thus suppressing telomerase activity and accelerating EPC senescence (Yang *et al*., 2011). These studies suggest that RLPs play a pivotal role linking EPC senescence and atherosclerosis.

Very few studies have focused on the role of FAs on EPC senescence. Song *et al*. (2017a) demonstrated that exposure of EPCs to a diabetes‐mimicking stimulus that contained high glucose and FAs promoted cellular senescence and compromised angiogenesis ability in EPCs. Meanwhile, the expression of peroxisome proliferator‐activated receptor coactivator1‐α (PGC‐1α) was upregulated, with the activation of the downstream SIRT1/p53/p21 pathway. Deletion of PGC‐1α attenuated the pro‐senescence phenotype in EPCs, indicating that PGC‐1α was a negative regulator of EPC senescence in diabetes. However, EPA and DHA were found to alleviate the tube formation and cell migration of EPCs under high‐glucose conditions, indicating a protective role of EPA and DHA on EPC senescence (Chiu *et al*., 2014, 2017). Future research should investigate the effects of different species of FAs on EPC senescence (Fig. 3).

### (4) HDL‐induced EPC senescence

It is known that HDL reinforces endothelial repair ability through increasing the number and functional activities of EPCs with elevated NO production and angiogenesis through the PI3K/AKT pathway (Tso *et al*., 2006; Noor *et al*., 2007; Sumi *et al*., 2007). Hence, we previously proposed that HDL could prevent or slow down EPC senescence by increasing NO production and enhancing PI3K/AKT‐mediated telomerase activity (Pu & Liu, 2008). Intriguingly, a novel role of HDL on EPC function was reported showing that HDL reversed oxidized LDL‐induced EPC viability loss and cytotoxicity in a dose‐dependent manner (Huang *et al*., 2012; Shih *et al*., 2019). However, when EPCs were incubated with HDL under oxidized LDL‐free conditions, the results seemed paradoxical. On the one hand, low concentrations of HDL (5–50 μg/ml) enhanced tube formation in EPCs through the activation of the PI3K/AKT/eNOS pathway. On the other hand, HDL at high concentrations (400–800 μg/ml) accelerated EPC senescence with decreased tube formation *via* activating the Rho‐associated kinase (ROCK) pathway but inhibiting the PI3K/AKT/eNOS pathway (Huang *et al*., 2012). The loss of a protective role of HDL at high concentrations may partly explain why elevation of HDL has failed to produce clinical benefits.

## IV. EVIDENCE AND MECHANISMS OF DYSLIPIDEMIA‐INDUCED VSMC SENESCENCE IN ATHEROSCLEROSIS

Table 3 provides an overview of the factors involved in dyslipidemia‐induced VSMC senescence.

**Table 3:** Factors involved in dyslipidemia‐induced vascular smooth muscle cell (VSMC) senescence

Factors	Functions	Mechanisms
Oxidized LDL	Increased SA‐β‐gal activity and ROS	p53/p21 and p16 (Zhu *et al*., 2019)
	DNA damage	SIRT1/NBS1 (Gorenne *et al*., 2013)
	Impaired proliferation; increased p16 and p21	mTOR/ULK1/ATG13 (Luo *et al*., 2017)
	Pro‐calcification	CAG/TGF‐β (Yan *et al*., 2011) TLR4/NF‐κB (Song *et al*., 2017b) p38 MAPK (Liao *et al*., 2013) PI3K/AKT and ERK1/2 (Tang *et al*., 2015) FKN/CX3CR1 (Yang *et al*., 2020)
Lp (a)	Pro‐calcification	Increased ALP activity (Sun *et al*., 2002)
Palmitate	Increased inflammation; telomerase damage	p38 MAPK, JNK and ERK1/2 (Grootaert *et al*., 2021)
Palmitate	Pro‐calcification	ROS‐dependent ERK1/2 (Brodeur *et al*., 2013) TGF‐β, BMP‐2, PKC, NF‐κB (Son *et al*., 2020)
EPA	Anti‐calcification	NOX (Nakamura *et al*., 2017)
		Wnt (Saito *et al*., 2017)

AKT, protein kinase B; ALP, alkaline phosphatase; ATG13, autophagy‐related protein 13; BMP‐2, bone morphogenetic protein 2; CAG, glycosaminoglycan; CX3CR1, C‐X3‐C motif chemokine receptor 1; EPA, eicosapentaenoic acid; ERK1/2, extracellular regulated protein kinase 1/2; FKN, Fractalkine; JNK, c‐Jun N‐terminal kinase; LDL, low‐density lipoprotein; Lp(a), lipoprotein(a); MAPK, mitogen‐activated protein kinase; mTOR, mammalian target of rapamycin; NBS1, Nijmegen Breakage Syndrome‐1; NF‐κB, nuclear factor kappa‐B; NOX, NADPH oxidase; PI3K, phosphatidylinositol 3‐kinase; PKC, protein kinase C; ROS, reactive oxygen species; SA‐β‐gal, senescence‐associated β‐galactosidase; SIRT1, silent information regulator 1; TGF‐β, transforming growth factor β; TLR4, toll‐like receptor 4; ULK1, Unc‐51 like autophagy activating kinase 1; Wnt, wingless.

### (1) VSMC senescence in atherosclerosis

VSMCs, residing in the vasculature *media*, are critical for maintaining vascular homeostasis through regulating vessel contraction and relaxation, and producing the extracellular matrix (ECM) (Basatemur *et al*., 2019). In healthy arteries, a phenotype of contractile VSMCs is often present, with expression of a variety of contractile proteins [including α‐smooth muscle actin (α‐SMA), smooth muscle 22 α (SM22α), and smooth muscle myosin heavy chain (SMMHC)] and secretion of ECM components including collagen, elastin and proteoglycan. When confronted with vascular injury, VSMCs convert to a high‐proliferation synthetic phenotype with decreased α‐SMA and SM22α, but increased ECM, osteopontin (OPN) and inflammatory cytokines, facilitating their migration to the subendothelium (Basatemur *et al*., 2019). VSMC proliferation is beneficial to a certain extent in atherosclerosis through the formation of the fibrous cap that stabilizes plaques. However, in some advanced atherosclerotic plaques, senescent VSMCs are present with poor proliferation, which promotes the pathogenesis of atherosclerosis and plaque vulnerability (Bennett *et al*., 1998). Bennett *et al*. (1998) were the first to describe that VSMCs from human atherosclerotic plaques undergo premature senescence. Subsequently, accumulating evidence indicated the presence of senescent VSMCs in atherosclerotic lesions, contributing to the progression of atherosclerosis and plaque destabilization (Minamino *et al*., 2003; Wang *et al*., 2015). It is widely accepted that senescent VSMCs possess an impaired capacity for plaque repair and fibrous cap stabilization, resulting in plaque instability. Further studies on the mechanisms involved revealed that senescent VSMCs promote plaque destabilization through the secretion of SASP mediators, exacerbating vascular inflammation and upregulating MMPs, which promote the degradation of elastic fibres and reduce fibrous cap thickness (Gardner *et al*., 2015; Grootaert *et al*., 2021). Thus, VSMC senescence plays a crucial role in atherosclerosis.

Recently, it has become clear that VSMCs are able to adopt a spectrum of phenotypes including calcific, macrophagic and adipogenic phenotypes during atherosclerosis. Overwhelming evidence demonstrated that senescent VSMCs acquire an ‘osteoblast‐like’ phenotype, manifested by increased expression of several osteoblastic markers including runt‐related transcription factor 2 (RUNX‐2), alkaline phosphatase (ALP), OPN, osteoprotegerin (OPG), and bone morphogenetic protein 2 (BMP‐2) (Nakano‐Kurimoto *et al*., 2009; Burton, Matsubara & Ikeda, 2010; Liu *et al*., 2013). The osteoblastic secretory phenotype of VSMCs shows increased susceptibility to plaque calcification, thus contributing to plaque vulnerability (Hofmann Bowman *et al*., 2011; Cai *et al*., 2016).

### (2) Oxidized LDL‐induced VSMC senescence and calcification

It has been shown that excessive intake of oxidized LDL by VSCMs *via* LOX‐1, CD36 and toll‐like receptors (TLRs) is closely linked to atherosclerosis (Kataoka *et al*., 2001; Kiyan *et al*., 2014). Since a number of studies have confirmed the role of VSMC senescence in atherosclerosis, the relationship between LDL or oxidized LDL and VSMCs has attracted much attention. Hutchinson‐Gilford progeria syndrome, a genetic disease usually accompanied by abnormal accumulation of progerin protein, accelerates atherogenesis (Hamczyk, del Campo & Andres, 2018a). Increased LDL retention and accelerated atherosclerosis were reported in a progerin‐induced atherosclerosis murine model with either ubiquitous or VSMC‐specific progerin overexpression, suggesting a close relationship between atherosclerosis and LDL‐induced VSMC senescence (Hamczyk *et al*., 2018b). A study using atherosclerosis‐prone apolipoprotein E (apoE)‐knockout mice indicated that a high‐cholesterol diet significantly induced the progression and vulnerability of atherosclerotic plaques (Zhu *et al*., 2019). Further *in vitro* experiments found that oxidized LDL promoted VSMC senescence, accompanied by increased mitochondrial ROS production and activation of downstream p53/p21 and p16 pathways. Clearance of ROS markedly reversed oxidized LDL‐induced VSMC senescence, suggesting that oxidative stress may play an important role in this process (Zhu *et al*., 2019). It also has been reported that DNA damage was increased in VSMCs derived from atherosclerotic vessels and in oxidized LDL‐incubated VSMCs, leading to the decreased expression of the anti‐senescence protein SIRT1 and a downstream repair protein Nijmegen breakage syndrome‐1 (NBS1), which resulted in reduced fibrous cap thickness and plaque vulnerability (Gorenne *et al*., 2013). Inhibition of SIRT1 in genetic apoE‐knockout mice aggravated oxidized LDL‐induced DNA damage and senescence in VSMCs (Gorenne *et al*., 2013). These studies support a crucial role of VSMC senescence in atherosclerosis, in which oxidative stress and DNA damage may be involved.

Recently, an interesting crosstalk between VSMC autophagy and senescence in atherosclerosis has been delineated (Grootaert *et al*., 2018). When VSMCs were challenged by toxic stressors, autophagy‐related pathways were activated as an adaptive response to help VSMCs overcome these deleterious insults and remove impaired cells (Martinet *et al*., 2008). Nonetheless, defective autophagy in VSMCs results both in decreased cell proliferation rate and also increased cell death, contributing to atherosclerosis progression (Osonoi *et al*., 2018). It has been shown that moderate activation of autophagy suppressed VSMC senescence, while defective autophagy accelerated their senescence (Salabei & Hill, 2013). Ding *et al*. (2013) reported that exposure of VSMCs to modest concentrations of oxidized LDL enhanced autophagy, whereas exposure to high concentrations impaired autophagy. However, activation of autophagy by rapamycin markedly inhibited oxidized LDL‐induced VSMC senescence through downregulation of mammalian target of rapamycin (mTOR) and upregulation of downstream Unc‐51‐like autophagy activating kinase 1 (ULK1) and autophagy‐related protein 13 (ATG13) (Luo *et al*., 2017). Accordingly, successful autophagy is crucial in protecting against oxidized LDL‐induced VSMC senescence in atherosclerosis.

Growing evidence indicates that oxidized LDL promotes VSMC calcification. Yan *et al*. (2011) reported that oxidized LDL increased ALP activity and accelerated mineralization in VSMCs through promoting decorin glycosaminoglycan (CAG) chain biosynthesis and triggering the transforming growth factor β (TGF‐β) signalling pathway. Song *et al*. (2017b) showed that the TLR4/nuclear factor kappa‐B (NF‐κB) signalling pathway played a critical role in oxidized LDL‐induced VSMC calcification. In addition, signalling pathways of p38 mitogen‐activated protein kinase (MAPK) (Liao *et al*., 2013), PI3K/AKT (Tang *et al*., 2015), extracellular regulated protein kinase 1/2 (ERK1/2) (Tang *et al*., 2015), and Fractalkine (FKN)/C‐X3‐C motif chemokine receptor 1 (CX3CR1) (Yang *et al*., 2020) were activated in oxidized LDL‐induced VSMC calcification, while autophagy was inhibited (Liao *et al*., 2018). Most interestingly, incubation of VSMCs with lipoprotein(a) [Lp(a)], which consists of apolipoprotein(a) and a LDL‐like particle, showed increased ALP activity and calcium accumulation (Sun *et al*., 2002). All these studies suggest that VSMC calcification, as a phenotype of VSMC senescence, can be induced by oxidized LDL or Lp(a), and hence promote atherosclerosis (Fig. 2).

### (3) VSMC senescence induced by TRLs and FAs

Unlike the well‐investigated roles of TRLs on ECs, there is little known about the effects of TRLs on VSMCs. It has been demonstrated that CM remnants, rather than CM itself, significantly increase monocyte chemotactic protein (MCP‐1) expression in VSMCs in a p38 MAPK‐dependent manner, suggesting a pro‐atherogenic role of CM remnants through pro‐inflammatory effects (Domoto *et al*., 2003). Another study revealed that in addition to increasing the expression of pro‐inflammatory cytokines, CM remnants also induced the expression of early growth response factor‐1 through the ERK/MAPK kinase pathway, which contributed to the atherosclerotic process (Takahashi *et al*., 2005). Nevertheless, a few studies have implicated TRLs and RLPs in the promotion of VSMC proliferation (Kawakami *et al*., 2003; Bermudez *et al*., 2008), which may be associated with protein kinase C (PKC)‐dependent activation of epidermal growth factor receptor (Kawakami *et al*., 2003). Unfortunately, there is no direct evidence about the role of TRLs on VSMC senescence. On the one hand, TRLs promote inflammation in VSMCs, which seems to accelerate cellular senescence. On the other hand, TRLs promote VSMC proliferation, leading to the opposite phenotype of senescence. Hence, it remains unclear how TRLs influence VSMC senescence and further investigations are needed.

There is limited evidence regarding the role of FAs on VSMC senescence. It has been reported that VSMC senescence was accelerated in human atherosclerotic plaques or by palmitate incubation, accompanied by a decrease in the anti‐senescence protein SIRT6 (Grootaert *et al*., 2021). The p38 and JNK pathways were activated in this process, while ERK1/2 was inhibited (Grootaert *et al*., 2021). Overexpression of SIRT6 in VSMCs alleviated cellular senescence and inflammation both *in vivo* and *in vitro*, and inhibited the development of atherosclerosis *in vivo* (Grootaert *et al*., 2021). A few studies have focused on the role of palmitate on VSMC calcification (Brodeur *et al*., 2013; Son *et al*., 2020). Brodeur *et al*. (2013) demonstrated that palmitate promoted VSMC calcification through increased ROS production and subsequently led to the phosphorylation of ERK1/2. The TGF‐β, BMP‐2, PKC, and NF‐κB signalling pathways were also involved in palmitate‐induced VSMC calcification (Son *et al*., 2020). However, VSMC calcification induced by palmitate could be prevented by EPA, which was associated with inhibition of NF‐κB and long‐chain acyl‐CoA synthetase 3 (ACSL3) (Kageyama *et al*., 2013). Moreover, EPA decreased NADPH oxidase (NOX) expression and inhibited the wingless (Wnt) signalling pathway in a genetic vascular calcification mouse model, which resulted in the alleviation of VSMC and vascular calcification (Nakamura *et al*., 2017; Saito *et al*., 2017). Adipose triglyceride lipase (ATGL) is a major lipase that initiates triglyceride mobilization in both adipocytes and non‐adipocyte cells (Smirnova *et al*., 2006). It has been reported that VSMCs from ATGL‐deficient mice showed elevated triglyceride content and aggravated cellular senescence (Lin *et al*., 2013). However, it was uncertain whether increased triglyceride or its hydrolysates caused the senescence phenotype of VSMCs from ATGL‐mice. Based on these studies, it appears that saturated FAs accelerate VSMC senescence while n‐3 PUFA, especially EPA exert the opposite effects (Fig. 3).

## V. EVIDENCE AND MECHANISMS OF DYSLIPIDEMIA‐INDUCED MACROPHAGE SENESCENCE IN ATHEROSCLEROSIS

Table 4 provides an overview of the factors involved in dyslipidemia‐induced macrophage senescence.

**Table 4:** Factors involved in dyslipidemia‐induced macrophage senescence

Factors	Functions	Mechanisms
Oxidized LDL	Reduced cell viability, increased lipid accumulation and upregulated p53 and p21	MST1‐ mediated autophagy (Cao *et al*., 2019) CKIP‐1/REGγ (Jia *et al*., 2019; Li *et al*., 2018)
	Increased ROS Increased ROS Increased ROS and cell death Increased ROS and apoptosis	Caveolin‐1/NOX2 (Wang *et al*., 2018a) Lysosomal dysfunction (Ahmad & Leake, 2019) MEK1/ERK/NOX4 (Lee *et al*., 2010) Inhibited autophagy (Liu *et al*., 2016)
	Increased inflammation	ERK/MAPK/TLR4 (Youk *et al*., 2021)
Palmitate	Increased inflammation	ERK/MAPK/TLR4 (Youk *et al*., 2021)

CKIP‐1, casein kinase 2‐interacting protein‐1; ERK, extracellular regulated protein kinase; LDL, low‐density lipoprotein; MAPK, mitogen‐activated protein kinase; MEK1, mitogen‐activated protein kinase 1; MST1, mammalian ste20‐like kinase 1; NOX, NADPH oxidase; REGγ, 11S regulatory particles, 28 kDa proteasome activator, proteasome activator subunit 3; ROS, reactive oxygen species; TLR4, toll‐like receptor 4.

### (1) Potential relationship between macrophage‐induced inflammation and cellular senescence

It is accepted that dyslipidemia and chronic inflammation caused by the immune response are the two major mechanisms involved in atherosclerosis (Weber & Noels, 2011). The interaction between lipids and immune cells is a main cause of chronic inflammation in the arterial wall during atherogenesis. The retention of oxidized LDL in the vascular *intima* triggers the secretion of monocyte recruitment factors by ECs, which drives the infiltration and activation of immune cells. The activated monocytes mediate the internalization of oxidized LDL *via* several scavenger receptors such as LOX‐1, SR‐A, SR‐B1, CD36 and TLRs (Di Pietro *et al*., 2016; Akhmedov *et al*., 2021) and release a variety of inflammatory cytokines, leading to the formation of macrophage‐like foam cells (Bekkering *et al*., 2014). Recently, anti‐inflammation strategies have shown significant cardiovascular benefits in the Canakinumab Anti‐inflammation Thrombosis Outcomes Study (CANTOS) (Ridker *et al*., 2017). However, a similar attempt by the Cardiovascular Inflammation Reduction Trial (CIRT) reported negative results, but encouraged further exploration of the mechanistic diversity of inflammatory pathways in atherosclerosis (Ridker *et al*., 2019). These trials provided promising therapeutic anti‐inflammation targets for the treatment of atherosclerosis, yet clarification of the detailed mechanisms remains limited. Hence, strategies targeting upstream effectors of inflammation may be a potential solution. It has been proposed that cellular senescence is a critical upstream source of inflammatory factors and a strong pathogenetic cause for atherosclerosis (Stojanovic *et al*., 2020). Although inflammatory factors can be derived from various types of cells in atherosclerosis, immune cells are the most significant.

### (2) Oxidized LDL‐induced macrophage senescence

A recent study identified that senescent macrophages are present in atherosclerotic lesions and play a critical role at all stages of atherosclerosis (Childs *et al*., 2016). Additionally, a number of studies have demonstrated that oxidized LDL induces macrophage senescence, manifested by increased activity of SA‐β‐gal, impaired cell survival, enhanced lipid accumulation, aggravated inflammation, and upregulated expression of senescence‐related proteins such as p53, p21 and p16 (Li *et al*., 2018; Wang *et al*., 2018a; Ahmad & Leake, 2019; Jia *et al*., 2019). It has been reported that excessive LDL internalized by macrophages could be oxidized in lysosomes, leading to an altered lysosomal pH, which in turn induces macrophage senescence as well as increased ROS production and secretion of inflammatory cytokines. Inhibition of LDL oxidation in lysosomes significantly restored cellular senescence in macrophages (Ahmad & Leake, 2019). Wang *et al*. (2018a) reported that incubation of macrophages with oxidized LDL increased caveolin‐1 expression and the latter upregulated the level of the NADPH oxidase NOX2, which promoted cellular senescence. Lee *et al*. (2010) found that the enhanced production of intracellular ROS induced by oxidized LDL promoted cell death in macrophages, which contributed to the formation of necrotic cores and the progression of atherosclerotic plaques. Another NADPH oxidase, NOX4, was confirmed to be the key source of ROS (Lee *et al*., 2010). These results suggest that oxidative stress plays a vital role in oxidized LDL‐induced macrophage senescence.

Autophagy and apoptosis were also found to be involved in oxidized LDL‐induced macrophage senescence (Muller *et al*., 2001; Liu *et al*., 2016; Cao *et al*., 2019; Zhou *et al*., 2020). Oxidized LDL obviously induced apoptosis in macrophages, as evidenced by an increased number of terminal deoxynucleotidyl transferase‐mediated dUTP‐biotin nick end labelling (TUNEL)‐positive macrophages and upregulated expression of the apoptosis‐related proteins B‐cell lymphoma‐2 (Bcl‐2) associated X protein (Bax), Bcl‐2 homologous antagonist/killer (Bak), caspase‐9 and caspase‐3. At the same time, autophagy was significantly inhibited, as manifested by decreased autophagosome formation and downregulated expression of the autophagy markers Beclin1 and light chain 3 (LC3) (Zhang *et al*., 2016a). These results were further supported by Cao *et al*. (2019), who reported that impaired autophagy induced by oxidized LDL resulted in macrophage senescence. They also found that quercetin reduced foam cell formation and delayed cellular senescence partly through mammalian ste20‐like kinase 1 (MST1)‐mediated autophagy activation. Similarly, Liu *et al*. (2016) demonstrated that suppressed autophagy induced by oxidized LDL increased lipid accumulation and ROS production in macrophages, which aggravated mitochondrial dysfunction and apoptosis. These results imply that autophagy and apoptosis play an important role in macrophage senescence induced by oxidized LDL (Fig. 2).

### (3) Macrophage senescence induced by TRLs and FAs

It has been suggested that TRL uptake by monocytes leads to lipid accumulation, which in turn augments foam cell formation and inflammation. In addition to the extensively studied scavenger receptors SR‐A, SR‐B1 and CD36, other types of receptors such as the apolipoprotein B48 receptor, VLDL receptor, LDL receptor and LRP1 also mediate the internalization of TRLs in macrophages (Botham & Wheeler‐Jones, 2013). Although there is no direct evidence regarding the role of TRLs on macrophage senescence, numerous studies have revealed pro‐inflammatory and pro‐oxidant effects on macrophages (Stollenwerk *et al*., 2005a,b; Persson, Nilsson & Lindholm, 2006). Hence, it is likely that TRLs may promote macrophage senescence. Only a few studies have investigated the role of specific types of FAs carried in TRLs on macrophages (Youk *et al*., 2021), as summarized by Botham & Wheeler‐Jones (2013) in a recent review. Briefly, TRLs enriched in n‐3 PUFA suppressed the secretion of inflammatory cytokines by macrophages to a greater extent than those from a control diet or a diet containing more SFAs (De Pascale *et al*., 2009; Jinno *et al*., 2011). There is no evidence regarding the effects of these specific FAs on macrophage senescence, which deserves further exploration (Fig. 3).

## VI. EVIDENCE AND MECHANISMS OF DYSLIPIDEMIA‐INDUCED CELLULAR SENESCENCE OF ADIPOSE TISSUE IN ATHEROSCLEROSIS

### (1) Senescent adipose tissue in non‐elderly obese individuals

Adipose tissue represents the largest energy reservoir in the body, and is classified as subcutaneous white adipose tissue (sWAT) or visceral white adipose tissue (vWAT) according to anatomical site. In addition to its main function of storing energy, there is growing recognition that WAT is a major endocrine organ that produces a variety of bioactive factors essential for the maintenance of systemic metabolic homeostasis (Liu *et al*., 2020). PVAT is a type of vWAT that surrounds the blood vessels. Recently, PVAT has been regarded as an active constituent of the blood vessel walls involved in regulating vascular homeostasis through the endocrine and paracrine effects of a wide range of bioactive factors (Chatterjee *et al*., 2009; Qi *et al*., 2018). Dramatic changes in WAT occur with advanced age, manifested by a decline in sWAT quantity and redistribution of WAT from sWAT to the visceral depots. These changes lead to impaired fat‐storage capacity in sWAT and increased release of free FAs into circulation, which then overflow into visceral fat and organs, contributing to increased susceptibility to metabolic disorders (Goodpaster *et al*., 2003; Tchkonia *et al*., 2010).

However, increasing evidence has demonstrated that senescent cells not only accumulate in WAT of elderly individuals, but also in that of young obese mice and humans (Minamino *et al*., 2009; Roldan *et al*., 2011; Gustafson, Nerstedt & Smith, 2019). Obesity is a major contributing factor for cellular senescence in WAT, which links metabolic disorders and the aging process (Minamino *et al*., 2009; Burton & Faragher, 2018; Gustafson *et al*., 2019). Excess caloric intake is often accompanied by obesity, which results in adipose tissue dysfunction and senescence, and leads to an increased risk of metabolic disorders (Minamino *et al*., 2009). AMSCs are multipotent preadipocytes residing in the WAT, which can proliferate and differentiate into mature adipocytes to maintain the normal functions of WAT. The accumulation of senescent AMSCs in sWAT is a leading cause of the senescence and dysfunction of fat tissue, while clearance of these senescent AMSCs not only protects against age‐associated disorders but also increases the healthy lifespan (Baker *et al*., 2011; Palmer *et al*., 2019). Senescent AMSCs and WAT display impaired capacities in lipid metabolism, insulin resistance and aberrant secretion of adipocytokines (Liu *et al*., 2020). It can also interfere with the normal function of distal tissues through SASP, participating in atherogenesis (Pan *et al*., 2019; Schutz *et al*., 2019; Parvizi *et al*., 2021).

### (2) Effects of preadipocyte senescence on vascular cells

The amounts and functions of sWAT deteriorate with advanced age, related to the reduced proliferation and differentiation capacities of preadipocytes (Li *et al*., 2021). A decline in adipogenesis in sWAT in obese humans has also been associated with senescent preadipocytes (Gustafson *et al*., 2019). It is recognized that preadipocytes in sWAT possess a higher replication potential than those in vWAT, and are thus more vulnerable to cellular senescence (Van Harmelen, Rohrig & Hauner, 2004; Tchkonia *et al*., 2005). A recent study using single‐cell RNA sequencing found that the adipose precursor population was drastically decreased in aging mice (Nguyen *et al*., 2021). Importantly, they detected a subpopulation of aging‐dependent cells that emerged and accumulated in sWAT with impaired differentiation capacity and enhanced expression of pro‐inflammatory factors, which they named ‘aging‐dependent regulatory cells (ARCs)’. These findings confirm the crucial role of preadipocyte senescence on WAT dysfunction.

Another recent study demonstrated that senescent AMSCs in WAT influenced the function and phenotypes of the main types of vascular cells (Parvizi *et al*., 2021). In this study, a culture medium collected from senescent AMSCs in sWAT was used to incubate ECs, VSMCs, macrophages and AMSCs. The results showed an inflammatory phenotype, declined cell viability, impaired proliferation and migration, and deteriorated metabolic function in these cells. These maladaptive changes support the hypothesis that senescent AMSCs may contribute to the development of vascular diseases such as atherosclerosis through paracrine effects on the cellular components in blood vessels.

### (3) TRL‐induced AMSC senescence

As mentioned above, obesity is a crucial factor for WAT and AMSC senescence (Minamino *et al*., 2009; Burton & Faragher, 2018; Gustafson *et al*., 2019). Notably, diet‐induced obesity is often accompanied by postprandial hypertriglyceridemia, leading to an increased number of circulating TRLs (Tian *et al*., 2019). We have previously demonstrated that postprandial TRL in subjects with a high‐calorie diet induced AMSC senescence with increased inflammation and intercellular ROS (Xiang *et al*., 2020a). During this process, SIRT1 was found to be significantly decreased, which resulted in reduced deacetylation of p53 and subsequent increased expression of acetylated p53 and downstream p21. In addition, SASP factors such as IL‐1α, IL‐6 and MCP‐1 were upregulated in TRL‐incubated AMSCs. These inflammatory cytokines released by AMSCs are known to be involved in the development and progression of atherosclerosis (Wassmann *et al*., 2004; Chava *et al*., 2009; Abe *et al*., 2010; Ohman *et al*., 2010; Ghanbari *et al*., 2021). Hence, we propose that AMSC senescence induced by TRLs may contribute to atherogenesis through increased SASP components.

## VII. SENESCENCE‐RELATED THERAPEUTIC STRATEGIES FOR ATHEROSCLEROSIS

### (1) Anti‐atherosclerotic effects of promising geroprotectors

A key initial finding on slowing aging was the observation that caloric restriction (CR) in rodents had beneficial effects on the lifespan (see McCay *et al*., 1975). Since then, accumulating evidence has suggested that CR not only increases the lifespan but also protects against age‐associated disorders in several species (Mattison *et al*., 2017; Pifferi *et al*., 2018). There have been increasing efforts to understand the underlying mechanisms by which CR affects the aging process (Campisi *et al*., 2019). Since CR is related to the reduction of nutrients, some promising pharmaceutical strategies for delaying aging have focused on lowering the activity of the nutrition‐sensing network. A recent detailed review summarized a list of promising agents that are closest to clinical testing (Partridge, Fuentealba & Kennedy, 2020). Here, we highlight the anti‐atherosclerotic effects of some of these potential geroprotectors to shed a light on therapeutic strategies for atherosclerosis.

Senolytics selectively target and kill senescent cells by inducing apoptosis through regulating multiple apoptosis signalling pathways (Martel *et al*., 2020). Clearance of senescent cells by senolytics has showed great potential for delaying aging and aging‐related disorders in animal models (Xu *et al*., 2018). Quercetin, a plant flavonoid which is mainly effective against senescent ECs through inhibition of the Bcl‐2 family, has been demonstrated to attenuate atherosclerotic lesions *via* modulating oxidized LDL‐induced EC senescence (Jiang *et al*., 2020). It also showed anti‐senescence effects on oxidized LDL‐incubated macrophages and VSMCs (Li *et al*., 2018; Cao *et al*., 2019; Kim *et al*., 2020). However, quercetin is less effective on senescent preadipocytes. Hence, a combination of quercetin plus dasatinib, a senolytic which selectively remove senescent preadipocytes through inhibiting multiple tyrosine kinases, has become a promising strategy (Xu *et al*., 2018; Palmer *et al*., 2019). Intermittent administration of quercetin plus dasatinib has been shown to alleviate vasomotor dysfunction in aged and atherosclerotic mice (Roos *et al*., 2016). In addition to these two senolytics, fisetin has also been shown to have atheroprotective effects, manifested by reduced atherosclerotic plaque and lipid accumulation (Yan *et al*., 2021). The anti‐atherosclerotic role of fisetin may be related to the amelioration of oxidized LDL‐induced EC death, inflammation and dysfunction (Patel *et al*., 2019). It has also been demonstrated that fisetin protected macrophages from oxidized LDL‐induced senescence and lipid accumulation (Jia *et al*., 2019). However, although senolytics have shown promising results, there remain some issues, such as disturbance of non‐senescent cells, uncertainty as to the best time of use, and potential damage caused by the unremoved products of killed senescent cells (Partridge *et al*., 2020). Future targeting of senolytics to specific types of senescent cells may help to overcome these issues.

NAD^+^ is a critical redox coenzyme and supplementation of NAD^+^ has been shown to prolong lifespan and mitigate age‐related physiological decline in animals (Mills *et al*., 2016; Zhang *et al*., 2016b). Since NAD^+^ cannot be taken up directly by cells, current strategies involve administration of its precursors, among which nicotinamide riboside (NR) and nicotinamide mononucleotide (NMN) have been investigated (Yoshino, Baur & Imai, 2018). NMN supplementation dramatically increased NAD^+^ level, accompanied by improvement of age‐related arterial dysfunction and attenuation of oxidative stress in mice (de Picciotto *et al*., 2016). Further research revealed that the protective role of NMN on vascular function was related to anti‐aging changes in the expression profile of miRNAs (Kiss *et al*., 2019). Although NR can increase NAD^+^ levels and was well tolerated in humans, no geroprotective effects have been identified in humans so far.

Sirtuins are NAD^+^‐dependent deacylases and are implicated in a wide range of physiological processes, one of which is a protective role in age‐related diseases, thus increasing the healthspan and lifespan (Kane & Sinclair, 2018). There are seven mammalian sirtuins, with SIRT1 (Gorenne *et al*., 2013) and SIRT6 (Grootaert *et al*., 2021) investigated with regard to protection against atherosclerosis. Expression of SIRT1 and SIRT6 is decreased in atherosclerotic plaques, while overexpression of SIRT1 or SIRT6 significantly attenuated oxidized LDL‐ or palmitate‐induced VSMC senescence and inhibited atherosclerosis (Gorenne *et al*., 2013; Grootaert *et al*., 2021). In addition, SIRT1 and SIRT6 also alleviated senescence in ECs (Zu *et al*., 2010; Cardus *et al*., 2013) and EPCs (Ming *et al*., 2016), suggesting a protective role in modulating atherosclerosis. Based on such evidence, sirtuin‐activating compounds (STACs), such as resveratrol, SRT1720 and SRT2104 have been investigated in some clinical trials (Dai *et al*., 2018). Although contradictory results have been reported (Bonkowski & Sinclair, 2016), these advances suggest a potential clinical application for STACs and human clinical trials are currently in progress (Kane & Sinclair, 2018).

Metformin is an oral biguanide drug widely prescribed as a first‐line therapy for type 2 diabetes (Marshall, 2017). It has been well established that metformin regulates a variety of metabolic processes including, but not limited to, lipid‐lowering and cardiovascular benefits (Kooy *et al*., 2009). It also plays a role in mitigating aging processes and increased lifespan in *Caenorhabditis elegans* (Onken & Driscoll, 2010). Consequently, it is not surprising that metformin showed pleiotropic effects on ameliorating atherosclerosis and vascular senescence (Forouzandeh *et al*., 2014). Mechanistically, the anti‐senescence role of metformin on ECs (Arunachalam *et al*., 2014; Zhang *et al*., 2015; Karnewar *et al*., 2018) has been widely studied. Further investigations of the effects of this drug on the senescence of other types of cells are urgently required.

mTOR inhibitors, especially rapamycin, have been demonstrated to extend not only lifespan but also healthspan in animal models (Partridge *et al*., 2020). However, their roles in atherosclerosis are complex. On the one hand, some research supports an anti‐atherosclerosis role, related to inhibition of the inflammation response (Ai *et al*., 2014), activation of autophagy (Wang *et al*., 2013) and promotion of cholesterol efflux (Ma *et al*., 2007). On the other hand, dyslipidemia including increased total cholesterol, LDL cholesterol and triglyceride, often occurred when utilizing mTOR inhibitors; this is a main risk factor of atherosclerosis (Kasiske *et al*., 2008; Kurdi, Martinet & De Meyer, 2018). Nevertheless, mTOR inhibitors ameliorated dyslipidemia‐induced senescence of ECs (Zhou *et al*., 2016) and VSMCs (Luo *et al*., 2017), which was protective in atherosclerosis. Taken together, these results suggest that mTOR inhibitors have unique anti‐atherosclerotic roles through multiple mechanisms. Although there is a high incidence of the side effect of dyslipidemia, the anti‐atherosclerotic role of mTOR inhibitors may be associated with an anti‐senescence effect, which seems to be independent of changes in lipids.

### (2) Anti‐senescence effects of current lipid‐lowering drugs

Currently available lipid‐lowering drugs, especially statins (Gong *et al*., 2014), represent the main medications for the primary and secondary prevention of CAD due to their role in cholesterol reduction (Istvan & Deisenhofer, 2001; Cannon *et al*., 2004). However, results from some clinical trials demonstrated that the benefits of statins were higher than expected, and were independent of lipid‐lowering effects (Sever *et al*., 2003; Glynn *et al*., 2009; Oesterle, Laufs & Liao, 2017). These lipid‐lowering‐independent pleotropic effects included, among others, anti‐inflammation, anti‐oxidation, anti‐platelet‐activation and improvement of EC function (Oesterle *et al*., 2017). It has been demonstrated that statins inhibited senescence of ECs (Gong *et al*., 2014; Zhang *et al*., 2018), VSMCs (Mahmoudi *et al*., 2008; Nakano‐Kurimoto *et al*., 2009) and EPCs (Assmus *et al*., 2003). The underlying mechanisms were involved in the reduction of intracellular ROS (Gong *et al*., 2014), inhibition of the inflammatory response (Dichtl *et al*., 2003), restoration of mitochondrial dysfunction (Zhang *et al*., 2018), increased SIRT1 and eNOS expression (Ota *et al*., 2010), attenuation of DNA damage and telomere shortening (Mahmoudi *et al*., 2008), and regulation of cell cycle proteins (Assmus *et al*., 2003). This evidence implies that the protective role of statins on atherosclerosis may partly relate to inhibition of cellular senescence.

## VIII. CONCLUSIONS

(1) Accumulating evidence suggests that dyslipidemia, especially elevated LDL and its modified products, elevated TRL and its hydrolysate, and dysfunctional HDL is involved in the occurrence and development of atherosclerosis. The contributory effects of cellular senescence to atherosclerosis has received much interest due to common causative stimuli and consequences.

(2) Dyslipidemia‐induced EC senescence impairs the endothelium barrier and facilitates lipid deposition, resulting in EC dysfunction and atherosclerosis. The potential mechanisms are involved not only in oxidative stress, but also in inflammation, autophagy and some novel ncRNAs *via* several classical signalling pathways. Nevertheless, n‐3 PUFA and functional HDL may provide new insights into atherosclerosis due to their anti‐senescence effects on ECs.

(3) EPC senescence induced by various types of lipoproteins and FAs contributes to the pathogenic mechanisms of atherosclerosis. Since a relatively small number of studies have focused on specific FAs and HDL other than oxidized LDL, further in‐depth research is needed to clarify the underlying molecular mechanisms.

(4) Dyslipidemia‐induced VSMC senescence or calcification leads to VSMC dysfunction and fibrous cap thinning, which contributes to atherosclerotic plaque vulnerability. The effects of different types of FAs on VSMC senescence need to be explored in future studies.

(5) Macrophage senescence induced by oxidized LDL also has an important role in atherosclerosis. In addition to the key link between dyslipidemia and inflammation that plays a vital role in macrophage senescence, oxidative stress, autophagy and apoptosis are also involved in this process. Further studies investigating the role of TRLs and the main types of FAs carried in TRLs on macrophage senescence are required.

(6) Senescent AMSCs and WAT display impaired capacities in lipid metabolism, insulin resistance and aberrant secretion of adipocytokines, which can interfere with the normal function of distal tissues through SASP and participate in atherogenesis. TRLs derived from a high‐calorie diet could induce AMSC senescence with increased inflammation and intercellular ROS.

## References

[brv12866-bib-0001] Abe, Y. , Kawakami, A. , Osaka, M. , Uematsu, S. , Akira, S. , Shimokado, K. , Sacks, F. M. & Yoshida, M. (2010). Apolipoprotein CIII induces monocyte chemoattractant protein‐1 and interleukin 6 expression via toll‐like receptor 2 pathway in mouse adipocytes. Arteriosclerosis, Thrombosis, and Vascular Biology 30(11), 2242–2248. 20829510 10.1161/ATVBAHA.110.210427 PMC3203842

[brv12866-bib-0002] Ahmad, F. & Leake, D. S. (2019). Lysosomal oxidation of LDL alters lysosomal pH, induces senescence, and increases secretion of pro‐inflammatory cytokines in human macrophages. Journal of Lipid Research 60(1), 98–110. 30397186 10.1194/jlr.M088245 PMC6314264

[brv12866-bib-0003] Ai, D. , Jiang, H. , Westerterp, M. , Murphy, A. J. , Wang, M. , Ganda, A. , Abramowicz, S. , Welch, C. , Almazan, F. , Zhu, Y. , Miller, Y. I. & Tall, A. R. (2014). Disruption of mammalian target of rapamycin complex 1 in macrophages decreases chemokine gene expression and atherosclerosis. Circulation Research 114(10), 1576–1584. 24687132 10.1161/CIRCRESAHA.114.302313 PMC4058053

[brv12866-bib-0004] Akhmedov, A. , Rozenberg, I. , Paneni, F. , Camici, G. G. , Shi, Y. , Doerries, C. , Sledzinska, A. , Mocharla, P. , Breitenstein, A. , Lohmann, C. , Stein, S. , von Lukowicz, T. , Kurrer, M. O. , Boren, J. , Becher, B. , et al. (2014). Endothelial overexpression of LOX‐1 increases plaque formation and promotes atherosclerosis in vivo. European Heart Journal 35(40), 2839–2848. 10.1093/eurheartj/eht532 24419805

[brv12866-bib-0005] Akhmedov, A. , Camici, G. G. , Reiner, M. F. , Bonetti, N. R. , Costantino, S. , Holy, E. W. , Spescha, R. D. , Stivala, S. , Schaub Clerigue, A. , Speer, T. , Breitenstein, A. , Manz, J. , Lohmann, C. , Paneni, F. , Beer, J. H. , et al. (2017). Endothelial LOX‐1 activation differentially regulates arterial thrombus formation depending on oxLDL levels: role of the Oct‐1/SIRT1 and ERK1/2 pathways. Cardiovascscular Research 113(5), 498–507. 10.1093/cvr/cvx015 28199510

[brv12866-bib-0006] Akhmedov, A. , Sawamura, T. , Chen, C. H. , Kraler, S. , Vdovenko, D. & Luscher, T. F. (2021). Lectin‐like oxidized low‐density lipoprotein receptor‐1 (LOX‐1): a crucial driver of atherosclerotic cardiovascular disease. European Heart Journal 42(18), 1797–1807. 33159784 10.1093/eurheartj/ehaa770

[brv12866-bib-0007] Allahverdian, S. , Chaabane, C. , Boukais, K. , Francis, G. A. & Bochaton‐Piallat, M. L. (2018). Smooth muscle cell fate and plasticity in atherosclerosis. Cardiovascular Research 114(4), 540–550. 29385543 10.1093/cvr/cvy022 PMC5852505

[brv12866-bib-0008] Arsenault, B. J. , Rana, J. S. , Stroes, E. S. , Despres, J. P. , Shah, P. K. , Kastelein, J. J. , Wareham, N. J. , Boekholdt, S. M. & Khaw, K. T. (2009). Beyond low‐density lipoprotein cholesterol: respective contributions of non‐high‐density lipoprotein cholesterol levels, triglycerides, and the total cholesterol/high‐density lipoprotein cholesterol ratio to coronary heart disease risk in apparently healthy men and women. Journal of the American College of Cardiology 55(1), 35–41. 20117361 10.1016/j.jacc.2009.07.057

[brv12866-bib-0009] Arunachalam, G. , Samuel, S. M. , Marei, I. , Ding, H. & Triggle, C. R. (2014). Metformin modulates hyperglycaemia‐induced endothelial senescence and apoptosis through SIRT1. British Journal of Pharmacology 171(2), 523–535. 24372553 10.1111/bph.12496 PMC3904269

[brv12866-bib-0010] Assmus, B. , Urbich, C. , Aicher, A. , Hofmann, W. K. , Haendeler, J. , Rossig, L. , Spyridopoulos, I. , Zeiher, A. M. & Dimmeler, S. (2003). HMG‐CoA reductase inhibitors reduce senescence and increase proliferation of endothelial progenitor cells via regulation of cell cycle regulatory genes. Circulation Research 92(9), 1049–1055. 12676819 10.1161/01.RES.0000070067.64040.7C

[brv12866-bib-0011] Baker, D. J. , Wijshake, T. , Tchkonia, T. , LeBrasseur, N. K. , Childs, B. G. , van de Sluis, B. , Kirkland, J. L. & van Deursen, J. M. (2011). Clearance of p16Ink4a‐positive senescent cells delays ageing‐associated disorders. Nature 479(7372), 232–236. 22048312 10.1038/nature10600 PMC3468323

[brv12866-bib-0012] Barter, P. J. , Caulfield, M. , Eriksson, M. , Grundy, S. M. , Kastelein, J. J. , Komajda, M. , Lopez‐Sendon, J. , Mosca, L. , Tardif, J. C. , Waters, D. D. , Shear, C. L. , Revkin, J. H. , Buhr, K. A. , Fisher, M. R. , Tall, A. R. , et al. (2007). Effects of torcetrapib in patients at high risk for coronary events. The New England Journal of Medicine 357(21), 2109–2122. 17984165 10.1056/NEJMoa0706628

[brv12866-bib-0013] Basatemur, G. L. , Jorgensen, H. F. , Clarke, M. C. H. , Bennett, M. R. & Mallat, Z. (2019). Vascular smooth muscle cells in atherosclerosis. Nature Reviews Cardiology 16(12), 727–744. 31243391 10.1038/s41569-019-0227-9

[brv12866-bib-0014] Bekkering, S. , Quintin, J. , Joosten, L. A. , van der Meer, J. W. , Netea, M. G. & Riksen, N. P. (2014). Oxidized low‐density lipoprotein induces long‐term proinflammatory cytokine production and foam cell formation via epigenetic reprogramming of monocytes. Arteriosclerosis, Thrombosis, and Vascular Biology 34(8), 1731–1738. 24903093 10.1161/ATVBAHA.114.303887

[brv12866-bib-0015] Belcastro, E. , Rehman, A. U. , Remila, L. , Park, S. H. , Gong, D. S. , Anton, N. , Auger, C. , Lefebvre, O. , Goetz, J. G. , Collot, M. , Klymchenko, A. S. , Vandamme, T. F. & Schini‐Kerth, V. B. (2021). Fluorescent nanocarriers targeting VCAM‐1 for early detection of senescent endothelial cells. Nanomedicine 34, 102379. 33713860 10.1016/j.nano.2021.102379

[brv12866-bib-0016] Bennett, M. R. , Macdonald, K. , Chan, S. W. , Boyle, J. J. & Weissberg, P. L. (1998). Cooperative interactions between RB and p53 regulate cell proliferation, cell senescence, and apoptosis in human vascular smooth muscle cells from atherosclerotic plaques. Circulation Research 82(6), 704–712. 9546379 10.1161/01.res.82.6.704

[brv12866-bib-0017] Bermudez, B. , Lopez, S. , Pacheco, Y. M. , Villar, J. , Muriana, F. J. , Hoheisel, J. D. , Bauer, A. & Abia, R. (2008). Influence of postprandial triglyceride‐rich lipoproteins on lipid‐mediated gene expression in smooth muscle cells of the human coronary artery. Cardiovascular Research 79(2), 294–303. 18359786 10.1093/cvr/cvn082

[brv12866-bib-0018] Bhayadia, R. , Schmidt, B. M. , Melk, A. & Homme, M. (2016). Senescence‐induced oxidative stress causes endothelial dysfunction. The Journals of Gerontology, Series A, Biological Sciences and Medical Sciences 71(2), 161–169. 25735595 10.1093/gerona/glv008

[brv12866-bib-0019] Bian, W. , Jing, X. , Yang, Z. , Shi, Z. , Chen, R. , Xu, A. , Wang, N. , Jiang, J. , Yang, C. , Zhang, D. , Li, L. , Wang, H. , Wang, J. , Sun, Y. & Zhang, C. (2020). Downregulation of LncRNA NORAD promotes ox‐LDL‐induced vascular endothelial cell injury and atherosclerosis. Aging (Albany NY) 12(7), 6385–6400. 32267831 10.18632/aging.103034 PMC7185106

[brv12866-bib-0020] Bonkowski, M. S. & Sinclair, D. A. (2016). Slowing ageing by design: the rise of NAD(+) and sirtuin‐activating compounds. Nature Reviews Molecular Cell Biology 17(11), 679–690. 27552971 10.1038/nrm.2016.93 PMC5107309

[brv12866-bib-0021] Boren, J. , Chapman, M. J. , Krauss, R. M. , Packard, C. J. , Bentzon, J. F. , Binder, C. J. , Daemen, M. J. , Demer, L. L. , Hegele, R. A. , Nicholls, S. J. , Nordestgaard, B. G. , Watts, G. F. , Bruckert, E. , Fazio, S. , Ference, B. A. , et al. (2020). Low‐density lipoproteins cause atherosclerotic cardiovascular disease: pathophysiological, genetic, and therapeutic insights: a consensus statement from the European Atherosclerosis Society Consensus Panel. European Heart Journal 41(24), 2313–2330. 32052833 10.1093/eurheartj/ehz962 PMC7308544

[brv12866-bib-0022] Botham, K. M. & Wheeler‐Jones, C. P. (2013). Postprandial lipoproteins and the molecular regulation of vascular homeostasis. Progress in Lipid Research 52(4), 446–464. 23774609 10.1016/j.plipres.2013.06.001

[brv12866-bib-0023] Botham, K. M. , Moore, E. H. , De Pascale, C. & Bejta, F. (2007). The induction of macrophage foam cell formation by chylomicron remnants. Biochemical Society Transactions 35(Pt. 3), 454–458. 17511626 10.1042/BST0350454

[brv12866-bib-0024] Brodeur, M. R. , Bouvet, C. , Barrette, M. & Moreau, P. (2013). Palmitic acid increases medial calcification by inducing oxidative stress. Journal of Vascular Research 50(5), 430–441. 24080574 10.1159/000354235

[brv12866-bib-0025] Burton, D. G. A. & Faragher, R. G. A. (2018). Obesity and type‐2 diabetes as inducers of premature cellular senescence and ageing. Biogerontology 19(6), 447–459. 30054761 10.1007/s10522-018-9763-7 PMC6223730

[brv12866-bib-0026] Burton, D. G. , Matsubara, H. & Ikeda, K. (2010). Pathophysiology of vascular calcification: pivotal role of cellular senescence in vascular smooth muscle cells. Experimental Gerontology 45(11), 819–824. 20647039 10.1016/j.exger.2010.07.005

[brv12866-bib-0027] Cai, Z. , Ding, Y. , Zhang, M. , Lu, Q. , Wu, S. , Zhu, H. , Song, P. & Zou, M. H. (2016). Ablation of adenosine monophosphate‐activated protein kinase alpha1 in vascular smooth muscle cells promotes diet‐induced atherosclerotic calcification in vivo. Circulation Research 119(3), 422–433. 27256105 10.1161/CIRCRESAHA.116.308301

[brv12866-bib-0028] Campisi, J. , Kapahi, P. , Lithgow, G. J. , Melov, S. , Newman, J. C. & Verdin, E. (2019). From discoveries in ageing research to therapeutics for healthy ageing. Nature 571(7764), 183–192. 31292558 10.1038/s41586-019-1365-2 PMC7205183

[brv12866-bib-0029] Cannon, C. P. , Braunwald, E. , McCabe, C. H. , Rader, D. J. , Rouleau, J. L. , Belder, R. , Joyal, S. V. , Hill, K. A. , Pfeffer, M. A. , Skene, A. M. & Pravastatin or Atorvastatin Evaluation and Infection Therapy‐Thrombolysis in Myocardial Infarction 22 Investigators (2004). Intensive versus moderate lipid lowering with statins after acute coronary syndromes. The New England Journal of Medicine 350(15), 1495–1504. 15007110 10.1056/NEJMoa040583

[brv12866-bib-0030] Cao, H. , Jia, Q. , Yan, L. , Chen, C. , Xing, S. & Shen, D. (2019). Quercetin suppresses the progression of atherosclerosis by regulating MST1‐mediated autophagy in ox‐LDL‐induced RAW264.7 macrophage foam cells. International Journal of Molecular Sciences 20(23), 6093. 10.3390/ijms20236093 PMC6928812 31816893

[brv12866-bib-0031] Cardus, A. , Uryga, A. K. , Walters, G. & Erusalimsky, J. D. (2013). SIRT6 protects human endothelial cells from DNA damage, telomere dysfunction, and senescence. Cardiovascular Research 97(3), 571–579. 23201774 10.1093/cvr/cvs352 PMC3567786

[brv12866-bib-0032] Carracedo, J. , Merino, A. , Briceno, C. , Soriano, S. , Buendia, P. , Calleros, L. , Rodriguez, M. , Martin‐Malo, A. , Aljama, P. & Ramirez, R. (2011). Carbamylated low‐density lipoprotein induces oxidative stress and accelerated senescence in human endothelial progenitor cells. FASEB Journal 25(4), 1314–1322. 21228221 10.1096/fj.10-173377

[brv12866-bib-0033] Chatterjee, T. K. , Stoll, L. L. , Denning, G. M. , Harrelson, A. , Blomkalns, A. L. , Idelman, G. , Rothenberg, F. G. , Neltner, B. , Romig‐Martin, S. A. , Dickson, E. W. , Rudich, S. & Weintraub, N. L. (2009). Proinflammatory phenotype of perivascular adipocytes: influence of high‐fat feeding. Circulation Research 104(4), 541–549. 19122178 10.1161/CIRCRESAHA.108.182998 PMC2742882

[brv12866-bib-0034] Chava, K. R. , Karpurapu, M. , Wang, D. , Bhanoori, M. , Kundumani‐Sridharan, V. , Zhang, Q. , Ichiki, T. , Glasgow, W. C. & Rao, G. N. (2009). CREB‐mediated IL‐6 expression is required for 15(S)‐hydroxyeicosatetraenoic acid‐induced vascular smooth muscle cell migration. Arteriosclerosis, Thrombosis, and Vascular Biology 29(6), 809–815. 19342597 10.1161/ATVBAHA.109.185777 PMC2724759

[brv12866-bib-0035] Childs, B. G. , Baker, D. J. , Wijshake, T. , Conover, C. A. , Campisi, J. & van Deursen, J. M. (2016). Senescent intimal foam cells are deleterious at all stages of atherosclerosis. Science 354(6311), 472–477. 27789842 10.1126/science.aaf6659 PMC5112585

[brv12866-bib-0036] Chiu, S. C. , Chiang, E. P. , Tsai, S. Y. , Wang, F. Y. , Pai, M. H. , Syu, J. N. , Cheng, C. C. , Rodriguez, R. L. & Tang, F. Y. (2014). Eicosapentaenoic acid induces neovasculogenesis in human endothelial progenitor cells by modulating c‐kit protein and PI3‐K/Akt/eNOS signaling pathways. The Journal of Nutritional Biochemistry 25(9), 934–945. 24927915 10.1016/j.jnutbio.2014.04.007

[brv12866-bib-0037] Chiu, S. C. , Chao, C. Y. , Chiang, E. I. , Syu, J. N. , Rodriguez, R. L. & Tang, F. Y. (2017). N‐3 polyunsaturated fatty acids alleviate high glucose‐mediated dysfunction of endothelial progenitor cells and prevent ischemic injuries both in vitro and in vivo. The Journal of Nutritional Biochemistry 42, 172–181. 28189115 10.1016/j.jnutbio.2017.01.009

[brv12866-bib-0038] Cho, K. & Choi, S. H. (2019). ASK1 mediates apoptosis and autophagy during oxLDL‐CD36 signaling in senescent endothelial cells. Oxidative Medicine and Cellular Longevity 2019, 2840437. 31772703 10.1155/2019/2840437 PMC6854215

[brv12866-bib-0039] de la Cruz‐Ares, S. , Cardelo, M. P. , Gutierrez‐Mariscal, F. M. , Torres‐Pena, J. D. , Garcia‐Rios, A. , Katsiki, N. , Malagon, M. M. , Lopez‐Miranda, J. , Perez‐Martinez, P. & Yubero‐Serrano, E. M. (2020). Endothelial dysfunction and advanced glycation end products in patients with newly diagnosed versus established diabetes: from the CORDIOPREV study. Nutrients 12(1), 238. 10.3390/nu12010238 PMC7019746 31963378

[brv12866-bib-0040] Csiszar, A. , Wang, M. , Lakatta, E. G. & Ungvari, Z. (2008). Inflammation and endothelial dysfunction during aging: role of NF‐kappaB. Journal of Applied Physiology (Bethesda, Md.: 1985) 105(4), 1333–1341. 10.1152/japplphysiol.90470.2008 PMC2576023 18599677

[brv12866-bib-0041] Dai, H. , Sinclair, D. A. , Ellis, J. L. & Steegborn, C. (2018). Sirtuin activators and inhibitors: promises, achievements, and challenges. Pharmacology & Therapeutics 188, 140–154. 29577959 10.1016/j.pharmthera.2018.03.004 PMC6342514

[brv12866-bib-0042] De Castellarnau, C. , Sanchez‐Quesada, J. L. , Benitez, S. , Rosa, R. , Caveda, L. , Vila, L. & Ordonez‐Llanos, J. (2000). Electronegative LDL from normolipemic subjects induces IL‐8 and monocyte chemotactic protein secretion by human endothelial cells. Arteriosclerosis, Thrombosis, and Vascular Biology 20(10), 2281–2287. 11031216 10.1161/01.atv.20.10.2281

[brv12866-bib-0043] De Meyer, G. R. , Grootaert, M. O. , Michiels, C. F. , Kurdi, A. , Schrijvers, D. M. & Martinet, W. (2015). Autophagy in vascular disease. Circulation Research 116(3), 468–479. 25634970 10.1161/CIRCRESAHA.116.303804

[brv12866-bib-0044] De Pascale, C. , Graham, V. , Fowkes, R. C. , Wheeler‐Jones, C. P. & Botham, K. M. (2009). Suppression of nuclear factor‐kappaB activity in macrophages by chylomicron remnants: modulation by the fatty acid composition of the particles. The FEBS Journal 276(19), 5689–5702. 19725874 10.1111/j.1742-4658.2009.07260.x PMC2776925

[brv12866-bib-0045] Di Pietro, N. , Formoso, G. & Pandolfi, A. (2016). Physiology and pathophysiology of oxLDL uptake by vascular wall cells in atherosclerosis. Vascular Pharmacology 84, 1–7. 27256928 10.1016/j.vph.2016.05.013

[brv12866-bib-0046] Di Santo, S. , Diehm, N. , Ortmann, J. , Volzmann, J. , Yang, Z. , Keo, H. H. , Baumgartner, I. & Kalka, C. (2008). Oxidized low density lipoprotein impairs endothelial progenitor cell function by downregulation of E‐selectin and integrin alpha(v)beta5. Biochemical and Biophysical Research Communications 373(4), 528–532. 18590706 10.1016/j.bbrc.2008.06.066

[brv12866-bib-0047] Dichtl, W. , Dulak, J. , Frick, M. , Alber, H. F. , Schwarzacher, S. P. , Ares, M. P. , Nilsson, J. , Pachinger, O. & Weidinger, F. (2003). HMG‐CoA reductase inhibitors regulate inflammatory transcription factors in human endothelial and vascular smooth muscle cells. Arteriosclerosis, Thrombosis, and Vascular Biology 23(1), 58–63. 12524225 10.1161/01.atv.0000043456.48735.20

[brv12866-bib-0048] Ding, Z. , Wang, X. , Schnackenberg, L. , Khaidakov, M. , Liu, S. , Singla, S. , Dai, Y. & Mehta, J. L. (2013). Regulation of autophagy and apoptosis in response to ox‐LDL in vascular smooth muscle cells, and the modulatory effects of the microRNA hsa‐let‐7 g. International Journal of Cardiology 168(2), 1378–1385. 23305858 10.1016/j.ijcard.2012.12.045

[brv12866-bib-0049] Domoto, K. , Taniguchi, T. , Takaishi, H. , Takahashi, T. , Fujioka, Y. , Takahashi, A. , Ishikawa, Y. & Yokoyama, M. (2003). Chylomicron remnants induce monocyte chemoattractant protein‐1 expression via p38 MAPK activation in vascular smooth muscle cells. Atherosclerosis 171(2), 193–200. 14644387 10.1016/j.atherosclerosis.2003.08.016

[brv12866-bib-0050] Donato, A. J. , Gano, L. B. , Eskurza, I. , Silver, A. E. , Gates, P. E. , Jablonski, K. & Seals, D. R. (2009). Vascular endothelial dysfunction with aging: endothelin‐1 and endothelial nitric oxide synthase. American Journal of Physiology. Heart and Circulatory Physiology 297(1), H425–H432. 19465546 10.1152/ajpheart.00689.2008 PMC2711733

[brv12866-bib-0051] Donato, A. J. , Morgan, R. G. , Walker, A. E. & Lesniewski, L. A. (2015). Cellular and molecular biology of aging endothelial cells. Journal of Molecular and Cellular Cardiology 89(Pt. B), 122–135. 25655936 10.1016/j.yjmcc.2015.01.021 PMC4522407

[brv12866-bib-0052] Fadini, G. P. , Baesso, I. , Albiero, M. , Sartore, S. , Agostini, C. & Avogaro, A. (2008). Technical notes on endothelial progenitor cells: ways to escape from the knowledge plateau. Atherosclerosis 197(2), 496–503. 18249408 10.1016/j.atherosclerosis.2007.12.039

[brv12866-bib-0053] Ferretti, G. , Bacchetti, T. , Negre‐Salvayre, A. , Salvayre, R. , Dousset, N. & Curatola, G. (2006). Structural modifications of HDL and functional consequences. Atherosclerosis 184(1), 1–7. 16157342 10.1016/j.atherosclerosis.2005.08.008

[brv12866-bib-0054] Foreman, K. E. & Tang, J. (2003). Molecular mechanisms of replicative senescence in endothelial cells. Experimental Gerontology 38(11–12), 1251–1257. 14698804 10.1016/j.exger.2003.09.005

[brv12866-bib-0055] Forouzandeh, F. , Salazar, G. , Patrushev, N. , Xiong, S. , Hilenski, L. , Fei, B. & Alexander, R. W. (2014). Metformin beyond diabetes: pleiotropic benefits of metformin in attenuation of atherosclerosis. Journal of the American Heart Association 3(6), e001202. 25527624 10.1161/JAHA.114.001202 PMC4338706

[brv12866-bib-0056] Fujioka, Y. & Ishikawa, Y. (2009). Remnant lipoproteins as strong key particles to atherogenesis. Journal of Atherosclerosis and Thrombosis 16(3), 145–154. 19556722 10.5551/jat.e598

[brv12866-bib-0057] Gardner, S. E. , Humphry, M. , Bennett, M. R. & Clarke, M. C. (2015). Senescent vascular smooth muscle cells drive inflammation through an interleukin‐1alpha‐dependent senescence‐associated secretory phenotype. Arteriosclerosis, Thrombosis, and Vascular Biology 35(9), 1963–1974. 26139463 10.1161/ATVBAHA.115.305896 PMC4548545

[brv12866-bib-0058] GBD 2013 Mortality and Causes of Death Collaborators (2015). Global, regional, and national age‐sex specific all‐cause and cause‐specific mortality for 240 causes of death, 1990–2013: a systematic analysis for the Global Burden of Disease Study 2013. The Lancet 385(9963), 117–171. 10.1016/S0140-6736(14)61682-2 PMC4340604 25530442

[brv12866-bib-0059] Ghanbari, M. , Momen Maragheh, S. , Aghazadeh, A. , Mehrjuyan, S. R. , Hussen, B. M. , Abdoli Shadbad, M. , Dastmalchi, N. & Safaralizadeh, R. (2021). Interleukin‐1 in obesity‐related low‐grade inflammation: from molecular mechanisms to therapeutic strategies. International Immunopharmacology 96, 107765. 34015596 10.1016/j.intimp.2021.107765

[brv12866-bib-0060] Gimbrone, M. A. Jr. & Garcia‐Cardena, G. (2016). Endothelial cell dysfunction and the pathobiology of atherosclerosis. Circulation Research 118(4), 620–636. 26892962 10.1161/CIRCRESAHA.115.306301 PMC4762052

[brv12866-bib-0061] Glynn, R. J. , Danielson, E. , Fonseca, F. A. , Genest, J. , Gotto, A. M. Jr. , Kastelein, J. J. , Koenig, W. , Libby, P. , Lorenzatti, A. J. , MacFadyen, J. G. , Nordestgaard, B. G. , Shepherd, J. , Willerson, J. T. & Ridker, P. M. (2009). A randomized trial of rosuvastatin in the prevention of venous thromboembolism. The New England Journal of Medicine 360(18), 1851–1861. 19329822 10.1056/NEJMoa0900241 PMC2710995

[brv12866-bib-0062] Gong, X. , Ma, Y. , Ruan, Y. , Fu, G. & Wu, S. (2014). Long‐term atorvastatin improves age‐related endothelial dysfunction by ameliorating oxidative stress and normalizing eNOS/iNOS imbalance in rat aorta. Experimental Gerontology 52, 9–17. 24463049 10.1016/j.exger.2014.01.015

[brv12866-bib-0063] Goodpaster, B. H. , Krishnaswami, S. , Resnick, H. , Kelley, D. E. , Haggerty, C. , Harris, T. B. , Schwartz, A. V. , Kritchevsky, S. & Newman, A. B. (2003). Association between regional adipose tissue distribution and both type 2 diabetes and impaired glucose tolerance in elderly men and women. Diabetes Care 26(2), 372–379. 12547865 10.2337/diacare.26.2.372

[brv12866-bib-0064] Gorenne, I. , Kavurma, M. , Scott, S. & Bennett, M. (2006). Vascular smooth muscle cell senescence in atherosclerosis. Cardiovascular Research 72(1), 9–17. 16824498 10.1016/j.cardiores.2006.06.004

[brv12866-bib-0065] Gorenne, I. , Kumar, S. , Gray, K. , Figg, N. , Yu, H. , Mercer, J. & Bennett, M. (2013). Vascular smooth muscle cell sirtuin 1 protects against DNA damage and inhibits atherosclerosis. Circulation 127(3), 386–396. 23224247 10.1161/CIRCULATIONAHA.112.124404

[brv12866-bib-0066] Grootaert, M. O. J. , Moulis, M. , Roth, L. , Martinet, W. , Vindis, C. , Bennett, M. R. & De Meyer, G. R. Y. (2018). Vascular smooth muscle cell death, autophagy and senescence in atherosclerosis. Cardiovascular Research 114(4), 622–634. 29360955 10.1093/cvr/cvy007

[brv12866-bib-0067] Grootaert, M. O. J. , Finigan, A. , Figg, N. L. , Uryga, A. K. & Bennett, M. R. (2021). SIRT6 protects smooth muscle cells from senescence and reduces atherosclerosis. Circulation Research 128(4), 474–491. 33353368 10.1161/CIRCRESAHA.120.318353 PMC7899748

[brv12866-bib-0068] Gu, W. , Nowak, W. N. , Xie, Y. , Le Bras, A. , Hu, Y. , Deng, J. , Issa Bhaloo, S. , Lu, Y. , Yuan, H. , Fidanis, E. , Saxena, A. , Kanno, T. , Mason, A. J. , Dulak, J. , Cai, J. , et al. (2019). Single‐cell RNA‐sequencing and metabolomics analyses reveal the contribution of perivascular adipose tissue stem cells to vascular remodeling. Arteriosclerosis, Thrombosis, and Vascular Biology 39(10), 2049–2066. 31340667 10.1161/ATVBAHA.119.312732 PMC6766361

[brv12866-bib-0069] Gustafson, B. , Nerstedt, A. & Smith, U. (2019). Reduced subcutaneous adipogenesis in human hypertrophic obesity is linked to senescent precursor cells. Nature Communications 10(1), 2757. 10.1038/s41467-019-10688-x PMC6588633 31227697

[brv12866-bib-0070] Hamczyk, M. R. , del Campo, L. & Andres, V. (2018a). Aging in the cardiovascular system: lessons from Hutchinson‐Gilford progeria syndrome. Annual Review of Physiology 80, 27–48. 10.1146/annurev-physiol-021317-121454 28934587

[brv12866-bib-0071] Hamczyk, M. R. , Villa‐Bellosta, R. , Gonzalo, P. , Andres‐Manzano, M. J. , Nogales, P. , Bentzon, J. F. , Lopez‐Otin, C. & Andres, V. (2018b). Vascular smooth muscle‐specific progerin expression accelerates atherosclerosis and death in a mouse model of Hutchinson‐Gilford progeria syndrome. Circulation 138(3), 266–282. 29490993 10.1161/CIRCULATIONAHA.117.030856 PMC6075893

[brv12866-bib-0072] Hasegawa, Y. , Saito, T. , Ogihara, T. , Ishigaki, Y. , Yamada, T. , Imai, J. , Uno, K. , Gao, J. , Kaneko, K. , Shimosawa, T. , Asano, T. , Fujita, T. , Oka, Y. & Katagiri, H. (2012). Blockade of the nuclear factor‐kappaB pathway in the endothelium prevents insulin resistance and prolongs life spans. Circulation 125(9), 1122–1133. 22302838 10.1161/CIRCULATIONAHA.111.054346

[brv12866-bib-0073] He, B. M. , Zhao, S. P. & Peng, Z. Y. (2013). Effects of cigarette smoking on HDL quantity and function: implications for atherosclerosis. Journal of Cellular Biochemistry 114(11), 2431–2436. 23852759 10.1002/jcb.24581

[brv12866-bib-0074] Hofmann Bowman, M. A. , Gawdzik, J. , Bukhari, U. , Husain, A. N. , Toth, P. T. , Kim, G. , Earley, J. & McNally, E. M. (2011). S100A12 in vascular smooth muscle accelerates vascular calcification in apolipoprotein E‐null mice by activating an osteogenic gene regulatory program. Arteriosclerosis, Thrombosis, and Vascular Biology 31(2), 337–344. 20966394 10.1161/ATVBAHA.110.217745 PMC3364048

[brv12866-bib-0075] Huang, C. Y. , Lin, F. Y. , Shih, C. M. , Au, H. K. , Chang, Y. J. , Nakagami, H. , Morishita, R. , Chang, N. C. , Shyu, K. G. & Chen, J. W. (2012). Moderate to high concentrations of high‐density lipoprotein from healthy subjects paradoxically impair human endothelial progenitor cells and related angiogenesis by activating Rho‐associated kinase pathways. Arteriosclerosis, Thrombosis, and Vascular Biology 32(10), 2405–2417. 22904272 10.1161/ATVBAHA.112.248617

[brv12866-bib-0076] Huang, C. S. , Lin, A. H. , Liu, C. T. , Tsai, C. W. , Chang, I. S. , Chen, H. W. & Lii, C. K. (2013). Isothiocyanates protect against oxidized LDL‐induced endothelial dysfunction by upregulating Nrf2‐dependent antioxidation and suppressing NFkappaB activation. Molecular Nutrition & Food Research 57(11), 1918–1930. 23836589 10.1002/mnfr.201300063

[brv12866-bib-0077] Huynh, J. , Nishimura, N. , Rana, K. , Peloquin, J. M. , Califano, J. P. , Montague, C. R. , King, M. R. , Schaffer, C. B. & Reinhart‐King, C. A. (2011). Age‐related intimal stiffening enhances endothelial permeability and leukocyte transmigration. Science Translational Medicine 3(112), 112ra122. 10.1126/scitranslmed.3002761 PMC3693751 22158860

[brv12866-bib-0078] Imanishi, T. , Hano, T. , Sawamura, T. & Nishio, I. (2004). Oxidized low‐density lipoprotein induces endothelial progenitor cell senescence, leading to cellular dysfunction. Clinical and Experimental Pharmacology & Physiology 31(7), 407–413. 15236625 10.1111/j.1440-1681.2004.04022.x

[brv12866-bib-0079] Imke, C. , Rodriguez, B. L. , Grove, J. S. , McNamara, J. R. , Waslien, C. , Katz, A. R. , Willcox, B. , Yano, K. & Curb, J. D. (2005). Are remnant‐like particles independent predictors of coronary heart disease incidence? The Honolulu heart study. Arteriosclerosis, Thrombosis, and Vascular Biology 25(8), 1718–1722. 15947240 10.1161/01.ATV.0000173310.85845.7b

[brv12866-bib-0080] Istvan, E. S. & Deisenhofer, J. (2001). Structural mechanism for statin inhibition of HMG‐CoA reductase. Science 292(5519), 1160–1164. 11349148 10.1126/science.1059344

[brv12866-bib-0081] Ji, K. T. , Qian, L. , Nan, J. L. , Xue, Y. J. , Zhang, S. Q. , Wang, G. Q. , Yin, R. P. , Zhu, Y. J. , Wang, L. P. , Ma, J. , Liao, L. M. & Tang, J. F. (2015). ox‐LDL induces dysfunction of endothelial progenitor cells via activation of NF‐kappaB. BioMed Research International 2015, 175291. 25821786 10.1155/2015/175291 PMC4363986

[brv12866-bib-0082] Jia, Q. , Cao, H. , Shen, D. , Yan, L. , Chen, C. & Xing, S. (2019). Fisetin, via CKIP‐1/REGgamma, limits oxidized LDL‐induced lipid accumulation and senescence in RAW264.7 macrophage‐derived foam cells. European Journal of Pharmacology 865, 172748. 31655030 10.1016/j.ejphar.2019.172748

[brv12866-bib-0083] Jiang, Y. H. , Jiang, L. Y. , Wang, Y. C. , Ma, D. F. & Li, X. (2020). Quercetin attenuates atherosclerosis via modulating oxidized LDL‐induced endothelial cellular senescence. Frontiers in Pharmacology 11, 512. 32410992 10.3389/fphar.2020.00512 PMC7198817

[brv12866-bib-0084] Jinno, Y. , Nakakuki, M. , Kawano, H. , Notsu, T. , Mizuguchi, K. & Imada, K. (2011). Eicosapentaenoic acid administration attenuates the pro‐inflammatory properties of VLDL by decreasing its susceptibility to lipoprotein lipase in macrophages. Atherosclerosis 219(2), 566–572. 22018640 10.1016/j.atherosclerosis.2011.09.046

[brv12866-bib-0085] Kageyama, A. , Matsui, H. , Ohta, M. , Sambuichi, K. , Kawano, H. , Notsu, T. , Imada, K. , Yokoyama, T. & Kurabayashi, M. (2013). Palmitic acid induces osteoblastic differentiation in vascular smooth muscle cells through ACSL3 and NF‐kappaB, novel targets of eicosapentaenoic acid. PLoS One 8(6), e68197. 23840832 10.1371/journal.pone.0068197 PMC3695932

[brv12866-bib-0086] Kane, A. E. & Sinclair, D. A. (2018). Sirtuins and NAD(+) in the development and treatment of metabolic and cardiovascular diseases. Circulation Research 123(7), 868–885. 30355082 10.1161/CIRCRESAHA.118.312498 PMC6206880

[brv12866-bib-0087] Karnewar, S. , Neeli, P. K. , Panuganti, D. , Kotagiri, S. , Mallappa, S. , Jain, N. , Jerald, M. K. & Kotamraju, S. (2018). Metformin regulates mitochondrial biogenesis and senescence through AMPK mediated H3K79 methylation: relevance in age‐associated vascular dysfunction. Biochimica et Biophysica Acta. Molecular Basis of Disease 1864(4 Pt. A), 1115–1128. 29366775 10.1016/j.bbadis.2018.01.018

[brv12866-bib-0088] Kasiske, B. L. , de Mattos, A. , Flechner, S. M. , Gallon, L. , Meier‐Kriesche, H. U. , Weir, M. R. & Wilkinson, A. (2008). Mammalian target of rapamycin inhibitor dyslipidemia in kidney transplant recipients. American Journal of Transplantation 8(7), 1384–1392. 18510633 10.1111/j.1600-6143.2008.02272.x

[brv12866-bib-0089] Kataoka, H. , Kume, N. , Miyamoto, S. , Minami, M. , Morimoto, M. , Hayashida, K. , Hashimoto, N. & Kita, T. (2001). Oxidized LDL modulates Bax/Bcl‐2 through the lectinlike ox‐LDL receptor‐1 in vascular smooth muscle cells. Arteriosclerosis, Thrombosis, and Vascular Biology 21(6), 955–960. 11397703 10.1161/01.atv.21.6.955

[brv12866-bib-0090] Kawakami, A. , Tanaka, A. , Chiba, T. , Nakajima, K. , Shimokado, K. & Yoshida, M. (2003). Remnant lipoprotein‐induced smooth muscle cell proliferation involves epidermal growth factor receptor transactivation. Circulation 108(21), 2679–2688. 14623816 10.1161/01.CIR.0000093278.75565.87

[brv12866-bib-0091] Kawasaki, S. , Taniguchi, T. , Fujioka, Y. , Takahashi, A. , Takahashi, T. , Domoto, K. , Taguchi, M. , Ishikawa, Y. & Yokoyama, M. (2000). Chylomicron remnant induces apoptosis in vascular endothelial cells. Annals of the New York Academy of Sciences 902, 336–341. 10865859 10.1111/j.1749-6632.2000.tb06334.x

[brv12866-bib-0092] Keymel, S. , Kalka, C. , Rassaf, T. , Yeghiazarians, Y. , Kelm, M. & Heiss, C. (2008). Impaired endothelial progenitor cell function predicts age‐dependent carotid intimal thickening. Basic Research in Cardiology 103(6), 582–586. 18704258 10.1007/s00395-008-0742-z

[brv12866-bib-0093] Khoukaz, H. B. , Ji, Y. , Braet, D. J. , Vadali, M. , Abdelhamid, A. A. , Emal, C. D. , Lawrence, D. A. & Fay, W. P. (2020). Drug targeting of plasminogen activator inhibitor‐1 inhibits metabolic dysfunction and atherosclerosis in a murine model of metabolic syndrome. Arteriosclerosis, Thrombosis, and Vascular Biology 40(6), 1479–1490. 32268785 10.1161/ATVBAHA.119.313775 PMC7255962

[brv12866-bib-0094] Kilhovd, B. K. , Juutilainen, A. , Lehto, S. , Ronnemaa, T. , Torjesen, P. A. , Birkeland, K. I. , Berg, T. J. , Hanssen, K. F. & Laakso, M. (2005). High serum levels of advanced glycation end products predict increased coronary heart disease mortality in nondiabetic women but not in nondiabetic men: a population‐based 18‐year follow‐up study. Arteriosclerosis, Thrombosis, and Vascular Biology 25(4), 815–820. 15692098 10.1161/01.ATV.0000158380.44231.fe

[brv12866-bib-0095] Kim, S. G. , Sung, J. Y. , Kim, J. R. & Choi, H. C. (2020). Quercetin‐induced apoptosis ameliorates vascular smooth muscle cell senescence through AMP‐activated protein kinase signaling pathway. The Korean Journal of Physiology & Pharmacology 24(1), 69–79. 31908576 10.4196/kjpp.2020.24.1.69 PMC6940493

[brv12866-bib-0096] Kiss, T. , Giles, C. B. , Tarantini, S. , Yabluchanskiy, A. , Balasubramanian, P. , Gautam, T. , Csipo, T. , Nyul‐Toth, A. , Lipecz, A. , Szabo, C. , Farkas, E. , Wren, J. D. , Csiszar, A. & Ungvari, Z. (2019). Nicotinamide mononucleotide (NMN) supplementation promotes anti‐aging miRNA expression profile in the aorta of aged mice, predicting epigenetic rejuvenation and anti‐atherogenic effects. GeroScience 41(4), 419–439. 31463647 10.1007/s11357-019-00095-x PMC6815288

[brv12866-bib-0097] Kiyan, Y. , Tkachuk, S. , Hilfiker‐Kleiner, D. , Haller, H. , Fuhrman, B. & Dumler, I. (2014). oxLDL induces inflammatory responses in vascular smooth muscle cells via urokinase receptor association with CD36 and TLR4. Journal of Molecular and Cellular Cardiology 66, 72–82. 24239845 10.1016/j.yjmcc.2013.11.005

[brv12866-bib-0098] Kooy, A. , de Jager, J. , Lehert, P. , Bets, D. , Wulffele, M. G. , Donker, A. J. & Stehouwer, C. D. (2009). Long‐term effects of metformin on metabolism and microvascular and macrovascular disease in patients with type 2 diabetes mellitus. Archives of Internal Medicine 169(6), 616–625. 19307526 10.1001/archinternmed.2009.20

[brv12866-bib-0099] Kuki, S. , Imanishi, T. , Kobayashi, K. , Matsuo, Y. , Obana, M. & Akasaka, T. (2006). Hyperglycemia accelerated endothelial progenitor cell senescence via the activation of p38 mitogen‐activated protein kinase. Circulation Journal 70(8), 1076–1081. 16864945 10.1253/circj.70.1076

[brv12866-bib-0100] Kurdi, A. , Martinet, W. & De Meyer, G. R. Y. (2018). mTOR inhibition and cardiovascular diseases: dyslipidemia and atherosclerosis. Transplantation 102(2S Suppl. 1), S44–S46. 28230638 10.1097/TP.0000000000001693

[brv12866-bib-0101] Lai, P. & Liu, Y. (2015). Angelica sinensis polysaccharides inhibit endothelial progenitor cell senescence through the reduction of oxidative stress and activation of the Akt/hTERT pathway. Pharmaceutical Biology 53(12), 1842–1849. 25845638 10.3109/13880209.2015.1027779

[brv12866-bib-0102] Latham Birt, S. H. , Purcell, R. , Botham, K. M. & Wheeler‐Jones, C. P. (2016). Endothelial HO‐1 induction by model TG‐rich lipoproteins is regulated through a NOX4‐Nrf2 pathway. Journal of Lipid Research 57(7), 1204–1218. 27185859 10.1194/jlr.M067108 PMC4918850

[brv12866-bib-0103] Lee, C. F. , Qiao, M. , Schroder, K. , Zhao, Q. & Asmis, R. (2010). Nox4 is a novel inducible source of reactive oxygen species in monocytes and macrophages and mediates oxidized low density lipoprotein‐induced macrophage death. Circulation Research 106(9), 1489–1497. 20360249 10.1161/CIRCRESAHA.109.215392 PMC2924578

[brv12866-bib-0104] Lee, M. J. , Kim, E. H. , Lee, S. A. , Kang, Y. M. , Jung, C. H. , Yoon, H. K. , Seol, S. M. , Lee, Y. L. , Lee, W. J. & Park, J. Y. (2015). Dehydroepiandrosterone prevents linoleic acid‐induced endothelial cell senescence by increasing autophagy. Metabolism 64(9), 1134–1145. 26051603 10.1016/j.metabol.2015.05.006

[brv12866-bib-0105] Li, S. , Cao, H. , Shen, D. , Jia, Q. , Chen, C. & Xing, S. L. (2018). Quercetin protects against oxLDLinduced injury via regulation of ABCAl, LXRalpha and PCSK9 in RAW264.7 macrophages. Molecular Medicine Reports 18(1), 799–806. 29845234 10.3892/mmr.2018.9048 PMC6059709

[brv12866-bib-0106] Li, Y. , Peng, Z. , Wang, C. , Li, L. , Leng, Y. , Chen, R. , Yuan, H. , Zhou, S. , Zhang, Z. & Chen, A. F. (2018a). Novel role of PKR in palmitate‐induced Sirt1 inactivation and endothelial cell senescence. American Journal of Physiology. Heart and Circulatory Physiology 315(3), H571–H580. 29906232 10.1152/ajpheart.00038.2018

[brv12866-bib-0107] Li, Y. , Qin, R. , Yan, H. , Wang, F. , Huang, S. , Zhang, Y. , Zhong, M. , Zhang, W. & Wang, Z. (2018b). Inhibition of vascular smooth muscle cells premature senescence with rutin attenuates and stabilizes diabetic atherosclerosis. The Journal of Nutritional Biochemistry 51, 91–98. 29107826 10.1016/j.jnutbio.2017.09.012

[brv12866-bib-0108] Li, X. , Sun, X. & Carmeliet, P. (2019). Hallmarks of endothelial cell metabolism in health and disease. Cell Metabolism 30(3), 414–433. 31484054 10.1016/j.cmet.2019.08.011

[brv12866-bib-0109] Li, Z. , Xu, K. , Zhao, S. , Guo, Y. , Chen, H. , Ni, J. , Liu, Q. & Wang, Z. (2021). SPATA4 improves aging‐induced metabolic dysfunction through promotion of preadipocyte differentiation and adipose tissue expansion. Aging Cell 20(1), e13282. 33314576 10.1111/acel.13282 PMC7811838

[brv12866-bib-0110] Liang, C. , Wang, Q. S. , Yang, X. , Niu, N. , Hu, Q. Q. , Zhang, B. L. , Wu, M. M. , Yu, C. J. , Chen, X. , Song, B. L. , Zhang, Z. R. & Ma, H. P. (2018). Oxidized low‐density lipoprotein stimulates epithelial sodium channels in endothelial cells of mouse thoracic aorta. British Journal of Pharmacology 175(8), 1318–1328. 28480509 10.1111/bph.13853 PMC5866960

[brv12866-bib-0111] Liao, L. , Zhou, Q. , Song, Y. , Wu, W. , Yu, H. , Wang, S. , Chen, Y. , Ye, M. & Lu, L. (2013). Ceramide mediates ox‐LDL‐induced human vascular smooth muscle cell calcification via p38 mitogen‐activated protein kinase signaling. PLoS One 8(12), e82379. 24358176 10.1371/journal.pone.0082379 PMC3865066

[brv12866-bib-0112] Liao, P. , Yang, D. , Liu, D. & Zheng, Y. (2017). GLP‐1 and ghrelin attenuate high glucose/high lipid‐induced apoptosis and senescence of human microvascular endothelial cells. Cellular Physiology and Biochemistry 44(5), 1842–1855. 29224011 10.1159/000485820

[brv12866-bib-0113] Liao, L. , Zhuang, X. , Li, W. , Su, Q. , Zhao, J. & Liu, Y. (2018). Polysaccharide from Fuzi protects against OxLDLinduced calcification of human vascular smooth muscle cells by increasing autophagic activity. Molecular Medicine Reports 17(4), 5109–5115. 29393437 10.3892/mmr.2018.8488 PMC5865975

[brv12866-bib-0114] Libby, P. , Ridker, P. M. , Hansson, G. K. & Leducq Transatlantic Network on Atherothrombosis (2009). Inflammation in atherosclerosis: from pathophysiology to practice. Journal of the American College of Cardiology 54(23), 2129–2138. 19942084 10.1016/j.jacc.2009.09.009 PMC2834169

[brv12866-bib-0115] Lin, Y. , Chiba, S. , Suzuki, A. , Yamaguchi, S. , Nakanishi, T. , Matsumoto, H. , Ikeda, Y. , Ishibashi‐Ueda, H. , Hirano, K. & Kato, S. (2013). Vascular smooth muscle cells isolated from adipose triglyceride lipase‐deficient mice exhibit distinct phenotype and phenotypic plasticity. Biochemical and Biophysical Research Communications 434(3), 534–540. 23583398 10.1016/j.bbrc.2013.03.109

[brv12866-bib-0116] Lincoff, A. M. , Nicholls, S. J. , Riesmeyer, J. S. , Barter, P. J. , Brewer, H. B. , Fox, K. A. A. , Gibson, C. M. , Granger, C. , Menon, V. , Montalescot, G. , Rader, D. , Tall, A. R. , McErlean, E. , Wolski, K. , Ruotolo, G. , et al. (2017). Evacetrapib and cardiovascular outcomes in high‐risk vascular disease. The New England Journal of Medicine 376(20), 1933–1942. 28514624 10.1056/NEJMoa1609581

[brv12866-bib-0117] Lipskaia, L. , Pourci, M. L. , Delomenie, C. , Combettes, L. , Goudouneche, D. , Paul, J. L. , Capiod, T. & Lompre, A. M. (2003). Phosphatidylinositol 3‐kinase and calcium‐activated transcription pathways are required for VLDL‐induced smooth muscle cell proliferation. Circulation Research 92(10), 1115–1122. 12730091 10.1161/01.RES.0000074880.25540.D0

[brv12866-bib-0118] Liu, L. , Wen, T. , Zheng, X. Y. , Yang, D. G. , Zhao, S. P. , Xu, D. Y. & Lu, G. H. (2009a). Remnant‐like particles accelerate endothelial progenitor cells senescence and induce cellular dysfunction via an oxidative mechanism. Atherosclerosis 202(2), 405–414. 18582890 10.1016/j.atherosclerosis.2008.05.024

[brv12866-bib-0119] Liu, P. , Zhou, B. , Gu, D. , Zhang, L. & Han, Z. (2009b). Endothelial progenitor cell therapy in atherosclerosis: a double‐edged sword? Ageing Research Reviews 8(2), 83–93. 19103308 10.1016/j.arr.2008.11.002

[brv12866-bib-0120] Liu, Y. , Drozdov, I. , Shroff, R. , Beltran, L. E. & Shanahan, C. M. (2013). Prelamin A accelerates vascular calcification via activation of the DNA damage response and senescence‐associated secretory phenotype in vascular smooth muscle cells. Circulation Research 112(10), e99–e109. 23564641 10.1161/CIRCRESAHA.111.300543

[brv12866-bib-0121] Liu, W. Q. , Zhang, Y. Z. , Wu, Y. , Zhang, J. J. , Li, T. B. , Jiang, T. , Xiong, X. M. , Luo, X. J. , Ma, Q. L. & Peng, J. (2015). Myeloperoxidase‐derived hypochlorous acid promotes ox‐LDL‐induced senescence of endothelial cells through a mechanism involving beta‐catenin signaling in hyperlipidemia. Biochemical and Biophysical Research Communications 467(4), 859–865. 26474698 10.1016/j.bbrc.2015.10.053

[brv12866-bib-0122] Liu, X. , Tang, Y. , Cui, Y. , Zhang, H. & Zhang, D. (2016). Autophagy is associated with cell fate in the process of macrophage‐derived foam cells formation and progress. Journal of Biomedical Science 23(1), 57. 27473161 10.1186/s12929-016-0274-z PMC4967324

[brv12866-bib-0123] Liu, Z. , Wu, K. K. L. , Jiang, X. , Xu, A. & Cheng, K. K. Y. (2020). The role of adipose tissue senescence in obesity‐ and ageing‐related metabolic disorders. Clinical Science (London, England: 1979) 134(2), 315–330. 10.1042/CS20190966 31998947

[brv12866-bib-0124] Lopez‐Otin, C. , Blasco, M. A. , Partridge, L. , Serrano, M. & Kroemer, G. (2013). The hallmarks of aging. Cell 153(6), 1194–1217. 23746838 10.1016/j.cell.2013.05.039 PMC3836174

[brv12866-bib-0125] Lu, J. , Jiang, W. , Yang, J. H. , Chang, P. Y. , Walterscheid, J. P. , Chen, H. H. , Marcelli, M. , Tang, D. , Lee, Y. T. , Liao, W. S. , Yang, C. Y. & Chen, C. H. (2008). Electronegative LDL impairs vascular endothelial cell integrity in diabetes by disrupting fibroblast growth factor 2 (FGF2) autoregulation. Diabetes 57(1), 158–166. 17959932 10.2337/db07-1287

[brv12866-bib-0126] Lu, C. , Zhang, X. , Zhang, D. , Pei, E. , Xu, J. , Tang, T. , Ye, M. , Uzan, G. , Zhi, K. , Li, M. & Zuo, K. (2015). Short time tripterine treatment enhances endothelial progenitor cell function via heat shock protein 32. Journal of Cellular Physiology 230(5), 1139–1147. 25336054 10.1002/jcp.24849

[brv12866-bib-0127] Lugano, R. , Pena, E. , Casani, L. , Badimon, L. & Padro, T. (2013). UPA promotes lipid‐loaded vascular smooth muscle cell migration through LRP‐1. Cardiovascular Research 100(2), 262–271. 23812296 10.1093/cvr/cvt171

[brv12866-bib-0128] Luo, Z. , Xu, W. , Ma, S. , Qiao, H. , Gao, L. , Zhang, R. , Yang, B. , Qiu, Y. , Chen, J. , Zhang, M. , Tao, B. , Cao, F. & Wang, Y. (2017). Moderate autophagy inhibits vascular smooth muscle cell senescence to stabilize progressed atherosclerotic plaque via the mTORC1/ULK1/ATG13 signal pathway. Oxidative Medicine and Cellular Longevity 2017, 3018190. 28713484 10.1155/2017/3018190 PMC5497616

[brv12866-bib-0129] Ma, F. X. , Zhou, B. , Chen, Z. , Ren, Q. , Lu, S. H. , Sawamura, T. & Han, Z. C. (2006). Oxidized low density lipoprotein impairs endothelial progenitor cells by regulation of endothelial nitric oxide synthase. Journal of Lipid Research 47(6), 1227–1237. 16522925 10.1194/jlr.M500507-JLR200

[brv12866-bib-0130] Ma, K. L. , Ruan, X. Z. , Powis, S. H. , Moorhead, J. F. & Varghese, Z. (2007). Anti‐atherosclerotic effects of sirolimus on human vascular smooth muscle cells. American Journal of Physiology. Heart and Circulatory Physiology 292(6), H2721–H2728. 17322416 10.1152/ajpheart.01174.2006

[brv12866-bib-0131] Mahmoudi, M. , Gorenne, I. , Mercer, J. , Figg, N. , Littlewood, T. & Bennett, M. (2008). Statins use a novel Nijmegen breakage syndrome‐1‐dependent pathway to accelerate DNA repair in vascular smooth muscle cells. Circulation Research 103(7), 717–725. 18723444 10.1161/CIRCRESAHA.108.182899

[brv12866-bib-0132] Marin, C. , Delgado‐Lista, J. , Ramirez, R. , Carracedo, J. , Caballero, J. , Perez‐Martinez, P. , Gutierrez‐Mariscal, F. M. , Garcia‐Rios, A. , Delgado‐Casado, N. , Cruz‐Teno, C. , Yubero‐Serrano, E. M. , Tinahones, F. , Malagon Mdel, M. , Perez‐Jimenez, F. & Lopez‐Miranda, J. (2012). Mediterranean diet reduces senescence‐associated stress in endothelial cells. Age 34(6), 1309–1316. 21894446 10.1007/s11357-011-9305-6 PMC3528364

[brv12866-bib-0133] Marshall, S. M. (2017). 60 years of metformin use: a glance at the past and a look to the future. Diabetologia 60(9), 1561–1565. 28776085 10.1007/s00125-017-4343-y

[brv12866-bib-0134] Martel, J. , Ojcius, D. M. , Wu, C. Y. , Peng, H. H. , Voisin, L. , Perfettini, J. L. , Ko, Y. F. & Young, J. D. (2020). Emerging use of senolytics and senomorphics against aging and chronic diseases. Medicinal Research Reviews 40(6), 2114–2131. 32578904 10.1002/med.21702

[brv12866-bib-0135] Martinet, W. , Schrijvers, D. M. , Timmermans, J. P. & Bult, H. (2008). Interactions between cell death induced by statins and 7‐ketocholesterol in rabbit aorta smooth muscle cells. British Journal of Pharmacology 154(6), 1236–1246. 18469840 10.1038/bjp.2008.181 PMC2483392

[brv12866-bib-0136] Matthews, C. , Gorenne, I. , Scott, S. , Figg, N. , Kirkpatrick, P. , Ritchie, A. , Goddard, M. & Bennett, M. (2006). Vascular smooth muscle cells undergo telomere‐based senescence in human atherosclerosis: effects of telomerase and oxidative stress. Circulation Research 99(2), 156–164. 16794190 10.1161/01.RES.0000233315.38086.bc

[brv12866-bib-0137] Mattison, J. A. , Colman, R. J. , Beasley, T. M. , Allison, D. B. , Kemnitz, J. W. , Roth, G. S. , Ingram, D. K. , Weindruch, R. , de Cabo, R. & Anderson, R. M. (2017). Caloric restriction improves health and survival of rhesus monkeys. Nature Communications 8, 14063. 10.1038/ncomms14063 PMC5247583 28094793

[brv12866-bib-0138] McCay, C. M. , Maynard, L. A. , Sperling, G. & Barnes, L. L. (1975). The Journal of Nutrition. Volume 18 July–December, 1939. Pages 1–13. Retarded growth, life span, ultimate body size and age changes in the albino rat after feeding diets restricted in calories. Nutrition Reviews 33(8), 241–243. 1095975 10.1111/j.1753-4887.1975.tb05227.x

[brv12866-bib-0139] Michaud, S. E. , Dussault, S. , Haddad, P. , Groleau, J. & Rivard, A. (2006). Circulating endothelial progenitor cells from healthy smokers exhibit impaired functional activities. Atherosclerosis 187(2), 423–432. 16288934 10.1016/j.atherosclerosis.2005.10.009

[brv12866-bib-0140] Mills, K. F. , Yoshida, S. , Stein, L. R. , Grozio, A. , Kubota, S. , Sasaki, Y. , Redpath, P. , Migaud, M. E. , Apte, R. S. , Uchida, K. , Yoshino, J. & Imai, S. I. (2016). Long‐term administration of nicotinamide mononucleotide mitigates age‐associated physiological decline in mice. Cell Metabolism 24(6), 795–806. 28068222 10.1016/j.cmet.2016.09.013 PMC5668137

[brv12866-bib-0141] Minamino, T. , Miyauchi, H. , Yoshida, T. , Ishida, Y. , Yoshida, H. & Komuro, I. (2002). Endothelial cell senescence in human atherosclerosis: role of telomere in endothelial dysfunction. Circulation 105(13), 1541–1544. 11927518 10.1161/01.cir.0000013836.85741.17

[brv12866-bib-0142] Minamino, T. , Yoshida, T. , Tateno, K. , Miyauchi, H. , Zou, Y. , Toko, H. & Komuro, I. (2003). Ras induces vascular smooth muscle cell senescence and inflammation in human atherosclerosis. Circulation 108(18), 2264–2269. 14557365 10.1161/01.CIR.0000093274.82929.22

[brv12866-bib-0143] Minamino, T. , Orimo, M. , Shimizu, I. , Kunieda, T. , Yokoyama, M. , Ito, T. , Nojima, A. , Nabetani, A. , Oike, Y. , Matsubara, H. , Ishikawa, F. & Komuro, I. (2009). A crucial role for adipose tissue p53 in the regulation of insulin resistance. Nature Medicine 15(9), 1082–1087. 10.1038/nm.2014 19718037

[brv12866-bib-0144] Ming, G. F. , Tang, Y. J. , Hu, K. , Chen, Y. , Huang, W. H. & Xiao, J. (2016). Visfatin attenuates the ox‐LDL‐induced senescence of endothelial progenitor cells by upregulating SIRT1 expression through the PI3K/Akt/ERK pathway. International Journal of Molecular Medicine 38(2), 643–649. 27277186 10.3892/ijmm.2016.2633

[brv12866-bib-0145] Mishra, A. , Chaudhary, A. & Sethi, S. (2004). Oxidized omega‐3 fatty acids inhibit NF‐kappaB activation via a PPARalpha‐dependent pathway. Arteriosclerosis, Thrombosis, and Vascular Biology 24(9), 1621–1627. 15231516 10.1161/01.ATV.0000137191.02577.86

[brv12866-bib-0146] Morgantini, C. , Natali, A. , Boldrini, B. , Imaizumi, S. , Navab, M. , Fogelman, A. M. , Ferrannini, E. & Reddy, S. T. (2011). Anti‐inflammatory and antioxidant properties of HDLs are impaired in type 2 diabetes. Diabetes 60(10), 2617–2623. 21852676 10.2337/db11-0378 PMC3178289

[brv12866-bib-0147] Muller, K. , Dulku, S. , Hardwick, S. J. , Skepper, J. N. & Mitchinson, M. J. (2001). Changes in vimentin in human macrophages during apoptosis induced by oxidised low density lipoprotein. Atherosclerosis 156(1), 133–144. 11369006 10.1016/s0021-9150(00)00641-9

[brv12866-bib-0148] Nakamura, K. , Miura, D. , Saito, Y. , Yunoki, K. , Koyama, Y. , Satoh, M. , Kondo, M. , Osawa, K. , Hatipoglu, O. F. , Miyoshi, T. , Yoshida, M. , Morita, H. & Ito, H. (2017). Eicosapentaenoic acid prevents arterial calcification in klotho mutant mice. PLoS One 12(8), e0181009. 28771600 10.1371/journal.pone.0181009 PMC5542469

[brv12866-bib-0149] Nakano‐Kurimoto, R. , Ikeda, K. , Uraoka, M. , Nakagawa, Y. , Yutaka, K. , Koide, M. , Takahashi, T. , Matoba, S. , Yamada, H. , Okigaki, M. & Matsubara, H. (2009). Replicative senescence of vascular smooth muscle cells enhances the calcification through initiating the osteoblastic transition. American Journal of Physiology. Heart and Circulatory Physiology 297(5), H1673–H1684. 19749165 10.1152/ajpheart.00455.2009

[brv12866-bib-0150] Nguyen, H. P. , Lin, F. , Yi, D. , Xie, Y. , Dinh, J. , Xue, P. & Sul, H. S. (2021). Aging‐dependent regulatory cells emerge in subcutaneous fat to inhibit adipogenesis. Developmental Cell 56(10), 1437–1451.e3. 33878347 10.1016/j.devcel.2021.03.026 PMC8137669

[brv12866-bib-0151] Ni, Y. Q. , Lin, X. , Zhan, J. K. & Liu, Y. S. (2020). Roles and functions of exosomal non‐coding RNAs in vascular aging. Aging and Disease 11(1), 164–178. 32010490 10.14336/AD.2019.0402 PMC6961769

[brv12866-bib-0152] Noor, R. , Shuaib, U. , Wang, C. X. , Todd, K. , Ghani, U. , Schwindt, B. & Shuaib, A. (2007). High‐density lipoprotein cholesterol regulates endothelial progenitor cells by increasing eNOS and preventing apoptosis. Atherosclerosis 192(1), 92–99. 16884727 10.1016/j.atherosclerosis.2006.06.023

[brv12866-bib-0153] Norata, G. D. , Grigore, L. , Raselli, S. , Seccomandi, P. M. , Hamsten, A. , Maggi, F. M. , Eriksson, P. & Catapano, A. L. (2006). Triglyceride‐rich lipoproteins from hypertriglyceridemic subjects induce a pro‐inflammatory response in the endothelium: molecular mechanisms and gene expression studies. Journal of Molecular and Cellular Cardiology 40(4), 484–494. 16516917 10.1016/j.yjmcc.2006.01.022

[brv12866-bib-0154] North, B. J. & Sinclair, D. A. (2012). The intersection between aging and cardiovascular disease. Circulation Research 110(8), 1097–1108. 22499900 10.1161/CIRCRESAHA.111.246876 PMC3366686

[brv12866-bib-0155] Oesterle, A. , Laufs, U. & Liao, J. K. (2017). Pleiotropic effects of statins on the cardiovascular system. Circulation Research 120(1), 229–243. 28057795 10.1161/CIRCRESAHA.116.308537 PMC5467317

[brv12866-bib-0156] Oh, S. T. , Park, H. , Yoon, H. J. & Yang, S. Y. (2017). Long‐term treatment of native LDL induces senescence of cultured human endothelial cells. Oxidative Medicine and Cellular Longevity 2017, 6487825. 28197300 10.1155/2017/6487825 PMC5288541

[brv12866-bib-0157] Ohman, M. K. , Wright, A. P. , Wickenheiser, K. J. , Luo, W. , Russo, H. M. & Eitzman, D. T. (2010). Monocyte chemoattractant protein‐1 deficiency protects against visceral fat‐induced atherosclerosis. Arteriosclerosis, Thrombosis, and Vascular Biology 30(6), 1151–1158. 20299683 10.1161/ATVBAHA.110.205914 PMC2874123

[brv12866-bib-0158] Ok, E. , Basnakian, A. G. , Apostolov, E. O. , Barri, Y. M. & Shah, S. V. (2005). Carbamylated low‐density lipoprotein induces death of endothelial cells: a link to atherosclerosis in patients with kidney disease. Kidney International 68(1), 173–178. 15954906 10.1111/j.1523-1755.2005.00391.x

[brv12866-bib-0159] Onken, B. & Driscoll, M. (2010). Metformin induces a dietary restriction‐like state and the oxidative stress response to extend *C. elegans* Healthspan via AMPK, LKB1, and SKN‐1. PLoS One 5(1), e8758. 20090912 10.1371/journal.pone.0008758 PMC2807458

[brv12866-bib-0160] Osonoi, Y. , Mita, T. , Azuma, K. , Nakajima, K. , Masuyama, A. , Goto, H. , Nishida, Y. , Miyatsuka, T. , Fujitani, Y. , Koike, M. , Mitsumata, M. & Watada, H. (2018). Defective autophagy in vascular smooth muscle cells enhances cell death and atherosclerosis. Autophagy 14(11), 1991–2006. 30025494 10.1080/15548627.2018.1501132 PMC6152523

[brv12866-bib-0161] Ota, H. , Eto, M. , Kano, M. R. , Kahyo, T. , Setou, M. , Ogawa, S. , Iijima, K. , Akishita, M. & Ouchi, Y. (2010). Induction of endothelial nitric oxide synthase, SIRT1, and catalase by statins inhibits endothelial senescence through the Akt pathway. Arteriosclerosis, Thrombosis, and Vascular Biology 30(11), 2205–2211. 20705918 10.1161/ATVBAHA.110.210500

[brv12866-bib-0162] Pacheco, Y. M. , Abia, R. , Perona, J. S. , Reina, M. , Ruiz‐Gutierrez, V. , Montero, E. & Muriana, F. J. (2001). Triacylglycerol‐rich lipoproteins interact with human vascular cells in a lipid‐dependent fashion. Journal of Agricultural and Food Chemistry 49(11), 5653–5661. 11714373 10.1021/jf010576n

[brv12866-bib-0163] Palmer, A. K. , Xu, M. , Zhu, Y. , Pirtskhalava, T. , Weivoda, M. M. , Hachfeld, C. M. , Prata, L. G. , van Dijk, T. H. , Verkade, E. , Casaclang‐Verzosa, G. , Johnson, K. O. , Cubro, H. , Doornebal, E. J. , Ogrodnik, M. , Jurk, D. , et al. (2019). Targeting senescent cells alleviates obesity‐induced metabolic dysfunction. Aging Cell 18(3), e12950. 30907060 10.1111/acel.12950 PMC6516193

[brv12866-bib-0164] Pan, X. X. , Ruan, C. C. , Liu, X. Y. , Kong, L. R. , Ma, Y. , Wu, Q. H. , Li, H. Q. , Sun, Y. J. , Chen, A. Q. , Zhao, Q. , Wu, F. , Wang, X. J. , Wang, J. G. , Zhu, D. L. & Gao, P. J. (2019). Perivascular adipose tissue‐derived stromal cells contribute to vascular remodeling during aging. Aging Cell 18(4), e12969. 31087498 10.1111/acel.12969 PMC6612678

[brv12866-bib-0165] Park, K. H. , Jang, W. , Kim, K. Y. , Kim, J. R. & Cho, K. H. (2010). Fructated apolipoprotein A‐I showed severe structural modification and loss of beneficial functions in lipid‐free and lipid‐bound state with acceleration of atherosclerosis and senescence. Biochemical and Biophysical Research Communications 392(3), 295–300. 20059975 10.1016/j.bbrc.2009.12.179

[brv12866-bib-0166] Park, K. H. , Kim, J. Y. , Choi, I. , Kim, J. R. , Won, K. C. & Cho, K. H. (2016). Fructated apolipoprotein A‐I exacerbates cellular senescence in human umbilical vein endothelial cells accompanied by impaired insulin secretion activity and embryo toxicity. Biochemistry and Cell Biology 94(4), 337–345. 27487295 10.1139/bcb-2015-0165

[brv12866-bib-0167] Partridge, L. , Fuentealba, M. & Kennedy, B. K. (2020). The quest to slow ageing through drug discovery. Nature Reviews Drug Discovery 19(8), 513–532. 32467649 10.1038/s41573-020-0067-7

[brv12866-bib-0168] Parvizi, M. , Ryan, Z. C. , Ebtehaj, S. , Arendt, B. K. & Lanza, I. R. (2021). The secretome of senescent preadipocytes influences the phenotype and function of cells of the vascular wall. Biochimica et Biophysica Acta. Molecular Basis of Disease 1867(1), 165983. 33002577 10.1016/j.bbadis.2020.165983

[brv12866-bib-0169] Patel, R. , Varghese, J. F. , Singh, R. P. & Yadav, U. C. S. (2019). Induction of endothelial dysfunction by oxidized low‐density lipoproteins via downregulation of Erk‐5/Mef2c/KLF2 signaling: amelioration by fisetin. Biochimie 163, 152–162. 31199942 10.1016/j.biochi.2019.06.007

[brv12866-bib-0170] Peiffer, V. , Sherwin, S. J. & Weinberg, P. D. (2013). Does low and oscillatory wall shear stress correlate spatially with early atherosclerosis? A systematic review. Cardiovascular Research 99(2), 242–250. 23459102 10.1093/cvr/cvt044 PMC3695746

[brv12866-bib-0171] Persson, J. , Nilsson, J. & Lindholm, M. W. (2006). Cytokine response to lipoprotein lipid loading in human monocyte‐derived macrophages. Lipids in Health and Disease 5, 17. 16800873 10.1186/1476-511X-5-17 PMC1524960

[brv12866-bib-0172] de Picciotto, N. E. , Gano, L. B. , Johnson, L. C. , Martens, C. R. , Sindler, A. L. , Mills, K. F. , Imai, S. & Seals, D. R. (2016). Nicotinamide mononucleotide supplementation reverses vascular dysfunction and oxidative stress with aging in mice. Aging Cell 15(3), 522–530. 26970090 10.1111/acel.12461 PMC4854911

[brv12866-bib-0173] Pifferi, F. , Terrien, J. , Marchal, J. , Dal‐Pan, A. , Djelti, F. , Hardy, I. , Chahory, S. , Cordonnier, N. , Desquilbet, L. , Hurion, M. , Zahariev, A. , Chery, I. , Zizzari, P. , Perret, M. , Epelbaum, J. , et al. (2018). Caloric restriction increases lifespan but affects brain integrity in grey mouse lemur primates. Communications Biology 1, 30. 30271916 10.1038/s42003-018-0024-8 PMC6123706

[brv12866-bib-0174] Pu, D. R. & Liu, L. (2007). Remnant like particles may induce atherosclerosis via accelerating endothelial progenitor cells senescence. Medical Hypotheses 69(2), 293–296. 17306468 10.1016/j.mehy.2006.11.046

[brv12866-bib-0175] Pu, D. R. & Liu, L. (2008). HDL slowing down endothelial progenitor cells senescence: a novel anti‐atherogenic property of HDL. Medical Hypotheses 70(2), 338–342. 17640824 10.1016/j.mehy.2007.05.025

[brv12866-bib-0176] Qi, X. Y. , Qu, S. L. , Xiong, W. H. , Rom, O. , Chang, L. & Jiang, Z. S. (2018). Perivascular adipose tissue (PVAT) in atherosclerosis: a double‐edged sword. Cardiovascular Diabetology 17(1), 134. 30305178 10.1186/s12933-018-0777-x PMC6180425

[brv12866-bib-0177] Qian, W. , Cai, X. , Qian, Q. , Zhuang, Q. , Yang, W. , Zhang, X. & Zhao, L. (2019). Astragaloside IV protects endothelial progenitor cells from the damage of ox‐LDL via the LOX‐1/NLRP3 inflammasome pathway. Drug Design. Development and Therapy 13, 2579–2589. 31440038 10.2147/DDDT.S207774 PMC6677131

[brv12866-bib-0178] Quesada, I. , Cejas, J. , Garcia, R. , Cannizzo, B. , Redondo, A. & Castro, C. (2018). Vascular dysfunction elicited by a cross talk between periaortic adipose tissue and the vascular wall is reversed by pioglitazone. Cardiovascular Therapeutics 36(3), e12322. 29464937 10.1111/1755-5922.12322

[brv12866-bib-0179] Quijano, C. , Cao, L. , Fergusson, M. M. , Romero, H. , Liu, J. , Gutkind, S. , Rovira, I. I. , Mohney, R. P. , Karoly, E. D. & Finkel, T. (2012). Oncogene‐induced senescence results in marked metabolic and bioenergetic alterations. Cell Cycle 11(7), 1383–1392. 22421146 10.4161/cc.19800 PMC3350879

[brv12866-bib-0180] Qureshi, A. W. , Altamimy, R. , El Habhab, A. , El Itawi, H. , Farooq, M. A. , Zobairi, F. , Hasan, H. , Amoura, L. , Kassem, M. , Auger, C. , Schini‐Kerth, V. & Toti, F. (2020). Ageing enhances the shedding of splenocyte microvesicles with endothelial pro‐senescent effect that is prevented by a short‐term intake of omega‐3 PUFA EPA:DHA 6:1. Biochemical Pharmacology 173, 113734. 31811867 10.1016/j.bcp.2019.113734

[brv12866-bib-0181] Rajamani, A. , Borkowski, K. , Akre, S. , Fernandez, A. , Newman, J. W. , Simon, S. I. & Passerini, A. G. (2019). Oxylipins in triglyceride‐rich lipoproteins of dyslipidemic subjects promote endothelial inflammation following a high fat meal. Scientific Reports 9(1), 8655. 31209255 10.1038/s41598-019-45005-5 PMC6572825

[brv12866-bib-0182] Rauscher, F. M. , Goldschmidt‐Clermont, P. J. , Davis, B. H. , Wang, T. , Gregg, D. , Ramaswami, P. , Pippen, A. M. , Annex, B. H. , Dong, C. & Taylor, D. A. (2003). Aging, progenitor cell exhaustion, and atherosclerosis. Circulation 108(4), 457–463. 12860902 10.1161/01.CIR.0000082924.75945.48

[brv12866-bib-0183] Ridker, P. M. , Everett, B. M. , Thuren, T. , MacFadyen, J. G. , Chang, W. H. , Ballantyne, C. , Fonseca, F. , Nicolau, J. , Koenig, W. , Anker, S. D. , Kastelein, J. J. P. , Cornel, J. H. , Pais, P. , Pella, D. , Genest, J. , et al. (2017). Antiinflammatory therapy with canakinumab for atherosclerotic disease. The New England Journal of Medicine 377(12), 1119–1131. 28845751 10.1056/NEJMoa1707914

[brv12866-bib-0184] Ridker, P. M. , Everett, B. M. , Pradhan, A. , MacFadyen, J. G. , Solomon, D. H. , Zaharris, E. , Mam, V. , Hasan, A. , Rosenberg, Y. , Iturriaga, E. , Gupta, M. , Tsigoulis, M. , Verma, S. , Clearfield, M. , Libby, P. , et al. (2019). Low‐dose methotrexate for the prevention of atherosclerotic events. The New England Jounal of Medicine 380(8), 752–762. 10.1056/NEJMoa1809798 PMC6587584 30415610

[brv12866-bib-0185] Roldan, M. , Macias‐Gonzalez, M. , Garcia, R. , Tinahones, F. J. & Martin, M. (2011). Obesity short‐circuits stemness gene network in human adipose multipotent stem cells. FASEB Journal 25(12), 4111–4126. 21846837 10.1096/fj.10-171439

[brv12866-bib-0186] Roos, C. M. , Zhang, B. , Palmer, A. K. , Ogrodnik, M. B. , Pirtskhalava, T. , Thalji, N. M. , Hagler, M. , Jurk, D. , Smith, L. A. , Casaclang‐Verzosa, G. , Zhu, Y. , Schafer, M. J. , Tchkonia, T. , Kirkland, J. L. & Miller, J. D. (2016). Chronic senolytic treatment alleviates established vasomotor dysfunction in aged or atherosclerotic mice. Aging Cell 15(5), 973–977. 26864908 10.1111/acel.12458 PMC5013022

[brv12866-bib-0187] Rosenson, R. S. , Brewer, H. B. Jr. , Ansell, B. J. , Barter, P. , Chapman, M. J. , Heinecke, J. W. , Kontush, A. , Tall, A. R. & Webb, N. R. (2016). Dysfunctional HDL and atherosclerotic cardiovascular disease. Nature Reviews Cardiology 13(1), 48–60. 26323267 10.1038/nrcardio.2015.124 PMC6245940

[brv12866-bib-0188] Saito, Y. , Nakamura, K. , Miura, D. , Yunoki, K. , Miyoshi, T. , Yoshida, M. , Kawakita, N. , Kimura, T. , Kondo, M. , Sarashina, T. , Akagi, S. , Watanabe, A. , Nishii, N. , Morita, H. & Ito, H. (2017). Suppression of Wnt signaling and osteogenic changes in vascular smooth muscle cells by eicosapentaenoic acid. Nutrients 9(8), 858. 10.3390/nu9080858 PMC5579651 28796175

[brv12866-bib-0189] Sakai, C. , Ishida, M. , Ohba, H. , Yamashita, H. , Uchida, H. , Yoshizumi, M. & Ishida, T. (2017). Fish oil omega‐3 polyunsaturated fatty acids attenuate oxidative stress‐induced DNA damage in vascular endothelial cells. PLoS One 12(11), e0187934. 29121093 10.1371/journal.pone.0187934 PMC5679535

[brv12866-bib-0190] Salabei, J. K. & Hill, B. G. (2013). Implications of autophagy for vascular smooth muscle cell function and plasticity. Free Radical Biology & Medicine 65, 693–703. 23938401 10.1016/j.freeradbiomed.2013.08.003 PMC3859773

[brv12866-bib-0191] Savji, N. , Rockman, C. B. , Skolnick, A. H. , Guo, Y. , Adelman, M. A. , Riles, T. & Berger, J. S. (2013). Association between advanced age and vascular disease in different arterial territories: a population database of over 3.6 million subjects. Journal of the American College of Cardiology 61(16), 1736–1743. 23500290 10.1016/j.jacc.2013.01.054

[brv12866-bib-0192] Schmidt‐Lucke, C. , Rossig, L. , Fichtlscherer, S. , Vasa, M. , Britten, M. , Kamper, U. , Dimmeler, S. & Zeiher, A. M. (2005). Reduced number of circulating endothelial progenitor cells predicts future cardiovascular events: proof of concept for the clinical importance of endogenous vascular repair. Circulation 111(22), 2981–2987. 15927972 10.1161/CIRCULATIONAHA.104.504340

[brv12866-bib-0193] Schutz, E. , Gogiraju, R. , Pavlaki, M. , Drosos, I. , Georgiadis, G. S. , Argyriou, C. , Rim Ben Hallou, A. , Konstantinou, F. , Mikroulis, D. , Schuler, R. , Bochenek, M. L. , Gachkar, S. , Buschmann, K. , Lankeit, M. , Karbach, S. H. , et al. (2019). Age‐dependent and ‐independent effects of perivascular adipose tissue and its paracrine activities during neointima formation. International Journal of Molecular Sciences 21(1), 282. 10.3390/ijms21010282 PMC6981748 31906225

[brv12866-bib-0194] Schwartz, S. M. & Benditt, E. P. (1977). Aortic endothelial cell replication. I. Effects of age and hypertension in the rat. Circulation Research 41(2), 248–255. 872300 10.1161/01.res.41.2.248

[brv12866-bib-0195] Sever, P. S. , Dahlof, B. , Poulter, N. R. , Wedel, H. , Beevers, G. , Caulfield, M. , Collins, R. , Kjeldsen, S. E. , Kristinsson, A. , McInnes, G. T. , Mehlsen, J. , Nieminen, M. , O'Brien, E. , Ostergren, J. & ASCOT Investigators (2003). Prevention of coronary and stroke events with atorvastatin in hypertensive patients who have average or lower‐than‐average cholesterol concentrations, in the Anglo‐Scandinavian Cardiac Outcomes Trial–Lipid Lowering Arm (ASCOT‐LLA): a multicentre randomised controlled trial. The Lancet 361(9364), 1149–1158. 10.1016/S0140-6736(03)12948-0 12686036

[brv12866-bib-0196] Shi, Q. , Hubbard, G. B. , Kushwaha, R. S. , Rainwater, D. , Thomas, C. A. III , Leland, M. M. , Vandeberg, J. L. & Wang, X. L. (2007). Endothelial senescence after high‐cholesterol, high‐fat diet challenge in baboons. American Journal of Physiology. Heart and Circulatory Physiology 292(6), H2913–H2920. 17277030 10.1152/ajpheart.01405.2006

[brv12866-bib-0197] Shi, G. , Liu, D. , Zhou, B. , Liu, Y. , Hao, B. , Yu, S. , Wu, L. , Wang, M. , Song, Z. , Wu, C. , Zhu, J. & Qian, X. (2020). Ginsenoside Rb1 alleviates oxidative low‐density lipoprotein‐induced vascular endothelium senescence via the SIRT1/Beclin‐1/autophagy axis. Journal of Cardiovascular Pharmacology 75(2), 155–167. 31658172 10.1097/FJC.0000000000000775

[brv12866-bib-0198] Shih, C. M. , Lin, F. Y. , Yeh, J. S. , Lin, Y. W. , Loh, S. H. , Tsao, N. W. , Nakagami, H. , Morishita, R. , Sawamura, T. , Li, C. Y. , Lin, C. Y. & Huang, C. Y. (2019). Dysfunctional high density lipoprotein failed to rescue the function of oxidized low density lipoprotein‐treated endothelial progenitor cells: a novel index for the prediction of HDL functionality. Translational Research 205, 17–32. 30720435 10.1016/j.trsl.2018.09.005

[brv12866-bib-0199] Shin, H. K. , Kim, Y. K. , Kim, K. Y. , Lee, J. H. & Hong, K. W. (2004). Remnant lipoprotein particles induce apoptosis in endothelial cells by NAD(P)H oxidase‐mediated production of superoxide and cytokines via lectin‐like oxidized low‐density lipoprotein receptor‐1 activation: prevention by cilostazol. Circulation 109(8), 1022–1028. 14967724 10.1161/01.CIR.0000117403.64398.53

[brv12866-bib-0200] Silva, G. C. , Abbas, M. , Khemais‐Benkhiat, S. , Burban, M. , Ribeiro, T. P. , Toti, F. , Idris‐Khodja, N. , Cortes, S. F. & Schini‐Kerth, V. B. (2017). Replicative senescence promotes prothrombotic responses in endothelial cells: role of NADPH oxidase‐ and cyclooxygenase‐derived oxidative stress. Experimental Gerontology 93, 7–15. 28412252 10.1016/j.exger.2017.04.006

[brv12866-bib-0201] Smirnova, E. , Goldberg, E. B. , Makarova, K. S. , Lin, L. , Brown, W. J. & Jackson, C. L. (2006). ATGL has a key role in lipid droplet/adiposome degradation in mammalian cells. EMBO Reports 7(1), 106–113. 16239926 10.1038/sj.embor.7400559 PMC1369222

[brv12866-bib-0202] Son, M. , Oh, S. , Jang, J. T. , Park, C. H. , Son, K. H. & Byun, K. (2020). Attenuating effects of pyrogallol‐phloroglucinol‐6,6‐bieckol on vascular smooth muscle cell phenotype changes to osteoblastic cells and vascular calcification induced by high fat diet. Nutrients 12(9), 2777. 10.3390/nu12092777 PMC7551448 32932908

[brv12866-bib-0203] Song, X. , Yang, B. , Qiu, F. , Jia, M. & Fu, G. (2017a). High glucose and free fatty acids induce endothelial progenitor cell senescence via PGC‐1alpha/SIRT1 signaling pathway. Cell Biology International 41(10), 1146–1159. 28786152 10.1002/cbin.10833

[brv12866-bib-0204] Song, Y. , Hou, M. , Li, Z. , Luo, C. , Ou, J. S. , Yu, H. , Yan, J. & Lu, L. (2017b). TLR4/NF‐kappaB/ceramide signaling contributes to ox‐LDL‐induced calcification of human vascular smooth muscle cells. European Journal of Pharmacology 794, 45–51. 27876618 10.1016/j.ejphar.2016.11.029

[brv12866-bib-0205] Soria‐Florido, M. T. , Castaner, O. , Lassale, C. , Estruch, R. , Salas‐Salvado, J. , Martinez‐Gonzalez, M. A. , Corella, D. , Ros, E. , Aros, F. , Elosua, R. , Lapetra, J. , Fiol, M. , Alonso‐Gomez, A. , Gomez‐Gracia, E. , Serra‐Majem, L. , et al. (2020). Dysfunctional high‐density lipoproteins are associated with a greater incidence of acute coronary syndrome in a population at high cardiovascular risk: a nested case‐control study. Circulation 141(6), 444–453. 31941372 10.1161/CIRCULATIONAHA.119.041658

[brv12866-bib-0206] Steffensen, L. B. , Mortensen, M. B. , Kjolby, M. , Hagensen, M. K. , Oxvig, C. & Bentzon, J. F. (2015). Disturbed laminar blood flow vastly augments lipoprotein retention in the artery wall: a key mechanism distinguishing susceptible from resistant sites. Arteriosclerosis, Thrombosis, and Vascular Biology 35(9), 1928–1935. 26183617 10.1161/ATVBAHA.115.305874

[brv12866-bib-0207] Steinberg, D. , Parthasarathy, S. , Carew, T. E. , Khoo, J. C. & Witztum, J. L. (1989). Beyond cholesterol. Modifications of low‐density lipoprotein that increase its atherogenicity. The New England Journal of Medicine 320(14), 915–924. 2648148 10.1056/NEJM198904063201407

[brv12866-bib-0208] Stojanovic, S. D. , Fiedler, J. , Bauersachs, J. , Thum, T. & Sedding, D. G. (2020). Senescence‐induced inflammation: an important player and key therapeutic target in atherosclerosis. European Heart Journal 41(31), 2983–2996. 31898722 10.1093/eurheartj/ehz919 PMC7453834

[brv12866-bib-0209] Stollenwerk, M. M. , Lindholm, M. W. , Porn‐Ares, M. I. , Larsson, A. , Nilsson, J. & Ares, M. P. (2005a). Very low‐density lipoprotein induces interleukin‐1beta expression in macrophages. Biochemical and Biophysical Research Communications 335(2), 603–608. 16087165 10.1016/j.bbrc.2005.07.123

[brv12866-bib-0210] Stollenwerk, M. M. , Schiopu, A. , Fredrikson, G. N. , Dichtl, W. , Nilsson, J. & Ares, M. P. (2005b). Very low density lipoprotein potentiates tumor necrosis factor‐alpha expression in macrophages. Atherosclerosis 179(2), 247–254. 15777538 10.1016/j.atherosclerosis.2004.12.002

[brv12866-bib-0211] Sumi, M. , Sata, M. , Miura, S. , Rye, K. A. , Toya, N. , Kanaoka, Y. , Yanaga, K. , Ohki, T. , Saku, K. & Nagai, R. (2007). Reconstituted high‐density lipoprotein stimulates differentiation of endothelial progenitor cells and enhances ischemia‐induced angiogenesis. Arteriosclerosis, Thrombosis, and Vascular Biology 27(4), 813–818. 17272742 10.1161/01.ATV.0000259299.38843.64

[brv12866-bib-0212] Sun, H. , Unoki, H. , Wang, X. , Liang, J. , Ichikawa, T. , Arai, Y. , Shiomi, M. , Marcovina, S. M. , Watanabe, T. & Fan, J. (2002). Lipoprotein(a) enhances advanced atherosclerosis and vascular calcification in WHHL transgenic rabbits expressing human apolipoprotein(a). The Journal of Biological Chemistry 277(49), 47486–47492. 12196525 10.1074/jbc.M205814200

[brv12866-bib-0213] Tai, M. H. , Kuo, S. M. , Liang, H. T. , Chiou, K. R. , Lam, H. C. , Hsu, C. M. , Pownall, H. J. , Chen, H. H. , Huang, M. T. & Yang, C. Y. (2006). Modulation of angiogenic processes in cultured endothelial cells by low density lipoproteins subfractions from patients with familial hypercholesterolemia. Atherosclerosis 186(2), 448–457. 16185697 10.1016/j.atherosclerosis.2005.08.022

[brv12866-bib-0214] Takahashi, S. (2017). Triglyceride rich lipoprotein ‐LPL‐VLDL receptor and Lp(a)‐VLDL receptor pathways for macrophage foam cell formation. Journal of Atherosclerosis and Thrombosis 24(6), 552–559. 28428482 10.5551/jat.RV17004 PMC5453679

[brv12866-bib-0215] Takahashi, Y. , Fujioka, Y. , Takahashi, T. , Domoto, K. , Takahashi, A. , Taniguchi, T. , Ishikawa, Y. & Yokoyama, M. (2005). Chylomicron remnants regulate early growth response factor‐1 in vascular smooth muscle cells. Life Sciences 77(6), 670–682. 15921998 10.1016/j.lfs.2005.01.012

[brv12866-bib-0216] Tang, D. , Lu, J. , Walterscheid, J. P. , Chen, H. H. , Engler, D. A. , Sawamura, T. , Chang, P. Y. , Safi, H. J. , Yang, C. Y. & Chen, C. H. (2008). Electronegative LDL circulating in smokers impairs endothelial progenitor cell differentiation by inhibiting Akt phosphorylation via LOX‐1. Journal of Lipid Research 49(1), 33–47. 17909223 10.1194/jlr.M700305-JLR200

[brv12866-bib-0217] Tang, Y. , Xu, Q. , Peng, H. , Liu, Z. , Yang, T. , Yu, Z. , Cheng, G. , Li, X. , Zhang, G. & Shi, R. (2015). The role of vascular peroxidase 1 in ox‐LDL‐induced vascular smooth muscle cell calcification. Atherosclerosis 243(2), 357–363. 26520887 10.1016/j.atherosclerosis.2015.08.047

[brv12866-bib-0218] Tchkonia, T. , Tchoukalova, Y. D. , Giorgadze, N. , Pirtskhalava, T. , Karagiannides, I. , Forse, R. A. , Koo, A. , Stevenson, M. , Chinnappan, D. , Cartwright, A. , Jensen, M. D. & Kirkland, J. L. (2005). Abundance of two human preadipocyte subtypes with distinct capacities for replication, adipogenesis, and apoptosis varies among fat depots. American Journal of Physiology. Endocrinology and Metabolism 288(1), E267–E277. 15383371 10.1152/ajpendo.00265.2004

[brv12866-bib-0219] Tchkonia, T. , Morbeck, D. E. , Von Zglinicki, T. , Van Deursen, J. , Lustgarten, J. , Scrable, H. , Khosla, S. , Jensen, M. D. & Kirkland, J. L. (2010). Fat tissue, aging, and cellular senescence. Aging Cell 9(5), 667–684. 20701600 10.1111/j.1474-9726.2010.00608.x PMC2941545

[brv12866-bib-0220] Thum, T. , Hoeber, S. , Froese, S. , Klink, I. , Stichtenoth, D. O. , Galuppo, P. , Jakob, M. , Tsikas, D. , Anker, S. D. , Poole‐Wilson, P. A. , Borlak, J. , Ertl, G. & Bauersachs, J. (2007). Age‐dependent impairment of endothelial progenitor cells is corrected by growth‐hormone‐mediated increase of insulin‐like growth‐factor‐1. Circulation Research 100(3), 434–443. 17234973 10.1161/01.RES.0000257912.78915.af

[brv12866-bib-0221] Tian, F. , Xiang, Q. Y. , Zhang, M. Y. , Chen, Y. Q. , Lin, Q. Z. , Wen, T. & Liu, L. (2019). Changes in non‐fasting concentrations of blood lipids after a daily Chinese breakfast in overweight subjects without fasting hypertriglyceridemia. Clinica Chimica Acta 490, 147–153. 10.1016/j.cca.2019.01.004 30615853

[brv12866-bib-0222] Ting, H. J. , Stice, J. P. , Schaff, U. Y. , Hui, D. Y. , Rutledge, J. C. , Knowlton, A. A. , Passerini, A. G. & Simon, S. I. (2007). Triglyceride‐rich lipoproteins prime aortic endothelium for an enhanced inflammatory response to tumor necrosis factor‐alpha. Circulation Research 100(3), 381–390. 17234968 10.1161/01.RES.0000258023.76515.a3

[brv12866-bib-0223] Torisu, K. , Singh, K. K. , Torisu, T. , Lovren, F. , Liu, J. , Pan, Y. , Quan, A. , Ramadan, A. , Al‐Omran, M. , Pankova, N. , Boyd, S. R. , Verma, S. & Finkel, T. (2016). Intact endothelial autophagy is required to maintain vascular lipid homeostasis. Aging Cell 15(1), 187–191. 26780888 10.1111/acel.12423 PMC4717267

[brv12866-bib-0224] Tso, C. , Martinic, G. , Fan, W. H. , Rogers, C. , Rye, K. A. & Barter, P. J. (2006). High‐density lipoproteins enhance progenitor‐mediated endothelium repair in mice. Arteriosclerosis, Thrombosis, and Vascular Biology 26(5), 1144–1149. 16528007 10.1161/01.ATV.0000216600.37436.cf

[brv12866-bib-0225] Tyrrell, D. J. , Blin, M. G. , Song, J. , Wood, S. C. , Zhang, M. , Beard, D. A. & Goldstein, D. R. (2020). Age‐associated mitochondrial dysfunction accelerates atherogenesis. Circulation Research 126(3), 298–314. 31818196 10.1161/CIRCRESAHA.119.315644 PMC7006722

[brv12866-bib-0226] Ungvari, Z. , Podlutsky, A. , Sosnowska, D. , Tucsek, Z. , Toth, P. , Deak, F. , Gautam, T. , Csiszar, A. & Sonntag, W. E. (2013). Ionizing radiation promotes the acquisition of a senescence‐associated secretory phenotype and impairs angiogenic capacity in cerebromicrovascular endothelial cells: role of increased DNA damage and decreased DNA repair capacity in microvascular radiosensitivity. The Journals of Gerontology. Series A, Biological Sciences and Medical Sciences 68(12), 1443–1457. 23689827 10.1093/gerona/glt057 PMC3814240

[brv12866-bib-0227] Van Harmelen, V. , Rohrig, K. & Hauner, H. (2004). Comparison of proliferation and differentiation capacity of human adipocyte precursor cells from the omental and subcutaneous adipose tissue depot of obese subjects. Metabolism 53(5), 632–637. 15131769 10.1016/j.metabol.2003.11.012

[brv12866-bib-0228] Vasa, M. , Fichtlscherer, S. , Aicher, A. , Adler, K. , Urbich, C. , Martin, H. , Zeiher, A. M. & Dimmeler, S. (2001). Number and migratory activity of circulating endothelial progenitor cells inversely correlate with risk factors for coronary artery disease. Circulation Research 89(1), E1–E7. 11440984 10.1161/hh1301.093953

[brv12866-bib-0229] Vasile, E. , Tomita, Y. , Brown, L. F. , Kocher, O. & Dvorak, H. F. (2001). Differential expression of thymosin beta‐10 by early passage and senescent vascular endothelium is modulated by VPF/VEGF: evidence for senescent endothelial cells in vivo at sites of atherosclerosis. FASEB Journal 15(2), 458–466. 11156961 10.1096/fj.00-0051com

[brv12866-bib-0230] Vemparala, K. , Roy, A. , Bahl, V. K. , Prabhakaran, D. , Nath, N. , Sinha, S. , Nandi, P. , Pandey, R. M. , Reddy, K. S. , Manhapra, A. & Lakshmy, R. (2013). Early accelerated senescence of circulating endothelial progenitor cells in premature coronary artery disease patients in a developing country—a case control study. BMC Cardiovascular Disorders 13, 104. 24245738 10.1186/1471-2261-13-104 PMC3871012

[brv12866-bib-0231] Vengrenyuk, Y. , Nishi, H. , Long, X. , Ouimet, M. , Savji, N. , Martinez, F. O. , Cassella, C. P. , Moore, K. J. , Ramsey, S. A. , Miano, J. M. & Fisher, E. A. (2015). Cholesterol loading reprograms the microRNA‐143/145‐myocardin axis to convert aortic smooth muscle cells to a dysfunctional macrophage‐like phenotype. Arteriosclerosis, Thrombosis, and Vascular Biology 35(3), 535–546. 25573853 10.1161/ATVBAHA.114.304029 PMC4344402

[brv12866-bib-0232] Voghel, G. , Thorin‐Trescases, N. , Farhat, N. , Nguyen, A. , Villeneuve, L. , Mamarbachi, A. M. , Fortier, A. , Perrault, L. P. , Carrier, M. & Thorin, E. (2007). Cellular senescence in endothelial cells from atherosclerotic patients is accelerated by oxidative stress associated with cardiovascular risk factors. Mechanisms of Ageing and Development 128(11–12), 662–671. 18022214 10.1016/j.mad.2007.09.006

[brv12866-bib-0233] Wan, J. B. , Huang, L. L. , Rong, R. , Tan, R. , Wang, J. & Kang, J. X. (2010). Endogenously decreasing tissue n‐6/n‐3 fatty acid ratio reduces atherosclerotic lesions in apolipoprotein E‐deficient mice by inhibiting systemic and vascular inflammation. Arteriosclerosis, Thrombosis, and Vascular Biology 30(12), 2487–2494. 20705919 10.1161/ATVBAHA.110.210054

[brv12866-bib-0234] Wang, L. , Sapuri‐Butti, A. R. , Aung, H. H. , Parikh, A. N. & Rutledge, J. C. (2008). Triglyceride‐rich lipoprotein lipolysis increases aggregation of endothelial cell membrane microdomains and produces reactive oxygen species. American Journal of Physiology. Heart and Circulatory Physiology 295(1), H237–H244. 18487440 10.1152/ajpheart.01366.2007 PMC2494756

[brv12866-bib-0235] Wang, L. , Gill, R. , Pedersen, T. L. , Higgins, L. J. , Newman, J. W. & Rutledge, J. C. (2009). Triglyceride‐rich lipoprotein lipolysis releases neutral and oxidized FFAs that induce endothelial cell inflammation. Journal of Lipid Research 50(2), 204–213. 18812596 10.1194/jlr.M700505-JLR200 PMC2636918

[brv12866-bib-0236] Wang, X. , Li, L. , Li, M. , Dang, X. , Wan, L. , Wang, N. , Bi, X. , Gu, C. , Qiu, S. , Niu, X. , Zhu, X. & Wang, L. (2013). Knockdown of mTOR by lentivirusmediated RNA interference suppresses atherosclerosis and stabilizes plaques via a decrease of macrophages by autophagy in apolipoprotein Edeficient mice. International Journal of Molecular Medicine 32(5), 1215–1221. 24043133 10.3892/ijmm.2013.1494

[brv12866-bib-0237] Wang, J. , Uryga, A. K. , Reinhold, J. , Figg, N. , Baker, L. , Finigan, A. , Gray, K. , Kumar, S. , Clarke, M. & Bennett, M. (2015). Vascular smooth muscle cell senescence promotes atherosclerosis and features of plaque vulnerability. Circulation 132(20), 1909–1919. 26416809 10.1161/CIRCULATIONAHA.115.016457

[brv12866-bib-0238] Wang, J. , Bai, Y. , Zhao, X. , Ru, J. , Kang, N. , Tian, T. , Tang, L. , An, Y. & Li, P. (2018a). oxLDL‐mediated cellular senescence is associated with increased NADPH oxidase p47phox recruitment to caveolae. Bioscience Reports 38(3), BSR20180283. 29695496 10.1042/BSR20180283 PMC5997791

[brv12866-bib-0239] Wang, Y. C. , Lee, A. S. , Lu, L. S. , Ke, L. Y. , Chen, W. Y. , Dong, J. W. , Lu, J. , Chen, Z. , Chu, C. S. , Chan, H. C. , Kuzan, T. Y. , Tsai, M. H. , Hsu, W. L. , Dixon, R. A. F. , Sawamura, T. , et al. (2018b). Human electronegative LDL induces mitochondrial dysfunction and premature senescence of vascular cells in vivo. Aging Cell 17(4), e12792. 29923368 10.1111/acel.12792 PMC6052487

[brv12866-bib-0240] Wang, X. , Zhang, J. Q. , Xiu, C. K. , Yang, J. , Fang, J. Y. & Lei, Y. (2020). Ginseng‐sanqi‐chuanxiong (GSC) extracts ameliorate diabetes‐induced endothelial cell senescence through regulating mitophagy via the AMPK pathway. Oxidative Medicine and Cellular Longevity 2020, 7151946. 32963699 10.1155/2020/7151946 PMC7495226

[brv12866-bib-0241] Warboys, C. M. , de Luca, A. , Amini, N. , Luong, L. , Duckles, H. , Hsiao, S. , White, A. , Biswas, S. , Khamis, R. , Chong, C. K. , Cheung, W. M. , Sherwin, S. J. , Bennett, M. R. , Gil, J. , Mason, J. C. , et al. (2014). Disturbed flow promotes endothelial senescence via a p53‐dependent pathway. Arteriosclerosis, Thrombosis, and Vascular Biology 34(5), 985–995. 24651677 10.1161/ATVBAHA.114.303415

[brv12866-bib-0242] Wassmann, S. , Stumpf, M. , Strehlow, K. , Schmid, A. , Schieffer, B. , Bohm, M. & Nickenig, G. (2004). Interleukin‐6 induces oxidative stress and endothelial dysfunction by overexpression of the angiotensin II type 1 receptor. Circulation Research 94(4), 534–541. 14699015 10.1161/01.RES.0000115557.25127.8D

[brv12866-bib-0243] Weber, C. & Noels, H. (2011). Atherosclerosis: current pathogenesis and therapeutic options. Nature Medicine 17(11), 1410–1422. 10.1038/nm.2538 22064431

[brv12866-bib-0244] Wu, Y. , Wang, Q. , Cheng, L. , Wang, J. & Lu, G. (2009). Effect of oxidized low‐density lipoprotein on survival and function of endothelial progenitor cell mediated by p38 signal pathway. Journal of Cardiovascular Pharmacology 53(2), 151–156. 19188833 10.1097/FJC.0b013e318197c637

[brv12866-bib-0245] Xiang, Q. Y. , Tian, F. , Du, X. , Xu, J. , Zhu, L. Y. , Guo, L. L. , Wen, T. , Liu, Y. S. & Liu, L. (2020a). Postprandial triglyceride‐rich lipoproteins‐induced premature senescence of adipose‐derived mesenchymal stem cells via the SIRT1/p53/Ac‐p53/p21 axis through oxidative mechanism. Aging (Albany NY) 12(24), 26080–26094. 33316776 10.18632/aging.202298 PMC7803527

[brv12866-bib-0246] Xiang, Q. Y. , Tian, F. , Lin, Q. Z. , Du, X. , Zhang, S. L. , Gui, Y. J. , Guo, L. L. , Xu, J. , Zhu, L. Y. , Wen, T. & Liu, L. (2020b). Comparison of remnant cholesterol levels estimated by calculated and measured LDL‐C levels in Chinese patients with coronary heart disease. Clinica Chimica Acta 500, 75–80. 10.1016/j.cca.2019.09.020 31655058

[brv12866-bib-0247] Xu, M. , Pirtskhalava, T. , Farr, J. N. , Weigand, B. M. , Palmer, A. K. , Weivoda, M. M. , Inman, C. L. , Ogrodnik, M. B. , Hachfeld, C. M. , Fraser, D. G. , Onken, J. L. , Johnson, K. O. , Verzosa, G. C. , Langhi, L. G. P. , Weigl, M. , et al. (2018). Senolytics improve physical function and increase lifespan in old age. Nature Medicine 24(8), 1246–1256. 10.1038/s41591-018-0092-9 PMC6082705 29988130

[brv12866-bib-0248] Yamagata, K. , Suzuki, S. & Tagami, M. (2016). Docosahexaenoic acid prevented tumor necrosis factor alpha‐induced endothelial dysfunction and senescence. Prostaglandins, Leukotrienes, and Essential Fatty Acids 104, 11–18. 26802937 10.1016/j.plefa.2015.10.006

[brv12866-bib-0249] Yan, J. , Stringer, S. E. , Hamilton, A. , Charlton‐Menys, V. , Gotting, C. , Muller, B. , Aeschlimann, D. & Alexander, M. Y. (2011). Decorin GAG synthesis and TGF‐beta signaling mediate ox‐LDL‐induced mineralization of human vascular smooth muscle cells. Arteriosclerosis, Thrombosis, and Vascular Biology 31(3), 608–615. 21205989 10.1161/ATVBAHA.110.220749

[brv12866-bib-0250] Yan, L. , Jia, Q. , Cao, H. , Chen, C. , Xing, S. , Huang, Y. & Shen, D. (2021). Fisetin ameliorates atherosclerosis by regulating PCSK9 and LOX‐1 in apoE^−/−^ mice. Experimental and Therapeutic Medicine 21(1), 25. 33262811 10.3892/etm.2020.9457 PMC7690243

[brv12866-bib-0251] Yang, D. G. , Liu, L. & Zheng, X. Y. (2008). Cyclin‐dependent kinase inhibitor p16(INK4a) and telomerase may co‐modulate endothelial progenitor cells senescence. Ageing Research Reviews 7(2), 137–146. 18343732 10.1016/j.arr.2008.02.001

[brv12866-bib-0252] Yang, D. G. , Liu, L. , Zhou, S. H. , Ma, M. F. & Wen, T. (2011). Remnant‐like lipoproteins may accelerate endothelial progenitor cells senescence through inhibiting telomerase activity via the reactive oxygen species‐dependent pathway. The Canadian Journal of Cardiology 27(5), 628–634. 21764252 10.1016/j.cjca.2010.12.075

[brv12866-bib-0253] Yang, T. , Guo, L. , Chen, L. , Li, J. , Li, Q. , Pi, Y. , Zhu, J. & Zhang, L. (2020). A novel role of FKN/CX3CR1 in promoting osteogenic transformation of VSMCs and atherosclerotic calcification. Cell Calcium 91, 102265. 32814243 10.1016/j.ceca.2020.102265

[brv12866-bib-0254] Yao, Y. , Wang, Y. , Zhang, Y. & Liu, C. (2017). Klotho ameliorates oxidized low density lipoprotein (ox‐LDL)‐induced oxidative stress via regulating LOX‐1 and PI3K/Akt/eNOS pathways. Lipids in Health and Disease 16(1), 77. 28407763 10.1186/s12944-017-0447-0 PMC5390438

[brv12866-bib-0255] Yoon, H. J. , Chay, K. O. & Yang, S. Y. (2019). Native low density lipoprotein increases the production of both nitric oxide and reactive oxygen species in the human umbilical vein endothelial cells. Genes & Genomics 41(3), 373–379. 30610621 10.1007/s13258-018-00777-4

[brv12866-bib-0256] Yoshino, J. , Baur, J. A. & Imai, S. I. (2018). NAD(+) intermediates: the biology and therapeutic potential of NMN and NR. Cell Metabolism 27(3), 513–528. 29249689 10.1016/j.cmet.2017.11.002 PMC5842119

[brv12866-bib-0257] Youk, H. , Kim, M. , Lee, C. J. , Oh, J. , Park, S. , Kang, S. M. , Kim, J. H. , Ann, S. J. & Lee, S. H. (2021). *Nlrp3*, *Csf3*, and *Edn1* in macrophage response to saturated fatty acids and modified low‐density lipoprotein. Korean Circulation Journal 51(1), 68–80. 32975056 10.4070/kcj.2020.0117 PMC7779813

[brv12866-bib-0258] Yu, E. , Calvert, P. A. , Mercer, J. R. , Harrison, J. , Baker, L. , Figg, N. L. , Kumar, S. , Wang, J. C. , Hurst, L. A. , Obaid, D. R. , Logan, A. , West, N. E. , Clarke, M. C. , Vidal‐Puig, A. , Murphy, M. P. , et al. (2013). Mitochondrial DNA damage can promote atherosclerosis independently of reactive oxygen species through effects on smooth muscle cells and monocytes and correlates with higher‐risk plaques in humans. Circulation 128(7), 702–712. 23841983 10.1161/CIRCULATIONAHA.113.002271

[brv12866-bib-0259] Yubero‐Serrano, E. M. , Fernandez‐Gandara, C. , Garcia‐Rios, A. , Rangel‐Zuniga, O. A. , Gutierrez‐Mariscal, F. M. , Torres‐Pena, J. D. , Marin, C. , Lopez‐Moreno, J. , Castano, J. P. , Delgado‐Lista, J. , Ordovas, J. M. , Perez‐Martinez, P. & Lopez‐Miranda, J. (2020). Mediterranean diet and endothelial function in patients with coronary heart disease: an analysis of the CORDIOPREV randomized controlled trial. PLoS Medicine 17(9), e1003282. 32903262 10.1371/journal.pmed.1003282 PMC7480872

[brv12866-bib-0260] Zampetaki, A. , Kirton, J. P. & Xu, Q. (2008). Vascular repair by endothelial progenitor cells. Cardiovascular Research 78(3), 413–421. 18349136 10.1093/cvr/cvn081

[brv12866-bib-0261] Zhang, X. , Qi, R. , Xian, X. , Yang, F. , Blackstein, M. , Deng, X. , Fan, J. , Ross, C. , Karasinska, J. , Hayden, M. R. & Liu, G. (2008). Spontaneous atherosclerosis in aged lipoprotein lipase‐deficient mice with severe hypertriglyceridemia on a normal chow diet. Circulation Research 102(2), 250–256. 18032735 10.1161/CIRCRESAHA.107.156554

[brv12866-bib-0262] Zhang, E. , Guo, Q. , Gao, H. , Xu, R. , Teng, S. & Wu, Y. (2015). Metformin and resveratrol inhibited high glucose‐induced metabolic memory of endothelial senescence through SIRT1/p300/p53/p21 pathway. PLoS One 10(12), e0143814. 26629991 10.1371/journal.pone.0143814 PMC4668014

[brv12866-bib-0263] Zhang, B. C. , Zhang, C. W. , Wang, C. , Pan, D. F. , Xu, T. D. & Li, D. Y. (2016a). Luteolin attenuates foam cell formation and apoptosis in ox‐LDL‐stimulated macrophages by enhancing autophagy. Cellular Physiology and Biochemistry 39(5), 2065–2076. 27825167 10.1159/000447902

[brv12866-bib-0264] Zhang, H. , Ryu, D. , Wu, Y. , Gariani, K. , Wang, X. , Luan, P. , D'Amico, D. , Ropelle, E. R. , Lutolf, M. P. , Aebersold, R. , Schoonjans, K. , Menzies, K. J. & Auwerx, J. (2016b). NAD(+) repletion improves mitochondrial and stem cell function and enhances life span in mice. Science 352(6292), 1436–1443. 27127236 10.1126/science.aaf2693

[brv12866-bib-0265] Zhang, J. J. , Liu, W. Q. , Peng, J. J. , Ma, Q. L. , Peng, J. & Luo, X. J. (2017). miR‐21‐5p/203a‐3p promote ox‐LDL‐induced endothelial cell senescence through down‐regulation of mitochondrial fission protein Drp1. Mechanisms of Ageing and Development 164, 8–19. 28347692 10.1016/j.mad.2017.03.009

[brv12866-bib-0266] Zhang, J. J. , Zhang, Y. Z. , Peng, J. J. , Li, N. S. , Xiong, X. M. , Ma, Q. L. , Luo, X. J. , Liu, B. & Peng, J. (2018). Atorvastatin exerts inhibitory effect on endothelial senescence in hyperlipidemic rats through a mechanism involving down‐regulation of miR‐21‐5p/203a‐3p. Mechanisms of Ageing and Development 169, 10–18. 29248491 10.1016/j.mad.2017.12.001

[brv12866-bib-0267] Zhang, H. , Yang, X. , Pang, X. , Zhao, Z. , Yu, H. & Zhou, H. (2019). Genistein protects against ox‐LDL‐induced senescence through enhancing SIRT1/LKB1/AMPK‐mediated autophagy flux in HUVECs. Molecular and Cellular Biochemistry 455(1–2), 127–134. 30443855 10.1007/s11010-018-3476-8

[brv12866-bib-0268] Zhi, K. , Li, M. , Bai, J. , Wu, Y. , Zhou, S. , Zhang, X. & Qu, L. (2016). Quercitrin treatment protects endothelial progenitor cells from oxidative damage via inducing autophagy through extracellular signal‐regulated kinase. Angiogenesis 19(3), 311–324. 27017346 10.1007/s10456-016-9504-y

[brv12866-bib-0269] Zhong, J. K. , Guo, Z. G. , Li, C. , Wang, Z. K. , Lai, W. Y. & Tu, Y. (2011). Probucol alleviates atherosclerosis and improves high density lipoprotein function. Lipids in Health and Disease 10, 210. 22078494 10.1186/1476-511X-10-210 PMC3253062

[brv12866-bib-0270] Zhou, Y. D. , Cao, X. Q. , Liu, Z. H. , Cao, Y. J. , Liu, C. F. , Zhang, Y. L. & Xie, Y. (2016). Rapamycin inhibits oxidized low density lipoprotein uptake in human umbilical vein endothelial cells via mTOR/NF‐kappaB/LOX‐1 pathway. PLoS One 11(1), e0146777. 26752047 10.1371/journal.pone.0146777 PMC4709184

[brv12866-bib-0271] Zhou, Z. , Zhu, X. , Yin, R. , Liu, T. , Yang, S. , Zhou, L. , Pan, X. & Ma, A. (2020). K63 ubiquitin chains target NLRP3 inflammasome for autophagic degradation in ox‐LDL‐stimulated THP‐1 macrophages. Aging (Albany NY) 12(2), 1747–1759. 32003754 10.18632/aging.102710 PMC7053591

[brv12866-bib-0272] Zhu, H. , Wang, Z. , Dong, Z. , Wang, C. , Cao, Q. , Fan, F. , Zhao, J. , Liu, X. , Yuan, M. , Sun, X. , Peng, X. , Zou, Y. , Zhou, J. , Ge, J. , Zhou, X. , et al. (2019). Aldehyde dehydrogenase 2 deficiency promotes atherosclerotic plaque instability through accelerating mitochondrial ROS‐mediated vascular smooth muscle cell senescence. Biochimica et Biophysica Acta. Molecular Basis of Disease 1865(7), 1782–1792. 30315930 10.1016/j.bbadis.2018.09.033

[brv12866-bib-0273] Zu, Y. , Liu, L. , Lee, M. Y. , Xu, C. , Liang, Y. , Man, R. Y. , Vanhoutte, P. M. & Wang, Y. (2010). SIRT1 promotes proliferation and prevents senescence through targeting LKB1 in primary porcine aortic endothelial cells. Circulation Research 106(8), 1384–1393. 20203304 10.1161/CIRCRESAHA.109.215483

